# A multichaperone condensate enhances protein folding in the endoplasmic reticulum

**DOI:** 10.1038/s41556-025-01730-w

**Published:** 2025-08-11

**Authors:** Anna Leder, Guillaume Mas, Viktória Szentgyörgyi, Roman P. Jakob, Timm Maier, Anne Spang, Sebastian Hiller

**Affiliations:** Biozentrum, University of Basel, Basel, Switzerland

**Keywords:** Chaperones, Endoplasmic reticulum, NMR spectroscopy

## Abstract

Protein folding in the endoplasmic reticulum (ER) relies on a network of molecular chaperones that facilitates the folding and maturation of client proteins. How the ER chaperones organize in a supramolecular manner to exert their cooperativity has, however, remained unclear. Here we report the discovery of a multichaperone condensate in the ER lumen, which is formed around the chaperone PDIA6 during protein folding homeostasis. The condensates form in a Ca^2+^-dependent manner and we resolve the underlying mechanism at the atomic and cellular levels. The PDIA6 condensates recruit further chaperones—Hsp70 BiP, J-domain protein ERdj3, disulfide isomerase PDIA1 and Hsp90 Grp94—which constitute some of the essential components of the early folding machinery. The chaperone condensates enhance folding of proteins, such as proinsulin, and prevent protein misfolding in the ER lumen. The PDIA6-scaffolded chaperone condensates hence provide the functional basis for spatial and temporal coordination of the dynamic ER chaperone network.

## Abstract (web summary)

Leder et al. show that the chaperones PDIA6, Hsp70 BiP, ERdj3, PDIA1 and Hsp90 form co-condensates within the endoplasmic reticulum, enhancing folding and preventing misfolding of client proteins.

## Main

One-third of eukaryotic proteins are processed within the endoplasmic reticulum (ER) to obtain their correct structure^1–3^. This function is ensured by a network of molecular chaperones that recognizes client proteins and assists their folding^4,5^. A rate-limiting step in ER protein folding is the formation of disulfide bonds, and the protein disulfide isomerase (PDI) chaperone family is therefore indispensable for folding homeostasis^6,7^. PDIs contain one or more catalytically active thioredoxin-like domains, which can reduce, oxidize or isomerize disulfide bonds in client proteins via a characteristic CXXC motif. They typically combine catalytically active and inactive domains towards multidomain architectures. Although all PDIs share common structural features, they differ in terms of client-protein specificity and affinity, chaperone activity and redox properties, resulting in specialized roles for individual family members^8^. One particular family member, PDIA6, caught our attention because its malfunction is connected to several human diseases including cancer and diabetes, yet its exact function remained elusive^9–12^.

## PDIA6 is organized in phase-separated condensates

**Fig. 1:**
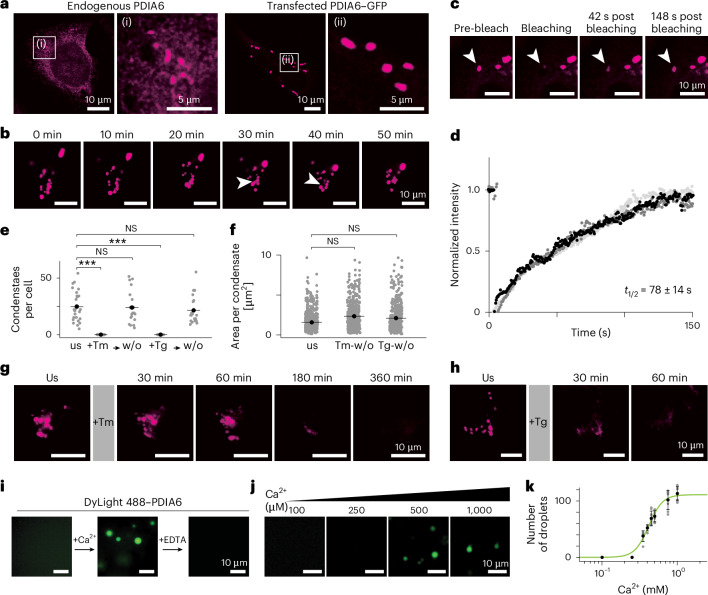
PDIA6 forms phase-separated condensates that dissolve in response to ER stress. **a**, Fixed HeLa cells expressing endogenous PDIA6 (left) and live-cell images of HeLa cells transfected with PDIA6–GFP (right). Endogenous PDIA6 was stained with anti-PDIA6 and overexpressed PDIA6–GFP was visualized by imaging eGFP. **b**, Time series of a HeLa cell transiently transfected with PDIA6–GFP. A fusion event is indicated by arrowheads. **c**, PDIA6–GFP FRAP experiment in HeLa cells. **d**, Fluorescence recovery analysis of the PDIA6–GFP FRAP experiment presented in **c**. In vivo analysis of three cells from three independent experiments each coloured in a different shade of grey. The black trace corresponds to Supplementary Video 3. **e**, Number of condensates in unstressed cells as well as cells subjected to tunicamycin or thapsigargin treatment and subsequent washouts. A total of 622, 0, 449, 0 and 455 condensates were observed in *n* = 25, 24, 21, 25 and 20 cells, respectively, from left to right, pooled from three independent biological replicates. *P* = 2.33 × 10^−10^, *P* = 0.337, *P* = 9.65 × 10^−11^ and *P* = 0.785 (left to right); two-sided unpaired Mann–Whitney test. Tg, thapsigargin stress; Tm, tunicamycin stress; us, unstressed; w/o, washout. **f**, Condensate size in unstressed and recovered cells after treatment with tunicamycin or thapsigargin; *n* = 25, 21 and 20 cells, respectively, pooled from three independent biological replicates. *P* = 0.226 (left) and *P* = 0.464 (right); two-sided unpaired Mann–Whitney test. **g**,**h**, Time series images of individual HeLa cells transiently transfected with PDIA6–GFP during tunicamycin (**g**) and thapsigargin (**h**) treatment. **i**, In vitro phase separation of DyLight 488–PDIA6 in the presence of crowding agent (left), crowding agent plus 10 mM Ca^2+^ (middle) and crowding agent plus 10 mM Ca^2+^ and 20 mM EDTA (right). **j**, In vitro phase separation of DyLight 488–PDIA6 in the presence of crowding agent with different concentrations of Ca^2+^, as indicated. **k**, Number of in vitro PDIA6 droplets per field of view in the presence of crowding agent at different Ca^2+^ concentrations and fitted Hill equation binding model (green slope). Data are the mean ± s.d.; *n* = 9 images for [Ca^2+^] ≥ 500 μM and *n* = 6 images for [Ca^2+^] < 500 μM per condition, pooled from three independent experiments. All images are representative of at least three independent experiments. ***P < 0.001; NS, not significant. Source data

**Extended Data Fig. 1:**
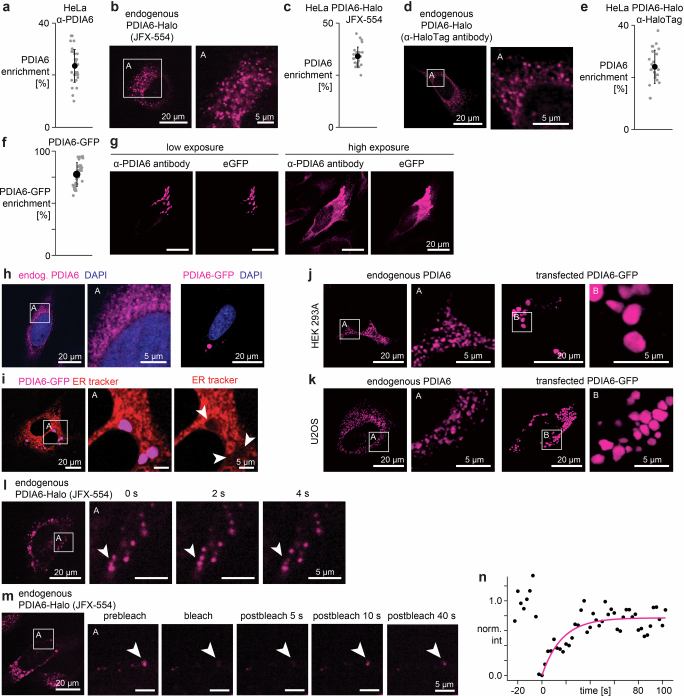
In vivo phase separation of PDIA6. **a**, Quantification of endogenous PDIA6 enrichment in condensates visualized by anti-PDIA6 antibody. Mean and standard deviation (*n* = 25 cells, pooled across three independent experiments). A total of 2,300 condensates were counted. **b**, PDIA6 was endogenously tagged with HaloTag in HeLa cells (PDIA6^EN^-Halo). Live-cell imaging of HeLa cells stably PDIA6^EN^-Halo fusion. PDIA6 was visualized by staining with the HaloTag ligand JFX-554. Scale bar, 20 μm, inset 5 μm. **c**, Quantification of PDIA6^EN^-Halo enrichment in condensates of live cells. PDIA6 was visualized by the HaloTag ligand JFX-554. Mean and standard deviation (*n* = 25 cells, pooled across three independent experiments). A total of 2,320 condensates were counted. **d**, Fixed HeLa cells stably expressing PDIA6^EN^-Halo fusion. PDIA6 stained with anti-HaloTag antibody. **e**, Quantification of PDIA6^EN^-Halo enrichment in condensates of fixed cell images. PDIA6 was stained by an α-Halo-antibody. Mean and standard deviation (*n* = 25 cells, pooled across three independent experiments). A total of 2,650 condensates were counted. **f**, Quantification of transiently transfected PDIA6–GFP enrichment in condensates visualized by the eGFP signal. Mean and standard deviation (*n* = 31 cells, pooled across three independent experiments). A total of 720 condensates were counted. **g**, Fixed HeLa cells transfected with PDIA6–GFP (eGFP signal) and stained against PDIA6 (anti-PDIA6 antibody, imaged in red channel) recorded at low (left) and high exposure (right). Scale bar, 20 μm. **h**, Fixed HeLa cells expressing endogenous PDIA6 (pink, left) and transfected with PDIA6–GFP (pink, right) stained for DAPI (blue). PDIA6–GFP was visualized by imaging eGFP. Scale bar, 20 μm, insert 5 μm. **i**, Live-cell imaging of HeLa cells transfected with PDIA6–GFP (pink), treated with ER tracker (red). The white arrows indicate the position of PDIA6 condensates. Scale bar, 20 μm, insert 5 μM. **j**, Fixed HEK293A cells expressing endogenous PDIA6 (left) and live-cell imaging of HEK293A cells transfected with PDIA6–GFP (right). Endogenous PDIA6 was stained with an anti-PDIA6 antibody, overexpressed PDIA6–GFP was visualized by imaging eGFP. Scale bar, 20 μm, insert 5 μm. **k**, Fixed U2OS cells expressing endogenous PDIA6 (left) and live-cell imaging of U2OS cells transfected with PDIA6–GFP (right). Endogenous PDIA6 was stained with an anti-PDIA6 antibody, overexpressed PDIA6–GFP was visualized by imaging eGFP. Scale bar, 20 μm, insert 5 μm. **l**, Time series of a single HeLa cell stably expressing PDIA6^EN^-Halo fusion. Scale bar, 20 μm, inset 5 μm. **m**, FRAP experiment of PDIA6^EN^-Halo. Images were not bleach-corrected **n**, Fluorescence recovery analysis of PDIA6^EN^-Halo FRAP experiment presented in **m**. Representative data of three independent replicates. Source data

We observed that endogenous PDIA6 localized as expected in the ER of human HeLa cells. Remarkably, however, the endogenous protein was not homogeneously dispersed but concentrated in well-defined clusters, both in wild-type (WT) cells as well as in PDIA6–Halo knock-in cells (Fig. 1a and Extended Data Fig. 1a–e). Overexpressed PDIA6 formed clusters of similar shape and localization in living cells, albeit with substantially increased size (Fig. 1a and Extended Data Fig. 1f–i). PDIA6 formed similar clusters in HEK and U2OS cells (Extended Data Fig. 1j,k). The PDIA6 clusters were mobile within the ER and underwent fission and fusion events, and their fluorescence recovery after photobleaching (FRAP) occurred within minutes (half life (*t*_1/2_) = 78 ± 14 s, diffusion coefficient (*D*) = 4 ± 1 × 10^−3^ μm^2^ s^−1^), a time scale that is common for biological phase-separated condensates^13,14^ (Fig. 1b–d, Extended Data Fig. 1l–n and Supplementary Videos 1–4). The PDIA6 condensates thus fulfil all hallmarks of biological phase separation^15^.

## PDIA6 condensates disperse in response to ER stress

**Extended Data Fig. 2:**
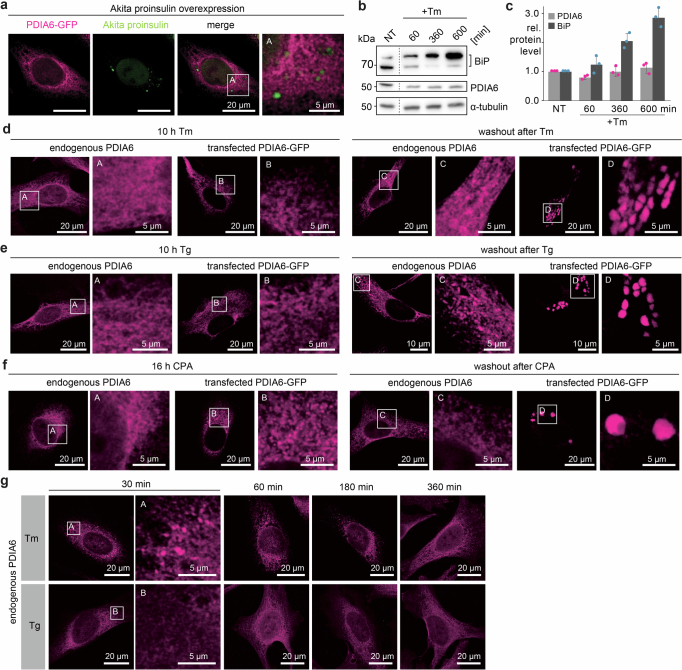
Effect of ER stress on PDIA6 condensates. **a**, Fixed HeLa cells co-transfected with PDIA6–GFP (pink) and Akita proinsulin-myc (green). Akita proinsulin, a folding-defective mutant of proinsulin^18^, was used to mimic the accumulation unfolded/misfolded proteins in the ER lumen. Akita proinsulin forms puncta, as previously reported^61^. PDIA6 was visualized by imaging eGFP, Akita proinsulin using an anti-myc antibody. Scale bar, 20 μm, insert 5 μm. **b**, Western blot showing expression levels of PDIA6, BiP and α-tubulin and at indicated time points of Tm treatment in HeLa cells. Unprocessed blots are available. Representative blot of three independent experiments. **c**, Quantification of PDIA6 and BiP levels from blot shown in **b**. Data representative of an experimental triplicate (*n* = 3). Mean and standard deviation. **d**–**f**, Fixed HeLa cells expressing endogenous PDIA6 and live-cell imaging of HeLa cells transfected with PDIA6–GFP after 10 h of Tm treatment (**d**), 10 h of Tg treatment (**e**) or 16 h of CPA (reversible Tg analogue) treatment (**f**) (left) and 24 h after drug washout (right). Endogenous PDIA6 was stained with an anti-PDIA6 antibody, overexpressed PDIA6–GFP was visualized by imaging eGFP. **g**, Fixed HeLa cells expressing endogenous PDIA6 observed at different time points after Tm (top) and Tg treatment (bottom). Source data

Attempts to delete the *PDIA6* gene in human cells were unsuccessful, indicating that PDIA6 is an essential protein, consistent with earlier findings in *Caenorhabditis* *elegans*^16^. Intriguingly, PDIA6 malfunction has been reported to lead to an imbalance in ER folding homeostasis^16,17^. We thus wondered whether a functional coupling exists between ER stress and condensate formation. To create a physiological ER-stress situation, we overexpressed Akita proinsulin, a misfolded mutant of proinsulin that accumulates in the ER and thereby causes ER stress^18^. This led to homogenous dispersion of PDIA6 in the entire ER lumen (Extended Data Fig. 2a). Similarly, following treatment of the cells with the pharmacological ER-stress inducer tunicamycin, the PDIA6 condensates dispersed independently of the PDIA6 expression levels (Extended Data Fig. 2b–d). Notably, the stress-dependent condensate dissolution was reversible, as PDIA6 condensates reorganized after washout of the stressor (Fig. 1e,f). Two other stress inducers, thapsigargin and cyclopiazonic acid, induced the same effect (Fig. 1e,f and Extended Data Fig. 2e,f). Remarkably, however, the dissolution kinetics differed substantially between the compounds. PDIA6 condensates dissolved completely within 1 h following thapsigargin treatment, whereas dissolution was observed only after 6 h of tunicamycin treatment (Fig. 1g,h and Extended Data Fig. 2g). This kinetic difference correlates with the different modes of action of the two drugs. Thapsigargin blocks the sarco-/endoplasmic reticulum Ca^2+^ ATPase pumps^19^, leading to an almost immediate Ca^2+^ depletion in the ER lumen, whereas tunicamycin inhibits *N*-glycosylation of newly synthesized proteins^20^ and causes a delayed drop in Ca^2+^ levels as a secondary effect of the accumulation of unfolded proteins^21^. Overall, the presence of PDIA6 condensates is thus coupled to protein homeostatic conditions and their dissolution in response to ER stress seems to be regulated by luminal Ca^2+^ levels.

**Extended Data Fig. 3:**
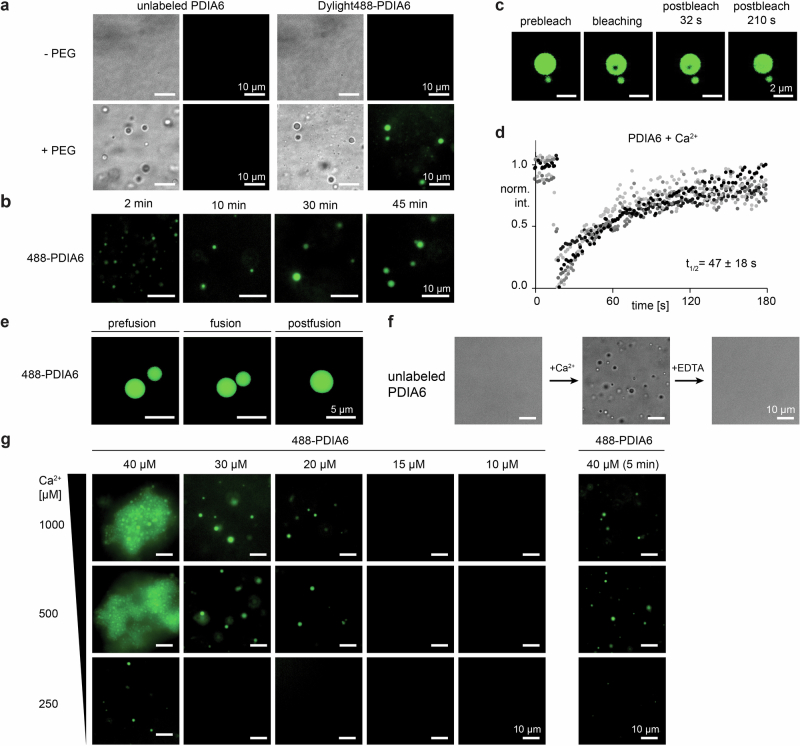
In vitro reconstitution of PDIA6 droplets. **a**, In vitro phase separation of PDIA6 (left) and Dylight 488–PDIA6 (right) with 10 mM Ca^2+^ in absence (top) and presence (bottom) of 10% PEG3350. Scale bar, 10 μm. **b**, In vitro time evolution of Dylight 488–PDIA6 droplets in presence of 10 mM Ca^2+^. Scale bar, 10 μm. **c**, FRAP experiments of Dylight 488–PDIA6 in presence of 10 mM Ca^2+^. Scale bar, 2 μm. **d**, Fluorescence recovery analysis of the Dylight 488–PDIA6 FRAP experiments presented in **c**. Experimental triplicate in shades of grey. **e**, Fusion event of Dylight 488–PDIA6 in presence of crowding agent and 10 mM Ca^2+^. Scale bar, 5 μm. **f**, In vitro phase separation of unlabelled PDIA6 in presence of crowding agent in absence (left) and presence of 10 mM Ca^2+^ (middle), and in presence of 10 mM Ca^2+^ and 20 mM EDTA (right). Scale bar, 10 μm. **g**, In vitro phase separation of Dylight 488–PDIA6 at different concentrations in presence of crowding agent and different Ca^2+^ concentrations after 30 min (left) and after 5 min (right), as indicated. Scale bar, 10 μm. Source data

To test this mechanistic hypothesis, we reconstituted droplets of purified recombinant PDIA6 protein in vitro. Homeostatic ER was mimicked by a high Ca^2+^ concentration, reducing conditions, physiological pH and a crowding agent. Under these conditions, recombinant PDIA6 reproducibly formed droplets that underwent fusion events and time-dependent growth in a time scale of seconds. The protein dynamically exchanged between the condensed and dispersed phase with similar kinetics as in vivo, as determined by FRAP experiments (*t*_1/2_ = 47 ± 18 s, *D* = 6 ± 2 × 10^−3^ μm^2^ s^−1^; Fig. 1i and Extended Data Fig. 3a–f). Droplets did not form when Ca^2+^ was replaced by another divalent ion, Mg^2+^, that is, their formation was Ca^2+^-specific. Importantly, the droplets formed only at Ca^2+^ concentrations above about 500 μM and the saturation concentration was independent of the protein concentration (Fig. 1j and Extended Data Fig. 3g). We determined the Ca^2+^ affinity of PDIA6 to be 420 ± 20 μM in vitro by quantifying the number of PDIA6 condensates as a function of Ca^2+^ concentration (Fig. 1k). Remarkably, this value corresponds to the physiological transition between protein folding homeostasis ([Ca^2+^]_homeo_ ≈ 800 μM) and ER stress ([Ca^2+^]_stress_ ≈ 100 μM)^22^, and therefore correlates very well with our observations in living cells. PDIA6 condensates are thus regulated by the Ca^2+^ concentration.

## Structural characterization and domain organization of PDIA6

**Fig. 2:**
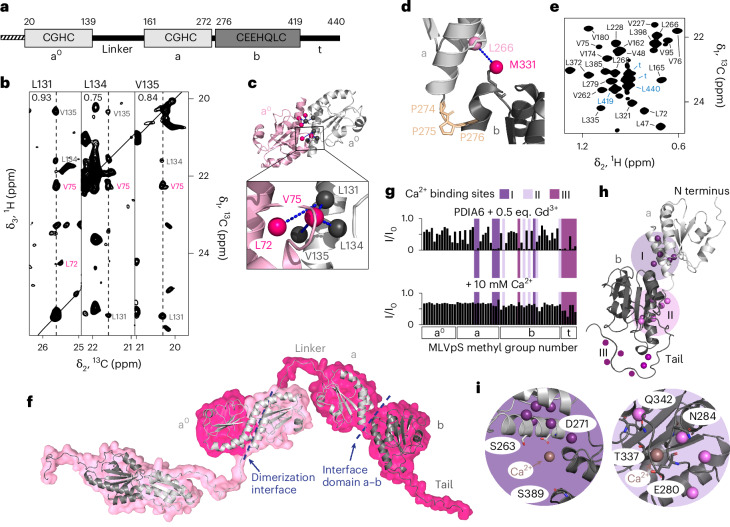
Structural characterization of PDIA6. **a**, Domain organization of PDIA6 including the cleaved signalling sequence (dashed), delimitations for domains a^0^, a and b, and the C-terminal tail (t). **b**, Two-dimensional strips of a three-dimensional ^13^C, ^13^C-resolved [^1^H, ^1^H]-NOESY spectrum of 1 mM PDIA6. The proton frequency is shown in the top-left corner of each strip. Intramolecular (grey) and intermolecular (pink) NOEs are indicated. **c**, Crystal structure of PDIA6 domain a^0^ (PDB: 4ef0) with subunits coloured in pink and grey. The NOE network detected in **b** is indicated by dashed blue lines. **d**, Interface between domain a (light grey) and b (dark grey). Proline residues in the linker are highlighted in gold. The interdomain NOE contact between L266 in domain a and M331 in domain b is represented by a blue line. **e**, Section of the two-dimensional [^13^C,^1^H]-heteronuclear multiple quantum correlation (HMQC) spectrum of 100 μM methyl-labelled PDIA6. Signals of residues in the tail are highlighted in blue. **f**, Structural model of dimeric PDIA6 full-length (PDB: a^0^, 4ef0; a, 4gwr; b, 8cpq). **g**, Paramagnetic relaxation enhancement effect of 0.5 equivalent (eq.) Gd^3+^ on methyl groups of 100 μM methyl-labelled PDIA6 WT (top) and outcompetition by Ca^2+^ (bottom). Residues with substantial loss in intensity are highlighted in shades of purple. **h**, Structural model of PDIA6 domain a (light grey) and b (dark grey) representing the methyl groups identified in **g**. **i**, Ca^2+^ binding sites I (left) and II (right). Ca^2+^ ions are modelled based on calsequestrin (PDB: 5kn3; Extended Data Fig. 6d,e). Methyl groups with substantial loss in intensity in **g** are represented as spheres. Side chains of Ca^2+^ binding residues are shown as sticks.

**Extended Data Fig. 4:**
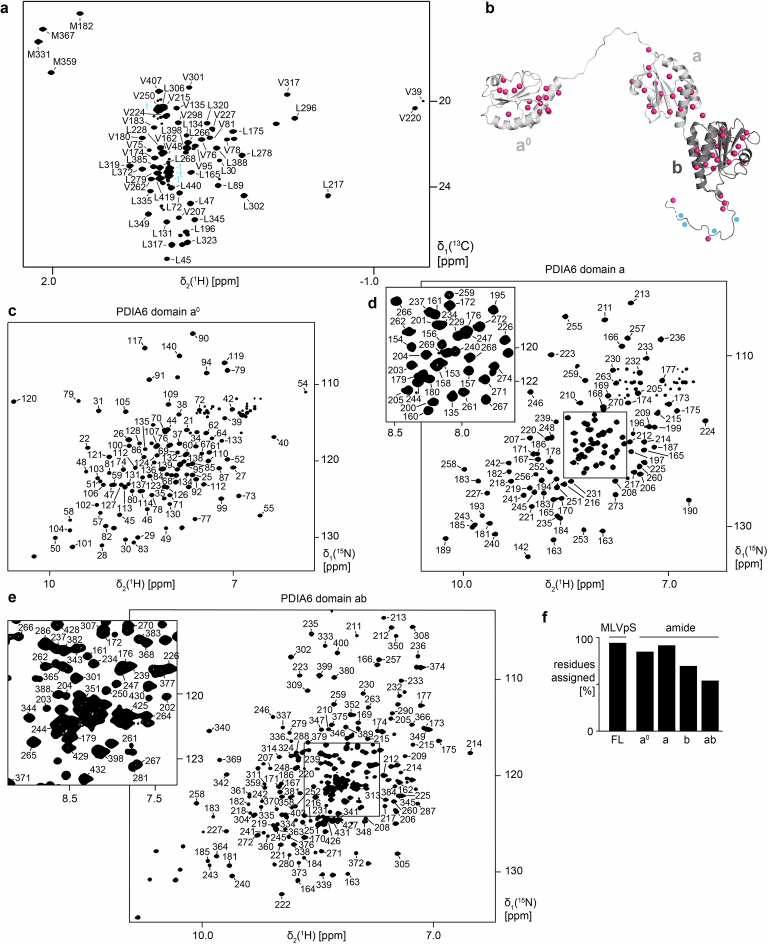
PDIA6 methyl group and amide backbone NMR assignments. **a**, 2D [^13^C,^1^H]-HMQC spectrum of methyl-labelled PDIA6. Assignment of Met[^13^C^1^H]^ε^, Val-[^13^C^1^H]^γ2^ and Leu-[^13^C^1^H]^δ2^ methyl groups are indicated. Peaks annotated with a “t” correspond to residues in the C-terminal tail which could not be unambiguously assigned. **b**, Assigned methyl groups (pink spheres) displayed on the structure of PDIA6 established in Fig. 2. Blue spheres represent partially assigned residues in the C-terminal tail. **c**–**e**, 2D [^15^N,^1^H]-HSQC spectra of PDIA6 domain a^0^ (**c**), domain a (**d**) and domain ab (**e**). Sequence-specific resonance assignments are indicated. **f**, Percentages of assigned residues of methyl and amide groups in PDIA6 FL and domain constructs.

**Extended Data Fig. 5:**
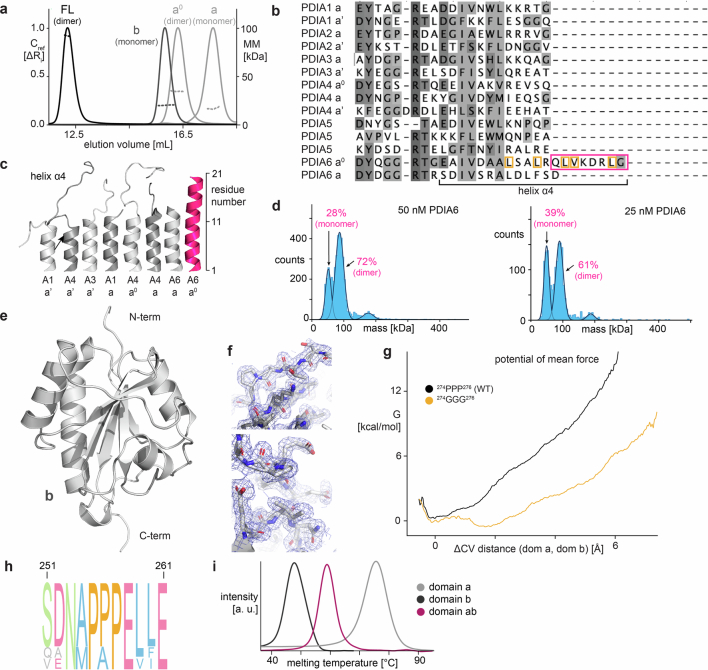
Domain characterization of PDIA6. **a**, SEC elution profiles (solid lines, left axis) and MALS apparent molecular mass (dotted lines, right axis) of PDIA6 full-length (FL) and domains. **b**, Sequence alignment of helix α4 of PDI catalytically active domains. The extended helix α4 of PDIA6 is highlighted by a pink square. Residues forming the leucine-valine adhesive dimerization motif^23^ are marked by an orange square. Domain delimitations as defined on uniport.org or by available crystal structures. **c**, Structural comparison of PDI catalytically active domains helix α4. **d**, Mass photometry of PDIA6 at 50 nM and 25 nM. Occupancy and oligomeric state are indicated for each peak. **e**, Crystal structure of PDIA6 domain b at a resolution of 1.8 Å. **f**, Zoom on the electron density map of PDIA6 domain b crystal structure. **g**, Potential of mean force along the collective variable (CV). The free energy profile for the WT protein (^274^PPP^276^) has one minimum at ΔCV 0 (black), while the ^274^GGG^276^ linker is flexible in the 0–2 Å range and the free energy increases slower with CV distance (yellow). **h**, Protein logo analysis of PDIA6 of domain a-b linker of *Homo sapiens*, *Rattus norvegicus*, *Mus musculus*, *Pongo abelii*, *Drosophila melanogaster* and *Caenorhabditis elegans*. **i**, Thermal stability of reduced domain a (light grey), domain b (dark grey) and domain ab (pink)as determined by differential scanning fluorimetry.

We then employed an integrated structural biology approach to resolve the structure and mechanism of PDIA6 condensate formation. PDIA6 consists of two catalytically active thioredoxin-like domains, domains a^0^ and a, which are followed by the inactive domain b of unknown function (Fig. 2a)^23,24^. To monitor conformational changes and interactions using NMR spectroscopy, we assigned 94% of the Met^ε^, Leu^δ2^ and Val^γ2^ methyl groups and established near-complete amide backbone assignments (Extended Data Fig. 4). Through size-exclusion chromatography-multi-angle light scattering (SEC-MALS) and nuclear Overhauser effect (NOE) spectroscopy (NOESY) experiments, we found that PDIA6 dimerizes via helix α4 in domain a^0^ (Fig. 2b,c and Extended Data Fig. 5a), in agreement with previous findings^25^. The dimerization interface was resolved by a methyl NOE network between residues L72 and L134 as well as between V75 and L131, L134 and L135, which corresponds to the arrangement observed in crystals of domain a^0^ (Protein Data Bank (PDB) identifier: 4ef0). The dimerization thus resulted from a carboxy-terminal extension of helix α4 in domain a^0^ by seven residues and contrasts the PDI family members PDIA1–5, which have shorter helices and are monomeric^26^ (Extended Data Fig. 5b,c). Dimerization occurred with a dissociation constant (*K*_D_) in the low nanomolar range (Extended Data Fig. 5d) and the dimer is therefore the dominant species at the physiological concentration of about 100 μM (refs. ^27,28^). We determined the crystal structure of domain b (residues 274–427), which adopts a thioredoxin-like fold, to a resolution of 1.8 Å (Extended Data Fig. 5e,f and Supplementary Table 1).

The dimeric domain a^0^ is connected to domain a by a long positively charged linker, and domains a and b are connected by a short five-residue linker that presumably restricts the relative domain motions as it contains three conserved proline residues (Fig. 2d and Extended Data Fig. 5g,h). Domains a and b unfold cooperatively, suggesting that they are stabilized by interdomain interactions (Extended Data Fig. 5i). The C-terminal tail of PDIA6 (residues 420–440) is highly flexible and disordered, as evidenced by a narrow chemical shift dispersion and high NMR signal intensities (Fig. 2e). Combining the experimental data, we established a full atomic model of PDIA6 (Fig. 2f).

## PDIA6 has three distinct Ca^2+^ binding sites

**Extended Data Fig. 6:**
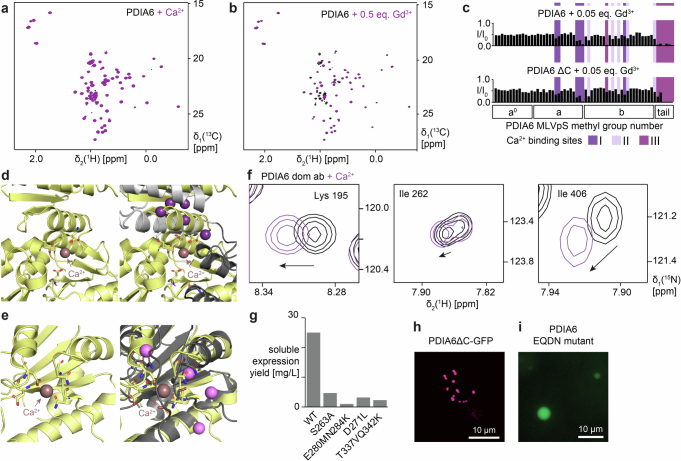
Localization of three Ca^2+^ binding sites in PDIA6. **a**, Overlay of 2D [^13^C,^1^H]-HMQC spectra of 100 μM methyl-labelled PDIA6 in absence (black) and presence of 10 mM Ca^2+^ (purple). Full spectra shown in Supplementary Fig. 3a. **b**, Overlay of 2D [^13^C,^1^H]-HMQC spectra of 100 μM methyl-labelled PDIA6 in absence (black) and presence of 0.05 eq. Gd^3+^ (purple). Full spectra shown in Supplementary Fig. 3b. **c**, PRE-effect of 0.05 eq. Gd^3+^ on full-length PDIA6 (top) and truncated ΔC PDIA6 (middle) and outcompetition of 0.5 eq. Gd^3+^ by 1 mM Ca^2+^ on full-length PDIA6 (bottom). Residues with substantial loss in intensity are highlighted in shades of purple (I, II and III). **d**,**e**, Zoom on Ca^2+^ binding site of calsequestrin low Ca^2+^ form (PDB: 5kn3, yellow) (left) and overlay with PDIA6 (grey) Ca^2+^ binding sites I (**d**) and II (**e**) (right). Side chains of Ca^2+^ binding residues shown as sticks. Methyl groups with substantial loss in intensity in Fig. 2g represented as spheres as in Fig. 2h. The Ca^2+^ ions in calsequestrin are coordinated by the amino acid residues Asp/Glu/Asp for site I and Asn/Asn/Asp/Asp for site II, while in PDIA6 by Asp/Ser/Ser and Asn/Glu/Gln/Thr, respectively, resulting in weaker Ca^2+^ binding. **f**, Overlayed sections of 2D [^15^N,^1^H]-HSQC spectra of 100 μM PDIA6 domain ab in absence (black) and in presence of excess Ca^2+^ showing assigned residues located at Ca^2+^ binding sites. Full spectra in Supplementary Fig. 4. **g**, Soluble expression yields of PDIA6 constructs with mutations (S263A, D271L, E280M, N284K, T337V or Q342K) targeting the Ca^2+^ binding sites. **h**, Fixed HeLa cells transfected with PDIA6–GFP ΔC. Scale bar, 10 μm. **i**, In vitro phase separation of Dylight 488–PDIA6 EQDN, which has been reported to not bind Ca^2+^ at the C-terminal tail^23^. Scale bar, 10 μm.

As PDIA6 condensate formation is Ca^2+^-dependent, we set out to determine PDIA6 Ca^2+^ binding sites. Titration of Ca^2+^ to PDIA6 did not lead to substantial changes in the methyl NMR spectra, demonstrating the absence of large structural rearrangements (Extended Data Fig. 6a). Titration of the paramagnetic analogue Gd^3+^, however, quenched NMR signals specifically in three structural regions, thus revealing three cation binding sites (I, II and III; Fig. 2g,h and Extended Data Fig. 6b,c). Bound Gd^3+^ was outcompeted by an excess of Ca^2+^, proving Ca^2+^ binding specificity. Binding sites I and II are located at the domain a–b interface and in domain b, respectively. Binding site III is located within the disordered C-terminal tail, as previously proposed^25^. We pinpointed the exact locations of binding sites I and II in the centre of the quenched regions by structural alignment with the Ca^2+^ binding protein calsequestrin and amide NMR experiments (Fig. 2i and Extended Data Fig. 6d–f)^29^. We attempted to probe the binding sites experimentally, however, single point mutations in binding sites I or II resulted in drastically decreased protein yields, preventing further experiments (Extended Data Fig. 6g). In contrast, mutagenesis of the Ca^2+^ binding site III was readily possible. Disruption of this site did not affect condensate formation in vitro or in living cells (Extended Data Fig. 6h,i). Taken together, the Ca^2+^-dependent phase separation of PDIA6 is controlled by a molecular switch in domain b, which is activated following Ca^2+^ binding.

## A molecular switch regulates condensate formation

**Extended Data Fig. 7:**
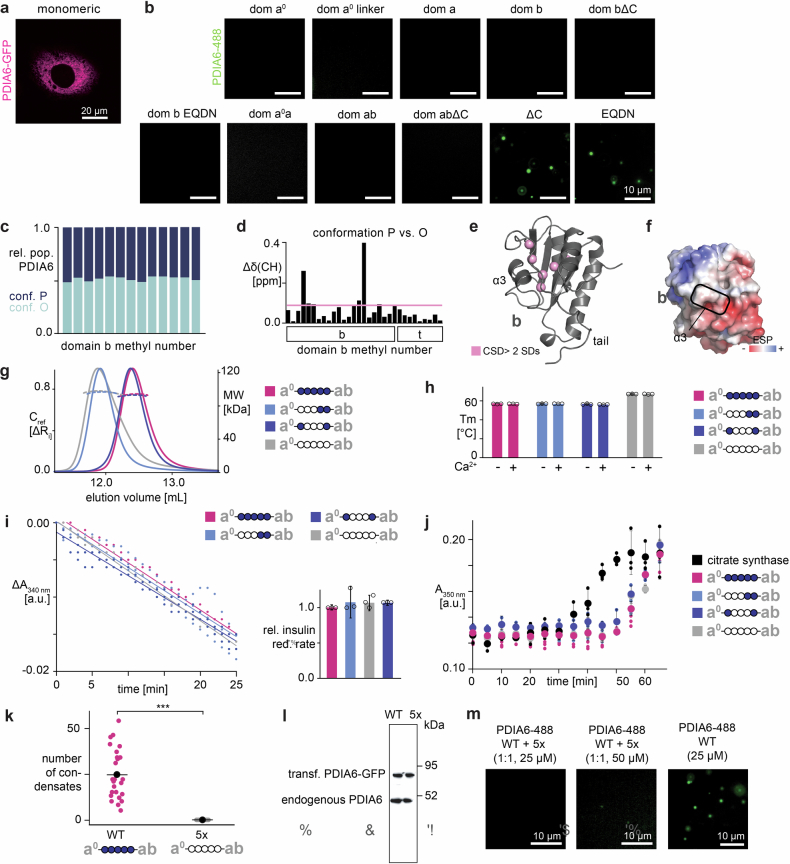
Specificity of PDIA6 phase separation in cells and in vitro*.* **a**, Live-cell imaging of HeLa cells transfected with a monomeric mutant^25^ of PDIA6–GFP. Scale bar, 20 μm. **b**, In vitro phase separation of Dylight 488–PDIA6 subconstructs. The EQDN mutant has been described before^25^ to not bind Ca^2+^ to the C-terminal tail (binding site III). Scale bar, 10 μm **c**, Residue-specific populations of the conformation P (dark blue) and O (light blue). Each bar corresponds to one methyl group in domain b. **d**, Combined methyl chemical shift differences between conformations P and O in domain b. The significance threshold of 2 SDs is indicated by a pink line. **e**, Methyl groups with substantial chemical shift difference between conformations P and O marked as pink spheres on the structure of domain b. Residues located around helix α3 show largest chemical shift differences between the two conformations. **f**, Electrostatic surface potential (ESP) of domain b. Helix α3 forms part of a negatively charged patch. **g**, SEC elution profiles (solid lines, left axis) and MALS apparent molecular mass (dotted lines, right axis) of PDIA6 WT (pink), (R142Q, K150N, R153Q) (light blue), (K150N, R153Q, K159N) (dark blue) and 5x linker mutant (grey). All proteins eluted as a dimer. **h**, Melting temperature of PDIA6 WT and linker mutant constructs in SEC buffer and in presence of 10 mM of Ca^2+^ determined by DSF. Mean and standard deviation (*n* = 3 technical triplicates). Colour code as in **g**. **i**, Insulin reduction by PDIA6 WT and linker mutants. Dots represent the average and the line the fit of three independent measurements (left). Insulin reduction rate of the linker mutants relative to the WT protein (right). Colour code as in **g**. Mean and standard deviation (*n* = 3 technical triplicates). **j**, Chaperone activity of PDIA6 WT and linker mutants assessed by the aggregation of citrate synthase as a model substrate. Colour code as in **g**. Mean and standard deviation (*n* = 3 technical triplicates). **k**, Quantification of condensates per cell of PDIA6 WT (same data as in Fig. 1e unstressed (us), pink) and PDIA6 5x linker mutant (grey) transfected cells (***P = 1.14·10^−7^, two-sided *t*-test), (*n* = 17 cells per condition pooled across three independent experiments). **l**, Protein expression levels of endogenous PDIA6 and transfected PDIA6–GFP in HeLa cells. Unprocessed blots are available. **m**, In vitro phase separation of Dylight 488–PDIA6 WT + Dylight 488–PDIA6 5x linker mutant at 1:1 stoichiometry in presence of 10 mM Ca^2+^ at a final protein concentration of 25 μM (left) and 50 μM (middle) and 25 μM Dylight 488–PDIA6 WT for comparison (right). Scale bar, 10 μm. Source data

Next, we set out to determine the molecular mechanism underlying condensate formation. Biological phase separation relies on multivalent interactions^15^ and therefore, we expected at least two intermolecular interactions to be involved. We suspected that dimerization of domain a^0^ is one of these interactions and indeed, a monomeric construct of PDIA6 (ref. ^25^) did not form condensates (Extended Data Fig. 7a). In search for the second interaction, we first tested droplet formation of the individual domain subconstructs but none of these formed droplets in solution (Extended Data Fig. 7b). As mentioned earlier, we also probed a potential role of the disordered C-terminal tail, but neither neutralization of its positive charges (EQDN) nor its entire truncation (ΔC) abrogated the formation of condensates (Extended Data Fig. 6h,i).

**Fig. 3:**
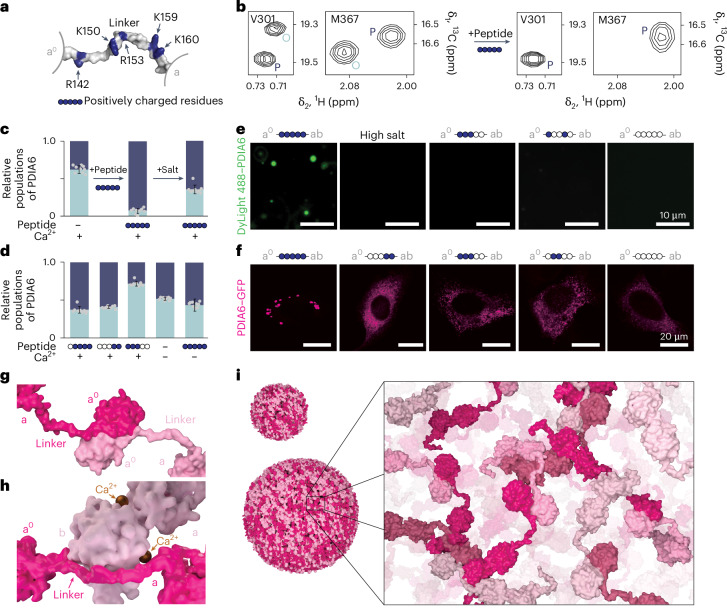
Mechanism of PDIA6 liquid–liquid phase separation condensate formation. **a**, Composition of the linker connecting domains a^0^ and a. **b**, Sections of a two-dimensional [^13^C,^1^H]-HMQC spectrum of PDIA6 WT in the presence of 10 mM Ca^2+^ (left) and with the addition of 10 eq. linker peptide (right) without crowding agent. The two peak sets of conformations P and O are shown on the example of residues V301 and M367. **c**, Relative populations of PDIA6 conformations P (dark blue) and O (light blue), in the presence of 10 mM Ca^2+^ and 10 eq. linker peptide (blue spheres) or following the addition of 1 M KCl. **d**, Relative populations of PDIA6 conformations P (dark blue) and O (light blue), as indicated. In **c** and **d**, data are the mean ± s.d. of *n* = 9 non-overlapping methyl group residues in domain b. NMR spectra are presented in Supplementary Fig. 1. **e**, In vitro phase separation of DyLight 488–PDIA6 constructs, as indicated, in the presence of crowding agent and 10 mM Ca^2+^. **f**, Live-cell images of HeLa cells transfected with PDIA6–GFP WT or linker mutants. In **c**–**f**, blue spheres represent positively charged residues in the peptide linker, while white spheres represent neutralizing mutations. **g**,**h**, Model of the domain a^0^a^0^ dimerization interface (**g**) as well as the intermolecular interaction between the linker and domain b of two PDIA6 dimers (**h**). One dimer of PDIA6 is coloured in dark pink, the other in light pink. **i**, Model of PDIA6 droplet formation (details in text). Source data

We then hypothesized that the highly flexible and charged linker connecting domains a^0^ and a (Fig. 3a) might drive condensate formation by interacting with another PDIA6 dimer. To resolve this interaction, we employed NMR spectroscopy on the disperse protein, that is, in the absence of crowding agent. We suspected this interaction to be rather weak and therefore added a 21-residue peptide corresponding to the a^0^–a linker (GGRSGGYSSGKQGRSDSSSKK) to methyl-labelled full-length PDIA6 to saturate any potential binding sites. The addition of the peptide indeed led to drastic spectral changes in domain b. In the absence of the peptide, the domain equally populated two conformations in slow exchange (two peaks per residue for all residues in domain b; 57:43 ± 8%). Following titration of the linker peptide, this equilibrium strongly shifted towards one conformation (93:7 ± 3%), evidencing a transient interaction between the linker and domain b (Fig. 3b,c and Extended Data Fig. 7c–e). High salt concentration as well as charge-neutralizing mutations in the linker peptide impaired the interaction, validating its electrostatic character (Fig. 3c,d and Extended Data Fig. 7f). Strikingly, the interaction was Ca^2+^-dependent, in full agreement with the physiological observation of phase separation (Fig. 1).

The data thus suggest a functional model for PDIA6 condensate formation, where the positively charged a^0^–a linker dynamically interacts with the negatively charged surface in domain b at homeostatic Ca^2+^ concentrations. We tested this functional model on the in vitro-reconstituted droplets of full-length PDIA6. In full agreement with the model, droplet formation was prevented by increased salt concentration or neutralization of the positively charged linker residues by mutagenesis (Fig. 3e). Importantly, the linker mutations did not affect the PDIA6 dimeric state or its disulfide isomerase and chaperone activities (Extended Data Fig. 7g–j). Encouraged by these findings, we then also probed the molecular model of condensate formation in cells. Neutralization of the positive charges in the linker should abolish PDIA6 condensate formation. Cells transfected with PDIA6 linker mutants indeed no longer formed the large condensates observed for PDIA6 WT. Removal of two charges from the linker led to a reduction in condensate size, and complete neutralization of all five charged residues (5× linker mutant) fully impeded condensate formation, acting as dominant negative (Fig. 3f and Extended Data Fig. 7k). The expression levels of endogenous PDIA6 and transfected PDIA6 5× linker mutant were approximately equal in our in vivo experiment, and at the same stoichiometry PDIA6 droplet formation was also abrogated in vitro (Extended Data Fig. 7l,m). The multivalency required for PDIA6 condensate formation thus results from (1) the high-affinity dimerization of domain a^0^ and (2) the transient Ca^2+^-dependent electrostatic interaction between the a^0^–a linker and domain b (Fig. 3g–i).

## PDIA6 recruits other chaperones into the condensates

**Fig. 4:**
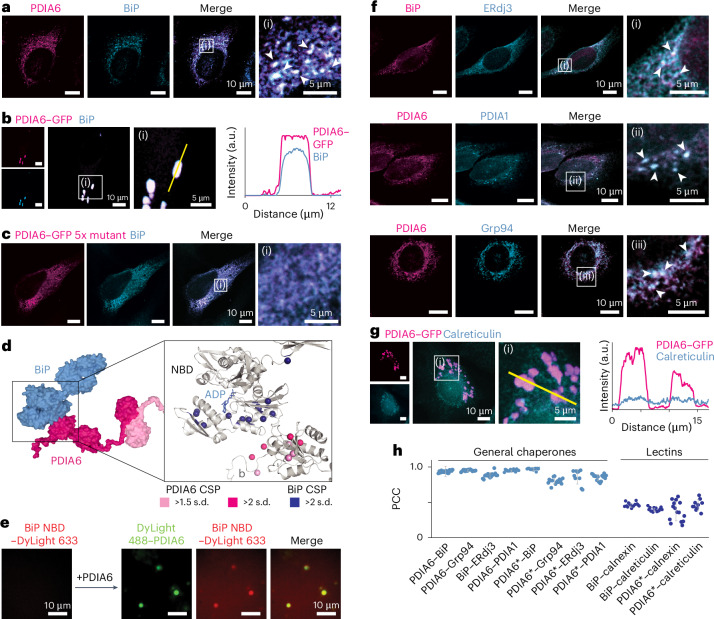
Chaperones involved in early client processing are selectively recruited into PDIA6 condensates. **a**, Co-localization of endogenous PDIA6 and BiP in fixed HeLa cells. Arrowheads exemplarily indicate the position of condensates. **b**, Co-localization of overexpressed PDIA6–GFP and endogenous BiP in fixed HeLa cells. Representative quantification from nine cells from three independent replicates. **c**, Co-localization of overexpressed PDIA6–GFP 5× linker mutant and endogenous BiP in fixed HeLa cells. PDIA6 was visualized using the eGFP signal. **d**, Docking model of PDIA6 and BiP, based on NMR data. Methyl groups with substantial chemical shift perturbation (CSP) upon interaction between PDIA6 and BiP are marked on the structures as coloured spheres. **e**, DyLight 633–BiP NBD in the presence of crowding agent and 10 mM Ca^2+^ (left), and co-localization with DyLight 488–PDIA6 (right) in vitro. **f**, Co-localization of endogenous BiP and ERdj3 (top), PDIA6 and PDIA1 (middle), and PDIA6 and Grp94 (blue) in fixed HeLa cells. Arrowheads exemplarily indicate the position of condensates. **g**, Co-localization of overexpressed PDIA6–GFP and endogenous calreticulin in fixed HeLa cells. In **b** and **g**, fluorescence intensities across the condensates were measured along the yellow line (left) and are shown as histograms (right). a.u., arbitrary units. **h**, Co-localization analysis of various ER chaperones and PDIA6 (or BiP). PCC for pixel intensity of proteins in HeLa cells as indicated. Transfected PDIA6–GFP WT is indicated with an asterisk; all other proteins are endogenous. Individual data points (PCC per cell; *n* = 10 for PDIA6*–ERdj3 and PDIA6*–calreticulin, 13 for PDIA6*–BiP and 15 for all other protein co-localizations), pooled from three independent experiments, and the s.d. are shown (details in Supplementary Table 2). Source data

**Extended Data Fig. 8:**
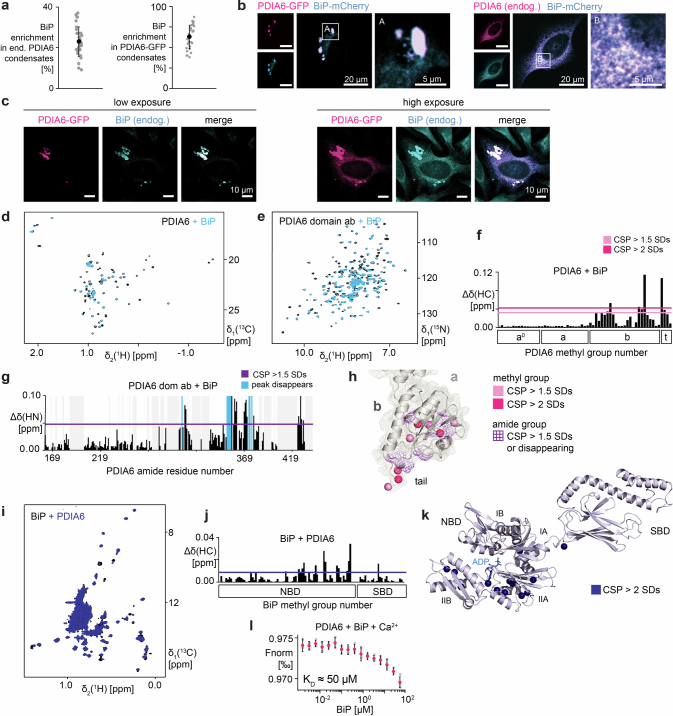
PDIA6 domain b recruits BiP into the condensates. **a**, Quantification of enrichment of BiP in endogenous PDIA6 condensates (left, *n* = 25 cells, pooled across three independent experiments, a total of 2,300 condensates were counted) and in transiently transfected PDIA6–GFP condensates (right, *n* = 31 cells, pooled across three independent experiments, a total of 720 condensates were counted). Mean and standard deviation. **b**, Co-localization of overexpressed PDIA6–GFP (pink) and overexpressed BiP–mCherry (blue) in live HeLa cells (left) and of endogenous PDIA6 (pink) and overexpressed BiP–mCherry (blue) in fixed HeLa cells (right). PDIA6–GFP was visualized by imaging eGFP, BiP–mCherry by imaging mCherry. Scale bar, 20 μm, insert 5 μm. **c**, Co-localization of overexpressed PDIA6–GFP (pink) and endogenous BiP (blue) in fixed HeLa cells at low (left) and high (right) exposure. Scale bar, 10 μm. **d**, Overlay of 2D [^13^C,^1^H]-HMQC spectra of 100 μM PDIA6 in absence (black) and in presence of 2 eq. BiP (blue). Full spectra shown in Supplementary Fig. 5a. **e**, Overlay of 2D [^15^N,^1^H]-HSQC spectra of 100 μM PDIA6 domain ab in absence (black) and in presence of 2 eq. BiP (blue). Full spectra shown in Supplementary Fig. 5b. **f**, Combined methyl chemical shift perturbation of PDIA6 upon titration of 2 eq. BiP. The magnitudes of 1.5 SD and 2 SDs are indicated by pink lines. **g**, Combined amide chemical shift perturbation of PDIA6 domain ab upon titration of BiP. The magnitude of 1.5 SD is indicated by a purple line, disappearing residues in blue. **h**, Methyl groups with substantial CSP upon interaction with BiP shown as pink spheres on the structure of domain b. Amide groups with substantial CSP are shown as purple surface. **i**, Overlay of 2D [^13^C,^1^H]-HMQC spectra of 100 μM methyl-labelled BiP in absence (black) and in presence of 2 eq. PDIA6 (blue). Full spectra shown in Supplementary Fig. 6. **j**, Combined methyl chemical shift perturbation of BiP upon titration of 2 eq. PDIA6. The magnitudes of 2 SDs is indicated by a blue line. **k**, Methyl groups with substantial CSP upon interaction with PDIA6 shown as blue spheres on the structure of the undocked ADP-bound BiP (modelled on PDB: 2kho). **l**, Microscale thermophoresis signal of Dylight 488–PDIA6 plotted against BiP concentration in presence of 1 mM Ca^2+^. Mean and standard deviation (*n* = 3, technical triplicates). Source data

To explore the biological role of the PDIA6 condensates, in particular their functional role in the ER chaperone network, we probed the localization of the central chaperone Hsp70 BiP^30^ relative to the condensates. BiP is one of the most abundant ER chaperones involved in protein folding and disaggregation and has been reported to interact with PDIA6 (refs. ^31–34^). Immunostaining of HeLa cells showed that endogenous BiP co-localized in the PDIA6 condensates, both at endogenous and overexpressed PDIA6 levels. Overexpression of BiP, in turn, did not affect the PDIA6 condensates (Fig. 4a,b, Extended Data Fig. 8a–c and Supplementary Table 2). We also tested whether BiP would form condensates on its own by staining endogenous BiP in cells transfected with PDIA6 5× linker mutant. As shown above, in these cells PDIA6 does not form condensates but is homogenously dispersed throughout the entire ER. Under this condition, BiP did not form condensates, thus establishing PDIA6 as the scaffold of the condensates to which BiP is recruited (Fig. 4c).

Next, we employed methyl and amide NMR experiments to determine the interaction sites between PDIA6 and BiP by chemical shift mapping. PDIA6 interacts via its domain b with the nucleotide binding domain (NBD) of BiP (Fig. 4d and Extended Data Fig. 8d–k). The affinity of *K*_D_ ≈ 50 μM (Extended Data Fig. 8l) is in the same range as known BiP–co-chaperone interactions^35,36^ and fully in line with a dynamic chaperone network. Importantly, the BiP binding site on domain b does not overlap with the interface required for condensate formation such that condensate formation and BiP recruitment by PDIA6 is simultaneously possible, as observed in cells. In full accordance with this model, the isolated BiP NBD co-localized in PDIA6 droplets in vitro, although it did not undergo phase separation by itself (Fig. 4e). Together, these experiments clearly establish that PDIA6 forms the scaffold of the condensates into which it recruits BiP as a secondary protein.

**Extended Data Fig. 9:**
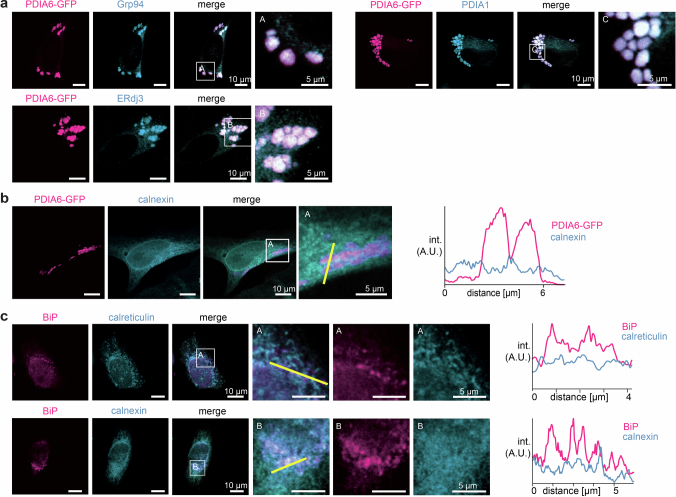
Composition of PDIA6 chaperone condensates. **a**, Co-localization of overexpressed PDIA6–GFP (pink) and endogenous Grp94 (blue) (top), overexpressed PDIA6–GFP (pink) and endogenous ERdj3 (blue) (bottom) and overexpressed PDIA6–GFP (pink) and endogenous PDIA1 (blue) (right) in fixed HeLa cells. PDIA6 was visualized using the eGFP signal. Scale bar, 10 μm, insert 5 μm. **b**, Co-localization of overexpressed PDIA6–GFP (pink) and endogenous calnexin (blue) in fixed HeLa cells. PDIA6 was visualized using the eGFP signal. Scale bar, 10 μm, insert 5 μm. Fluorescence intensities across the condensates were measured along the yellow line and are shown as histograms (right). Representative quantification from 9 cells from three independent replicates. **c**, Co-localization of endogenous BiP (pink) and endogenous calreticulin (blue) (top) or calnexin (blue) (bottom) in fixed HeLa cells. Scale bar, 10 μm, insert 5 μm. Fluorescence intensities across the condensates were measured along the yellow line and are shown as histograms (right).

Next, we investigated whether further ER chaperones were also concentrated into the PDIA6 condensates. We chose to examine the BiP co-chaperone ERdj3, an ER-resident member of the J-domain protein family, because it plays a central role in mediating client-protein transfer to BiP^37^. Strikingly, ERdj3 co-localized with the PDIA6–BiP condensates, both at endogenous protein levels and in PDIA6-transfected cells. Next, we probed the localizations of PDIA1 (the major PDI for numerous ER client proteins) and Grp94 (the ER-localized Hsp90), which cooperate with BiP in client-protein processing^34,38^. Both chaperones enriched in the PDIA6 condensates at endogenous protein levels and in PDIA6-transfected cells (Fig. 4f, Extended Data Fig. 9a and Supplementary Table 2). In contrast to this, the lectin chaperones calreticulin and calnexin (responsible for the glycosylation of client proteins before their export from the ER)^39^ did not enrich in the PDIA6 condensates (Fig. 4g, Extended Data Fig. 9b,c and Supplementary Table 2). From these observations, it emerges that the essential components of the early folding machinery—Hsp70, Hsp90, J-domain proteins and PDIs—specifically concentrate in the condensates, whereas the lectin chaperones involved in later-stage client maturation—calnexin and calreticulin—are not enriched (Fig. 4h).

## Chaperone condensates function as folding factories

The co-localization of multiple key chaperones in the condensates strongly suggests a fundamental biological function in client-protein folding. Proinsulin, the precursor of insulin, is known to be folded in the ER through the assistance of various chaperones including BiP, Erdj3, PDIA6 and PDIA1 (refs. ^40,41^) before being exported to the Golgi for further processing and subsequent secretion in the form of insulin^42^. This makes it an ideal model client to study the potential role of the condensates as folding compartments.

**Fig. 5:**
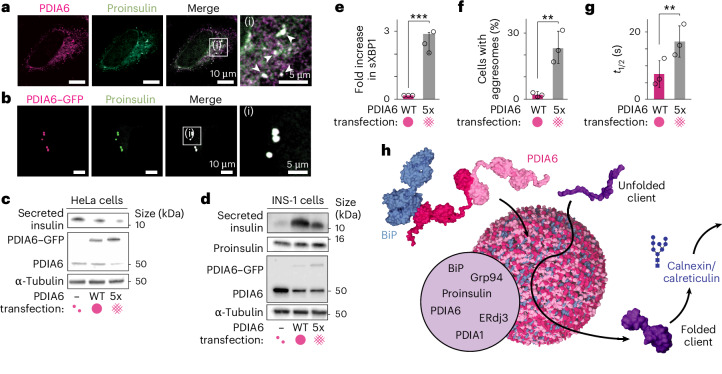
Chaperone condensates function as folding factories. **a**, Co-localization of endogenous PDIA6 and overexpressed proinsulin–myc in fixed HeLa cells. Arrowheads exemplarily indicate the position of condensates. **b**, Co-localization of overexpressed PDIA6–GFP and proinsulin–myc in fixed HeLa cells. **c**, Secretion levels of insulin (medium), and expression levels of PDIA6 and α-tubulin (lysate) in HeLa cells transfected with proinsulin, proinsulin + PDIA6–GFP WT or proinsulin + PDIA6–GFP 5× linker mutant. Proinsulin was not detectable in the lysate. **d**, Secretion levels of insulin (medium) as well as expression levels of PDIA6, proinsulin and α-tubulin (lysate) in INS-1 cells transfected with mock, PDIA6–GFP WT or PDIA6–GFP 5× linker mutant. In **c** and **d**, representative blots from three independent replicates. **e**, Fold increase in spliced XBP1 (sXBP1) in HeLa cells transfected with PDIA6 WT or 5× linker mutant relative to untransfected cells, determined using quantitative PCR with reverse transcription (*P* = 0.00092). **f**, Flow cytometry-based cell aggresome analysis of HeLa cells transfected with PDIA6–GFP WT or PDIA6–GFP 5× linker mutant, detected by PROTEOSTAT aggresome dye (*P* = 0.007). Gating strategy summarized in Supplementary Fig. 2. **g**, Half recovery time of mApple–Sec61 in HeLa cells co-transfected with PDIA6–GFP WT or PDIA6–GFP 5× linker mutant. Data from three independent experiments were fitted individually and averaged (*P* = 0.003). In **e**–**g**, data are the mean ± s.d.; *n* = 3 independent biological experiments per condition; two-sided Student’s *t*-test. **h**, Model of PDIA6 chaperones condensates functioning as folding factories (details in text). **P < 0.01, ***P < 0.001. Unprocessed blots are available. Source data

**Extended Data Fig. 10:**
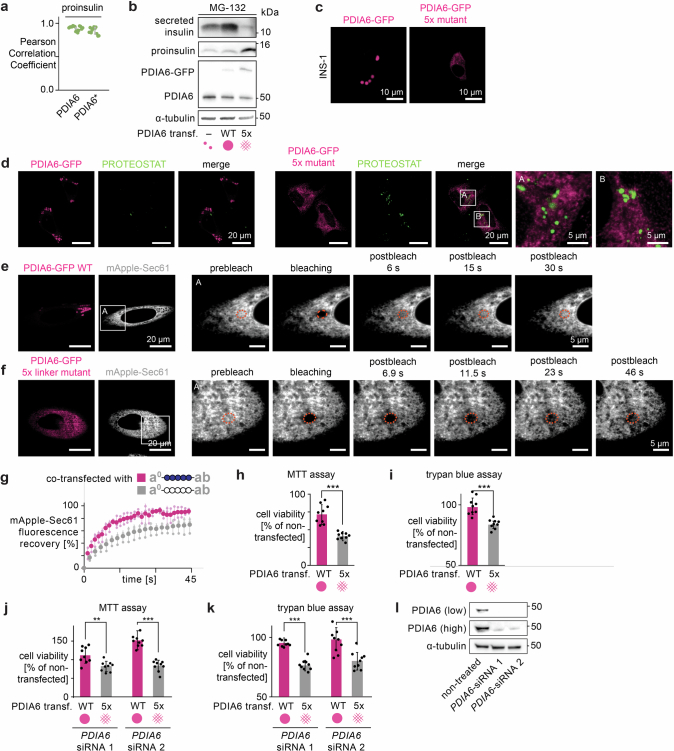
PDIA6-scaffolded chaperone condensates reduce ER protein aggregation. **a**, Co-localization analysis by Pearson Correlation Coefficient (PCC) for pixel intensity of proinsulin and endogenous and overexpressed (*) PDIA6 in HeLa cells. Individual data points (PCC per cell) (*n* = 15/11 cells for PDIA6-proinsulin and PDIA6*-proinsulin respectively) pooled across three independent experiments and standard deviation (for details see Supplementary Table 2). **b**, Secretion levels of insulin (medium) and expression levels of proinsulin, PDIA6 and α-tubulin (lysate) in proinsulin, proinsulin + PDIA6–GFP WT or proinsulin + PDIA6–GFP 5x linker mutant transfected HeLa cells after 18 h treatment with the proteasome inhibitor MG-132. **c**, Live-cell imaging of INS-1 cells transfected with PDIA6–GFP WT (left) or PDIA6–GFP 5x linker mutant (right). PDIA6–GFP was visualized by imaging eGFP. Scale bar, 10 μm. **d**, Aggresomes detected by PROTEOSTAT dye (green) in fixed HeLa cells transfected with PDIA6–GFP WT (left) or PDIA6–GFP 5x linker mutant (right) (pink). Scale bar, 20 μm, insert 5 μm. **e**,**f**, FRAP experiments of mApple–Sec61 (grey) in HeLa cells, co-transfected with PDIA6–GFP WT (**e**) or PDIA6–GFP 5x linker mutant (**f**) (pink). Due to strong bleaching of the mApple, contrast and brightness were adjusted for each image individually. Scale bar, 20 μm, insets 5 μm. **g**, Fluorescence recovery curve of photobleached mApple–Sec61 in PDIA6–GFP WT (pink) or PDIA6–GFP 5x linker mutant (grey) co-transfected HeLa cells. Average and standard deviation of experimental triplicates. Bleached areas did not overlap with condensates. **h**,**i**, Cell viability of PDIA6–GFP WT (pink) and PDIA6–GFP 5x linker mutant (grey) transfected cells relative to mock-transfected cells by MTT. (***P = 0.0003, two-sided *t*-test) (*n* = 9, triplicates of three independent biological experiments; **h**) and trypan blue assay (***P = 0.0003, two-sided *t*-test) (*n* = 9, parallel triplicates from three independent biological experiments; **i**). For the trypan blue assay a total of 12,760 (PDIA6–GFP WT), 8,519 (PDIA6–GFP 5x) and 16,150 (mock-transfected) cells were counted (**i**). Mean and standard deviation. **j**,**k**, Cell viability of *PDIA6*-knockdown cells, of PDIA6–GFP WT (pink) and PDIA6–GFP 5x linker mutant (grey) transfected cells relative to mock-transfected cells by MTT (*PDIA6-*siRNA1: **P = 0.003, *PDIA6-*siRNA2: ***P = 0.00008, two-sided t-test)(n = 9, triplicates of three independent biological experiments; **j**) and trypan blue assay (*PDIA6-*siRNA1: ** *p* = 1.64·10^−8^, *PDIA6-*siRNA2: *** *p* = 0.0004, two-sided *t*-test) (*n* = 9, triplicates of from three independent biological experiments; **k**). For the trypan blue assay a total of 90,350 (PDIA6–GFP WT, siRNA1), 76,070 (PDIA6–GFP 5x, siRNA1), 75,040 (mock-transfected, siRNA1), 54,850 (PDIA6–GFP WT, siRNA2), 66,210 (PDIA6–GFP 5x, siRNA2) and 41,530 (mock-transfected, siRNA2) *PDIA6*-knockdown cells were counted (**k**). Mean and standard deviation. **l**, Western blot showing expression levels of PDIA6 and α-tubulin and in non-treated and *PDIA6*-knockdown (siRNA1 and siRNA2) HeLa cells. Unprocessed blots are available. Source data

To assess the spatial localization of proinsulin within the ER, cells were transiently transfected with a proinsulin construct including a C-terminal myc tag. This allowed visualization of ER-localized proinsulin through staining with antibody to myc and revealed that proinsulin was highly enriched in endogenous PDIA6 condensates (Pearson’s correlation coefficient, PCC = 0.93 ± 0.1), which strongly points towards a role of the condensates in client-protein folding. Similarly, proinsulin co-localized in PDIA6–GFP condensates (PCC = 0.88 ± 0.06; Fig. 5a,b, Extended Data Fig. 10a and Supplementary Table 2).

To evaluate whether proinsulin is actively processed within the PDIA6 condensates, we assessed their effect on insulin secretion. If the PDIA6 condensates served as functional ER folding compartments, their absence should lead to a decrease in insulin secretion. We co-transfected cells with proinsulin and either PDIA6 WT (condensates) or the PDIA6 5× linker mutant (no condensates), and determined the amount of insulin secretion. Strikingly, cells transfected with PDIA6 5× linker mutant secreted less insulin than PDIA6 WT-transfected cells, supporting our hypothesis of a functional role of the PDIA6 condensates in client maturation (Fig. 5c). Notably, intracellular proinsulin was not detectable in these experiments, presumably due to its proteolytic degradation. We thus treated the cells with the proteasome inhibitor MG-132, which led to detectable proinsulin accumulation (Extended Data Fig. 10b). Thereby, proinsulin levels were notably higher in cells expressing PDIA6 5× linker mutant (no condensates) compared with PDIA6 WT (condensates), which further demonstrates that the absence of condensates impairs proinsulin folding. To corroborate this, we also used the rat insulinoma cell line INS-1, which exhibits an inherently high glucose-stimulated insulin secretion, overcoming the requirement of proinsulin co-transfection. Comparison of the secreted insulin with the intracellular proinsulin ratio of PDIA6 WT- and PDIA6 5× linker mutant-transfected INS-1 cells led to the same conclusion as before, namely that the absence of the chaperone condensates severely disrupts insulin processing (Fig. 5d and Extended Data Fig. 10c). As shown earlier, the enzymatic activity of PDIA6 is fully intact in the 5× linker mutant such that the disruption of proinsulin folding must result from the disruption of the condensate architecture. Together, these experiments provide clear evidence that the PDIA6 condensates as such play a fundamental role in insulin folding that goes beyond classical chaperone functions.

Finally, we wanted to study the effect of the PDIA6–chaperone condensates on general ER client-protein turnover and proteostasis. To this end, we assessed the effect of chaperone condensates on three parameters that report on ER proteostasis: (1) XBP1 splicing, a well-established readout that directly reports on the accumulation of misfolded proteins in the ER lumen^43,44^, (2) protein aggregation using the PROTEOSTAT aggresome assay and (3) fluidity of the ER membrane, which has been reported to stiffen on accumulation of misfolded proteins, measured via the recovery dynamics of mApple–Sec61 in FRAP experiments. We transfected cells with PDIA6 WT or PDIA6 5× linker mutant and quantified the above parameters. Strikingly, compared with PDIA6 WT-expressing cells, the absence of PDIA6 condensates (5× linker mutant) led to a pronounced increase in XBP1 splicing (Fig. 5e) and greater protein aggregation (Fig. 5f and Extended Data Fig. 10d). The fluorescence recovery of mApple–Sec61 was significantly slower in cells without PDIA6 condensates (*t*_1/2_ = 17 ± 5 s) than cells with PDIA6 condensates (*t*_1/2_ = 8 ± 4 s; Fig. 5g and Extended Data Fig. 10e–g), showing a stiffening of the ER morphology in the absence of the chaperone condensates. Together, these results lend strong support for our model that the PDIA6 condensates play an active role in preventing protein aggregation and enhancing protein folding robustness. This is further reflected in a lower cell viability in the absence of condensates (Extended Data Fig. 10h–l), indicating that condensates avert cell death by preventing the accumulation of misfolded proteins. PDIA6 condensates thus serve to subcompartmentalize the ER into functional ‘folding factories’.

In summary, our experiments reveal along multiple independent lines that crucial ER chaperones do not only cooperate in a functional network but form a self-organized supramolecular structure within the ER that guides client folding (Fig. 5h). The chaperone condensates seem to act in the early stages of client-protein folding and it is thus imaginable that client proteins are imported directly into the condensates following their translocation into the ER. The physiological benefits of subcompartmentalizing the ER by arranging chaperones into folding condensates indeed seem to be numerous: the elevated local concentrations of chaperones and clients increases both specificity and folding efficiency, thus reducing protein misfolding and aggregation. The simultaneous decrease in chaperone concentration outside the condensates may prevent non-specific interactions with folded proteins in the ER lumen, thus enabling a multistep processing pipeline for clients. Furthermore, the dynamic regulation of the chaperone condensates via the Ca^2+^ concentration might help to overcome an increasing load of misfolded proteins in the ER lumen during acute ER-stress situations by accelerating protein turnover at the cost of specificity. Condensate properties have recently been reported for the cytosolic chaperones Hsp70 and J-domain proteins, to specifically prevent aggregation of amyloid-forming proteins^13,45^. From these and our observations, the pattern seems to emerge that subcompartmentalization by chaperone condensates may be a general theme to structure cellular compartments. The chaperone superstructures in the ER not only provide a fascinating layer of functionality to enable protein biogenesis but might also result in novel therapeutical approaches to target protein misfolding diseases.

## Methods

### Protein expression and purification

#### Cloning, expression and purification of human PDIA6

Human PDIA6 lacking its ER signal sequence (residues 19–440), domain ab (residues 161–440) and domain b (residues 276–440), with an additional N-terminal His_6_ tag including a tobacco etch virus (TEV) cleavage sequence and domain a (residues 161–272) with an additional N-terminal His_6_ tag, followed by a SUMO tag were synthesised by GenScript. The genes were inserted using NcoI and XhoI restriction enzymes into a pET28a expression vector (Novagen). BL21-(DE3)-Lemo cells (New England Biolabs) were transformed and cultured at 37 °C in Luria–Bertani (LB) medium containing 30 mg ml^−1^ kanamycin. Expression was induced at an optical density at 600 nm (OD_600_) of 0.6 by adding 0.5 mM isopropyl-β-d-thiogalactopyranoside (IPTG) and incubating at 25 °C for 12 h. The cells were harvested by centrifugation at 4,000*g* for 20 min. The pellet was resuspended in 35 ml lysis buffer per litre of culture (25 mM HEPES (pH 7.5), 300 mM NaCl, 10 mM MgCl_2_, 5 mM ATP (Sigma-Aldrich), 0.02 mg ml^−1^ ribonuclease RNase A (Roche), 0.01 mg ml^−1^ deoxyribonuclease DNase I (AppliChem) and 0.2 mg ml^−1^ phenylmethylsulfonyl fluoride). Cell lysis was performed using a microfluidizer (Microfluidics; three cycles at 4 °C). The soluble bacterial lysate was separated from cell debris and other components by centrifugation at 14,000*g* for 60 min and loaded onto a Ni-NTA column (Cytiva) equilibrated in buffer A (25 mM HEPES (pH 7.5), 300 mM NaCl and 10 mM imidazole). PDIA6 was eluted using a 60% step gradient of buffer B (25 mM HEPES (pH 7.5), 300 mM NaCl and 500 mM imidazole) and dialysed overnight against buffer (25 mM HEPES (pH 7.5) and 300 mM NaCl) to remove the imidazole. The PDIA6 was denatured with 6 M urea and loaded onto a Ni-NTA column (Cytiva) equilibrated in buffer A + 6 M urea to further separate the protein from nucleotide and protein contamination. No changes in the protein conformation of refolded and not refolded PDIA6 were observed in two-dimensional-HMQC spectra and SEC-MALS analyses. PDIA6 was eluted at a 300 mM imidazole concentration in buffer containing 6 M urea. PDIA6 refolding was achieved by overnight dialysis against buffer (25 mM HEPES (pH 7.5) and 300 mM NaCl). After refolding, the His tag was cleaved by overnight incubation with 1 mg TEV protease or Ulp1 SUMO protease per 50 mg of PDIA6 in cleavage buffer (25 mM HEPES (pH 7.5), 300 mM NaCl, 1 mM dithiothreitol and 0.5 mM EDTA) at 4 °C. The his–TEV–domain b and his–TEV–domain ab constructs were not cleaved. His–TEV–domain a^0^ and his–TEV–domain a used for amide backbone assignment were not cleaved. Cleaved PDIA6 constructs were separated from the tag, TEV or Ulp1 and uncleaved PDIA6 by a Ni-NTA column equilibrated in buffer A. Finally, PDIA6 was concentrated by ultrafiltration and injected into size-exclusion chromatography columns (Superdex-200 16/600 PG for constructs >30 kDa or Superdex-75 16/600 PG for constructs <30 kDa; Cytiva) to further purify the proteins and adjust the protein to its final buffer (25 mM HEPES (pH 7.5), 150 mM KCl and 10 mM MgCl_2_). The PDIA6 was concentrated by ultrafiltration and stored at −20 °C until use. The final yield of purified protein was 25 mg per litre of LB medium. To obtain fully reduced PDIA6, the protein was incubated with 10 mM tris(2-carboxyethyl)phosphine (TCEP) at room temperature for 1 h, buffer exchanged (3×) to SEC buffer (25 mM HEPES (pH 7.5), 150 mM KCl and 10 mM MgCl_2_) and immediately frozen. Before performing the calcium-binding experiments, PDIA6 was incubated with 20 mM EDTA for 1 h at room temperature and dialysed overnight against SEC buffer at 4 °C.

#### Cloning, expression and purification of human BiP

Human BiP lacking its ER signal sequence (residues 19–654) with an N-terminal His_6_ tag including a TEV cleavage sequence was synthesized by GenScript. The gene was inserted through NcoI and XhoI into a pET28a expression vector (Novagen). BL21-(DE3)-Lemo cells (New England Biolabs) were transformed and cultured at 37 °C in LB medium. BiP expression was induced at an OD_600_ of 0.6 by adding 1 mM IPTG, and expression was continued at 25 °C for 12 h. The cells were harvested by centrifugation at 5,000*g* for 20 min. The pellet was resuspended in 20 ml lysis buffer per litre of culture (25 mM HEPES (pH 7.5), 150 mM NaCl, 10 mM MgCl_2_, 0.02 mg ml^−1^ ribonuclease RNase A (Roche), 0.01 mg ml^−1^ deoxyribonuclease DNase I (AppliChem) and 0.2 mg ml^−1^ phenylmethylsulfonyl fluoride). Cell lysis was performed using a microfluidizer (Microfluidics; three cycles at 4 °C). The soluble bacterial lysate was separated from cell debris and other components by centrifugation at 14,000*g* for 60 min and loaded onto a Ni-NTA column (Cytiva) equilibrated in buffer A (25 mM HEPES (pH 7.5), 150 mM NaCl and 10 mM MgCl_2_). BiP was eluted using a 60% step gradient of buffer B (25 mM HEPES (pH 7.5), 150 mM NaCl, 10 mM MgCl_2_ and 1 M imidazole) and dialysed overnight against buffer (25 mM HEPES (pH 7.5), 300 mM NaCl and 10 mM MgCl_2_). The BiP was denatured with 6 M urea and loaded onto a Ni-NTA column equilibrated in buffer + 8 M urea. The BiP was eluted at a 500 mM imidazole concentration in buffer containing 8 M urea. BiP refolding was achieved by overnight dialysis against buffer (25 mM HEPES (pH 7.5), 300 mM NaCl and 10 mM MgCl_2_). After refolding, the His tag was cleaved by overnight incubation with 1 mg TEV per 50 mg BiP in cleavage buffer (25 mM HEPES (pH 7.5), 300 mM NaCl, 10 mM MgCl_2_, 1 mM dithiothreitol and 0.5 mM EDTA) at 4 °C. BiP was separated from the tag, TEV and uncleaved BiP via a Ni-NTA column (Cytiva) equilibrated in buffer A. The BiP was then applied to an anion-exchange column (QHP, Cytiva) equilibrated in buffer QA (25 mM Tris (pH 8.5)) and eluted using a gradient of buffer QB (25 mM Tris (pH 8.5) and 1 mM KCl) at a concentration of 250 mM KCl. Finally, the BiP was concentrated by ultrafiltration and subjected to size-exclusion chromatography (Superdex-200 16/600 PG, Cytiva) to further purify the proteins and adjust to its SEC buffer. Finally, the BiP was concentrated by ultrafiltration and stored at −20 °C until use. The final yield of purified protein was 15–20 mg WT BiP per litre of LB medium.

#### Methyl labelling of human PDIA6 and BiP

Methyl-labelled PDIA6 proteins were obtained by growing the expression cells in M9 minimal media prepared with 99.85% D_2_O (Sigma-Aldrich) containing ^15^NH_4_Cl (1 g l^−1^; Sigma-Aldrich) and d-glucose-d_7_ (2 g l^−1^; Sigma-Aldrich). At an OD_600_ of 0.8, a solution containing the labelled precursors was added as follows. For [U-^2^H, ^15^N, ^12^C], Met-[^13^CH_3_]^ε^, Val-[^13^CH_3_]^γ2^ and Leu-[^13^CH_3_]^δ2^ labelling of PDIA6 methionine leucine valine, 240 mg 2-hydroxy-2-[^13^C]methyl-3-oxo-4,4,4-tri-[^2^H]-butanoate (pro-S acetolactate-^13^C, NMR-Bio) and 100 mg of [^13^C]^ε^-L-methionine (Sigma-Aldrich). For [U-^2^H, ^15^N, ^12^C], Met-[^13^CH_3_]^ε^ and Val-[^13^CH_3_]^γ2^ labelling of PDIA6 methionine valine, 240 mg 2-hydroxy-2-[^13^C]methyl-3-oxo-4,4,4-tri-[^2^H]-butanoate (pro-S acetolactate-^13^C, NMR-Bio), 30 mg of l-leucine-d_10_ and 100 mg of [^13^C]^ε^-l-methionine (Sigma-Aldrich). For [U-^2^H, ^15^N, ^12^C], Met-[^13^CH_3_]^ε^, Val-[^13^CH_3_/^12^C^2^H_3_]^cγ1/cγ2^ and Ile-[^13^CH_3_]^δ1^ labelling of BiP isoleucine methionine valine, 100 mg 2-keto-3-(methyl-d3)-butyric acid-4-^13^C,3-d sodium salt, 30 mg l-leucine-d_10_, 80 mg α-ketobutyric acid methyl ^13^C (99%) 3,3-D2 (98%) and 100 mg [^13^C]^ε^-l-methionine (Sigma-Aldrich). One hour after the addition of the precursors, protein expression was induced by adding 0.5–1 mM IPTG and incubating at 25 °C for 12 h. Methyl-labelled proteins were purified following the protocols described earlier.

#### Mutagenesis, expression and purification of the PDIA6 and BiP mutants

The QuikChange II mutagenesis protocol (Stratagene) was used to introduce the PDIA6 mutations M182L; M331L; M359L; M367L; L61M; V75I; V124I; L139M; L165M; L196M; I234V; L320M; L345M; L349M; L385M; I406V; V407I; L419M; V421I; I425V; L427M; L432M; L435M; L440M; K161 to stop (domain a^0^); L139STOP (domain a^0^ Δlinker); P274 to stop (domain a^0^a, domain a); S428 to stop (domain b ΔC(13)); 420STOP (ΔC(20); domain b ΔC(20); domain ab ΔC(20)); E422Q, D423N, D424N, D426N, D429N, E431Q, D433N and D434N (EQDN, dom b EQDN); R142Q, K150N and R153Q; K150N, R153Q and K159N; R142Q, K150N, R153Q, K159N and K160N (5× linker mutant) and the BiP mutation G407 to stop (NBD). All PCR primers were obtained from Microsynth. The plasmids were purified using the Zyppy plasmid mini prep kit (Zymo Research) and mutations were confirmed by sequencing by Microsynth AG. The expression and purification of the mutant proteins was performed as described for the WT proteins. All linker mutants (R142Q, K150N and R153Q; K150N, R153Q and K159N; and R142Q, K150N, R153Q, K159N and K160N) behaved like the WT protein.

### NMR spectroscopy

NMR experiments were performed in 25 mM HEPES (pH 7.5), 150 mM KCl, 10 mM MgCl_2_ or 25 mM 2-morpholinoethanesulfonic acid (MES) (pH 6.5), 150 mM KCl and 10 mM MgCl_2_ at 37 °C. The experiments were recorded on Bruker Avance IIIHD 600 MHz, Avance III 700 MHz, Avance Neo 800 MHz or Avance IIIHD 900 MHz spectrometers running Topspin 3.6–4.0 and equipped with a cryogenically cooled triple-resonance probe. The NMR data were processed with nmrPipe^46^ 2022.230.12.16 and ccpnmr^47^ 2.5.2. Reference experiments were recorded at concentrations between 25 μM and 100 μM. Secondary chemical shifts were calculated relative to random-coil values^48^. Depending on the conditions, 10 mM TCEP, 10 mM CaCl_2_, 5 mM ADP, 10 eq. linker peptide and 1 mM K_2_HPO_4_/KH_2_PO_4_ were used as indicated. To mimic ER homeostatic-like conditions, 10 mM TCEP or 15 mM glutathione (GSH) with 7:1 GSH:glutathione disulfide (GSSG) was used in the presence of 10 mM Ca^2+^. Samples behaved identically in the presence of 10 mM TCEP and 15 mM GSH with 7:1 GSH:GSSG. The samples were incubated before the experiment (about 1 h for TCEP, GSH and ADP; about 30 min for protein–protein interaction experiments, Ca^2+^ and Gd^3+^; and overnight for peptides). Data were analysed using Excel 16.97.

### NMR assignments

#### Assignment of PDIA6 Met[CH_3_]^ε^, Val[CH_3_]^γ2^ and Leu[CH_3_]^δ2^ methyl groups

PDIA6 Met[CH_3_]^ε^, Val[CH_3_]^γ2^ and Leu[CH_3_]^δ2^ assignment was obtained using a structure-based approach combining mutagenesis, specific valine labelling schemes^49^, subconstructs and three-dimensional ^13^C, ^13^C-resolved [^1^H, ^1^H]-NOESY experiments recorded at 37 °C in SEC buffer with 10 mM TCEP and 5% D_2_O. The following point mutations were used: M182L, M331L, M359L, M376L, L61M, V75I, V124I, V139M, L165M, L196M, I234V, L320M, L345M, L349M, L385M, I406V, V407I, L419M, V421I, I425V, L427M, L432M, L435M and L440M. The following subconstructs were used: his–domain a and his–domain ab. Specific valine labelling allowed distinction between leucine and valine residues. Each sample was recorded at 37 °C with an adjusted duration depending on the final concentration of each mutant (experimental time ranging from 120 to 240 min per sample). Three-dimensional ^13^C, ^13^C-resolved [^1^H, ^1^H]-NOESY experiments were recorded in 25 mM HEPES (pH 7.5), 150 mM KCl, 10 mM MgCl_2_, 10 mM TCEP and 5% D_2_O, with sample concentrations of 870 μM and a mixing time of 500 ms. Together, this allowed for the assignment of 62/66 Met[CH_3_]^ε^ and Leu[CH_3_]^δ2^ and Val[CH_3_]^γ2^ methyl groups (94%).

#### Amide backbone assignment

For the sequence-specific backbone resonance assignments, the following experiments were recorded. [*U*-^1^H, ^13^C, ^15^N]-PDIA6 domain a^0^: HNCA and HNCACB at 37 °C in 25 mM MES (pH 6.5), 150 mM KCl, 10 mM MgCl_2_, 10 mM TCEP and 5 % D_2_O. [*U*-^1^H, ^13^C, ^15^N]-PDIA6 his–domain a: 4D-APSY^50^ at 37 °C in 25 mM MES (pH 6.5), 150 mM KCl, 10 mM MgCl_2_, 10 mM TCEP and 5% D_2_O. [*U*-^2^H, ^13^C, ^15^N]-PDIA6 his–domain ab: HNCA, HNCACB, HNCO and HNcoCA at 37 °C in 25 mM MES (pH 6.5), 150 mM KCl, 10 mM MgCl_2_, 10 mM TCEP and 5 % D_2_O; and HNCA, HNCACB with deuterium decoupling and 3D ^15^N, ^15^N resolved [^1^H, ^1^H]-NOESY with a mixing time of 100 ms at 37 °C in 25 mM HEPES (pH 7.5), 150 mM KCl, 10 mM MgCl_2_, 10 mM TCEP and 5% D_2_O. [*U*-^1^H, ^13^C, ^15^N]-PDIA6 his–domain bΔC: HNCA, HNCACB, HNcoCACB and HNCO at 25 °C in 25 mM HEPES (pH 7.5), 150 mM KCl, 10 mM MgCl_2_, 10 mM GSSG and 5% D_2_O, and HNCACB, HNcaCO and 3D ^15^N, ^15^N resolved [^1^H, ^1^H]-NOESY with a mixing time of 80 ms at 25 °C in 25 mM HEPES (pH 7.5), 150 mM KCl, 10 mM MgCl_2_, 10 mM TCEP and 5 % D_2_O. All experiments were recorded at sample concentrations of 0.5–1.0 mM. The amide backbone assignments comprise 85% of domain a^0^, 92% of his–domain a, 79% of his–domain b and 54% of his–domain ab.

### Fluorescent labelling of purified recombinant PDIA6 and BiP NBD

All PDIA6 constructs were N-terminally tagged with DyLight 488 amine-reactive dyes (Thermo Scientific) at a concentration of 10 mg ml^−1^ in 25 mM MES (pH 6.5), 150 mM KCl and 10 mM MgCl_2_ in the dark for 1 h at 25 °C. Free dye was removed by overnight dialysis at pH 6.5 and 4 °C in the dark, and the labelled proteins were buffer exchanged (3×) to SEC buffer. BiP NBD was N-terminally tagged with DyLight 633 amine-reactive dyes (Thermo Scientific) following the same protocol. The protein concentration and extent of labelling were determined according to manufacturer’s protocol for all constructs and only proteins labelled with 0.75 and 1.5 moles dye per mole protein were used for experiments.

### SEC-MALS

SEC-MALS measurements of PDIA6 constructs were performed at 25 °C in SEC buffer using a S200 10/300 GL column (Agilent Technologies) on an Agilent 1260 high-performance liquid chromatography system. Proteins were eluted at a concentration of 20 μM. Elution was monitored by multiangle light scattering (Heleos II 8+; Wyatt Technology), differential refractive index (Optilab T-rEX; Wyatt Technology) and absorbance at 280 and 254 nm (1260 UV; Agilent Technologies). The column was equilibrated overnight in the running buffer to obtain stable baseline signals from the detectors before data collection. All system parameters were calibrated using an injection of 2 mg ml^−1^ BSA solution (ThermoPierce) and standard protocols in ASTRA 6. Molar mass and mass distributions were calculated using the ASTRA 6 software (Wyatt Technology).

### Differential scanning fluorimetry

Differential scanning fluorimetry data were acquired using a Prometheus NanoTemper NT.48 instrument (NanoTemper Technology) at protein concentrations of 100 μM in SEC buffer. The samples were scanned from 20 °C to 95 °C at a scan rate of 0.5 °C min^−1^. Protein unfolding was measured by detecting the temperature-dependent change in tryptophan fluorescence at emission wavelengths of 330 and 350 nm. Melting temperatures were determined by detecting the maximum of the first derivative of the fluorescence ratios (F350/F330).

### Microscale thermophoresis experiments

For the determination of the dissociation constants N-terminally tagged DyLight 488–PDIA6 at 20 nM was incubated with increasing amounts of BiP in SEC buffer in the presence of 5 mM ADP, 1 mM KH_2_PO_4_/K_2_HPO_4_, 0.05% Tween and 1 mM CaCl_2_ prepared by serial dilution. The samples were incubated for 30 min at 25 °C before being loaded into capillary tubes. The LED power was set to 100% and the microscale thermophoresis (MST) power to medium. The pre-MST period was 5 s, the MST-acquisition period was 30 s and the post-MST period was 5 s. Measurements were performed in triplicates on a Monolith NT.115 system (NanoTemper) and integrated at an interval of 2.5 s.

### Mass photometry

Mass photometry data were obtained using a Refeyn OneMP system in microscope coverslips prepared according to the manufacturer’s instructions with silicone gaskets. To lock the focus, 18 µl SEC buffer was used, followed by the addition of 2 µl protein sample at the indicated final concentrations. Movies were recorded using Refeyn Acquire^MP^ 2.5.1 and analysed with Refeyn Discover^MP^ 2.5.0.

### Crystallization, data collection and structure determination of PDIA6 domain bΔC

PDIA6 his–domain bΔC (P274–L427) in 25 mM HEPES (pH 7.5), 150 mM KCl, 5 mM MgCl_2_ and 10 mM TCEP was crystallized at 10 mg ml^−1^ concentration at room temperature in sitting-drop vapour diffusion experiments in a 1:1 ratio of protein to precipitant. Crystals appeared in 1.9 M (NH4)_2_SO_4_, 0.1 M HEPES (pH 7.0) and 1% dimethylsulfoxide after two days and grew to their final size within ten days. The crystals were cryopreserved by the addition of ethylene glycol to a final concentration of 20% (vol/vol) and flash-cooled in liquid nitrogen. Data were collected at the SLS beamline X06DA (Swiss Light Source, Paul Scherrer Institute, Switzerland) at 100 K, and integrated, indexed and scaled using the XDS software^51,52^. The crystal structure was determined by molecular replacement with Phaser^53^ 1.0 using PDB accession code 3uem (ref. ^54^). Automated model building^55^ was carried out with Arp/Warp^56^ 8.0, followed by Autobuild in PHENIX^57^ 1.17, and completed by manual model building with Coot^58^ 0.9.8 and structure refinement in PHENIX^59^ 1.17. Model quality was validated with Molprobity^60^ 4.4. Data collection and refinement statistics are in Supplementary Table 1. The atomic coordinates and structure factors have been deposited in the PDB under the accession code 8cpq.

### Insulin reduction assay

Disulfide reductase activity was assessed using the NADPH-based insulin reduction assay. Purified PDIA6 constructs (1 μM final concentration) were added to 1 mM reduced GSH, 800 μM NADPH, 0.16 units GSH reductase (Sigma-Aldrich) and 30 μM human insulin (Sigma-Aldrich) in SEC buffer and the absorbance of NADPH monitored at 340 nm using a Biotek Synergy H1 multi-mode microplate reader for 25 min at 25 °C.

### Chaperone activity assay

Chaperone activity was assessed using the citrate synthase aggregation assay. Citrate synthase (*Escherichia coli*; NZYTech) was dialysed against 40 mM HEPES (pH 7.5). Purified PDIA6 constructs (10 μM final concentration) were added to 1 μM citrate synthase in 40 mM HEPES (pH 7.5) buffer and incubated at 45 °C and 300 rpm. Protein aggregation was monitored at 350 nm using a Biotek Synergy H1 multi-mode microplate reader at the indicated time points.

### In vitro LLPS

PDIA6 liquid–liquid phase separation (LLPS) was examined at 25 μM in SEC buffer in the presence of 10% polyethylene glycol (PEG3350, mass fraction, freshly prepared immediately before the experiment) as a crowding agent in the absence/presence of 15 mM GSH with 7:1 GSH:GSSG or 10 mM TCEP, 10 mM CaCl_2_ and/or 20 mM EDTA. The PEG was added last by simultaneously swirling the sample and gently pipetting up and down to thoroughly mix the sample. Images were taken 30 min after the addition of PEG. No PDIA6 construct formed liquid droplets in the absence of crowding agent and at concentrations below 1 mM. Phase-separated PDIA6 droplets formed under ER-mimicking conditions (15 mM GSH with 7:1 GSH:GSSG + 1–10 mM CaCl_2_) as well as under strongly reducing conditions (10 mM TCEP) in the presence of 1–10 mM CaCl_2_. Several salt concentrations (50–350 mM) were tested. Unlabelled proteins were used to confirm that they undergo phase separation without fluorescent labelling. Glass-bottomed 96-well plates were coated with 1% Pluronic F-127 overnight and the wells were washed three times with SEC buffer. For fluorescence microscopy, 1% N-terminally tagged DyLight protein was added to the unlabelled protein. BiP NBD LLPS was examined at 20 μM in SEC buffer in the presence of 5 mM ADP and 10% PEG as a crowding agent in the absence or presence of 20 μM PDIA6. Samples including BiP NBD were prepared as described for PDIA6.

### In vitro fluorescence microscopy

A wide-field microscope (FEI MORE) with a Hamamatsu ORCA flash 4.0 cooled sCMOS camera using a TIRF APON ×60/1.49 and U Plan S Apo ×100/1.4 oil objective with the Live Acquisition 2.5 software were used for capturing images. Images were processed in the OMERO biological image data management system. All images are representative of at least three independent sets of experiments. Post-acquisition colouring of the channels was performed according to artistic preference.

### Cell culture

HeLa CCL2, HEK293A and U2OS cells were cultured at 37 °C and 5% CO_2_ in high-glucose Dulbecco’s modified Eagle’s medium (Sigma-Aldrich) supplemented with 10% fetal bovine serum (Biowest), 2 mM l-glutamine (Gibco), 1 mM sodium pyruvate (Sigma) and 1×penicillin–streptomycin (Sigma). INS-1 832/13 cells were cultured at 37 °C and 5% CO_2_ in RPMI-1640 medium (Sigma) supplemented with 2 mM l-glutamine (Gibco), 1 mM sodium pyruvate (Sigma), 10 mM HEPES (BioConcept), 0.05 mM EmbryoMax β-mercaptoethanol (Sigma) and 10% fetal bovine serum. The HeLa CCL2 (ATCC CCL-2) and HEK293A (ATCC CVCL-6910) cell lines were a gift from M. Spiess; their identities were authenticated by short-tandem-repeat analysis by Microsynth AG (last in 2021). The U2OS cell line (ATCC HTB-96) was a gift from M. Hall. The INS-1 832/13 cell line was purchased from Sigma-Aldrich (catalogue number SCC207). All cell lines were confirmed to be mycoplasma-negative by PCR. For transient cell transfections, the cells were plated into six-well plates to reach 70% confluency the following day and transfected with 1 µg plasmid DNA complexed with Helix-IN transfection reagent (OZ Biosciences). For small interfering RNA (siRNA) knockdown experiments, cells were transfected with a final concentration of 50 pmol Silencer Select siRNA targeting *PDIA6* (siRNA1, GAAUGUUCUGGACAGUGAAtt, Ambion, s19715 and siRNA2, GGCAGUGAAUGGUCUGUAUtt, Ambion, s19716) using RNAiMAX (Invitrogen) as per the manufacturer’s protocol. The cells were replated onto coverslips or an imaging chamber the following day and fixed or imaged 24–48 h post transfection. For stress treatments, the cells were treated with 1 μM tunicamycin, 1.25 μg ml^−1^ thapsigargin or 8 μM cyclopiazonic acid for the specified duration. For washout experiments, the cells were treated with 1 μM tunicamycin, 1.25 μg ml^−1^ thapsigargin or 8 μM cyclopiazonic acid for 10 h, washed with PBS and cultured in fresh medium overnight. For experiments with blocked proteasomal degradation, the cells were treated with 5 μM MG-132 for 18 h.

### Plasmid generation for cell transfection

To ensure ER localization, the KDEL signal sequence (present in native PDIA6) was cloned at the C terminus of PDIA6–tGFP (Origene, RG201710) using a NEBuilder HiFi Assembly cloning kit (New England Biolabs) with primers designed by the NEBuilder assembly tool following the manufacturer’s instructions. For simplicity, we refer to this plasmid as PDIA6–GFP. The C-terminal tail of PDIA6 was truncated by removing the last twelve amino acids from its sequence in the PDIA6-tGFP-KDEL plasmid using a NEB site-directed mutagenesis kit following the manufacturer’s instructions and primers selected using the NEBaseChanger tool. The PDIA6-EGFP-KDEL plasmid for transient transfections was generated from the PDIA6-tGFP-KDEL plasmid and the EGFP insert from ACE2–EGFP (Addgene, catalogue number 154962), using a NEBuilder HiFi assembly cloning kit (New England Biolabs) with the primers designed by the NEBuilder Assembly Tool following the manufacturer’s instructions. The QuikChange II mutagenesis protocol (Stratagene) was used to introduce the mutations K159N and K160N, and R124Q, K159N and K160N. Mutagenesis of L128A, L131A, L134A, V135A and L139A; R142Q, K150N and R153Q; R142Q, K150N, R153Q, K159N and K160N; and introduction of silent mutations in PDIA6 WT and PDIA6 5× linker mutant (for siRNA + transfection) was performed by GenScript. PDIA6 L128A, L131A, L134A, V135A and L139A has been characterized to be monomeric^23^ and is hence referred to as ‘PDIA6 monomer’. A gene encoding human pre-proinsulin, followed by a C-terminal myc–his A tag was synthesised by BioCat. The genes were inserted using BamHI and XhoI restriction enzymes into a pcDNA3.1(+) expression vector (Novagen). The QuikChange II mutagenesis protocol (Stratagene) was used to introduce the Akita proinsulin mutation C96Y. Hamster BiP–mCherry was a gift from E. Snapp (Addgene plasmid 62233) and mutated to the human sequence, and mApple–Sec61 was a gift from J. Lippincott-Schwartz. All PCR primers were obtained from Microsynth. The plasmids were purified using the Zyppy plasmid mini prep kit from Zymo Research and mutations were confirmed by sequencing by Microsynth AG.

### Generation of PDIA6–HaloTag cell line

To generate the C-terminally Halo-tagged PDIA6 HeLa CCL2 cell line, the PDIA6 genomic locus was targeted shortly after the stop codon with the following guide RNA: 5′-CACCGAGTTGTGAGAGCCACAACAG-3′. The guides were synthesized by Microsynth and cloned into the hSpCas9 pX330 plasmid (Addgene plasmid 42230) by annealing oligonucleotides and ligation into the vector, which was linearized with BbsI. The homology repair was designed as follows: 500 bp left homology arm–glycine-serin linker (GSGSGSGSGS)–HaloTag–linker (PSRLEEELRRRLTEP)–ER retention sequence (KDEL)–PolyA–G418/geneticin resistance cassette–500 bp right homology arm. The coding sequence of the G418 resistance cassette, PolyA and the HaloTag were provided by F. Bottanelli and the homology repair plasmid was synthesized by Genscript. Both plasmids were confirmed by sequencing (Microsynth).

HeLa CCL2 cells at 70% confluency were transfected with 0.5 µg pX330 plasmid with the PDIA6 guide and 0.5 µg homology repair plasmid using Helix-IN transfection reagent (OZ Biosciences). Three days post transfection, 1.5 mg ml^−1^ G418 (Thermo Fisher) was added; the medium was exchanged every 2–3 days with fresh medium containing 1.5 mg ml^−1^ G418 until selection was complete (two weeks).

### Generation of PDIA6-knockout cell lines

To generate a CRISPR–Cas9-mediated PDIA6-knockout cell line, the PDIA6 genomic locus was targeted with the following guide RNAs: 5′-GCTCGTGAAGGATCGCCTCG-3′ (gRNA1, targeting exons 5–7, depending on the splice variant) and 5′-AGACCGCGTTGGGGATTGGA-3′ (gRNA2, targeting exons 11–13, depending on splice variant). The guides were synthesized by Microsynth and cloned into px458-mCherry (gRNA1) and px459-Puro (gRNA2) vectors by annealing oligonucleotides and ligation into the vector, which was linearized with BbsI. The plasmid was confirmed by sequencing (Microsynth).

HeLa CCL2 and Hek293A cells at 70% confluency were transfected on a 10 cm dish with px458-mCherry (gRNA1) plasmid (2.5 µg per dish) and px459-Puro (gRNA2) plasmid containing the PDIA6 guides (2.5 µg per dish) using Helix-IN transfection reagent (OZ Biosciences). For selection, the cells were treated with 1.5 µg ml^−1^ puromycin one day post transfection for 24 h. FACS sorting (mCherry^+^) was carried out 48 h after transfection. The cells were trypsinized and resuspended in cell-sorting medium (2% FCS and 2.5 mM EDTA in PBS) and sorted on a Fortessa flow cytometer. Individual mCherry^+^ cells were collected, seeded into a 96-well plate and expanded. PDIA6 expression was determined by western blots. All of the surviving clones were positive for PDIA6, indicating that PDIA6 is essential.

### Immunostaining

HeLa cells were plated onto coverslips 24 h before fixation or stress treatment. At specified time points, the cells were fixed with 4% paraformaldehyde, permeabilized with 0.1 % Triton X-100, blocked in PBS containing 5% fetal bovine serum and stained with polyclonal anti-PDIA6 (1:200; GeneTex, GTX33397), monoclonal anti-GRP78/BiP (1:200; Invitrogen, 1H11-1H7; lot: XI3695781), polyclonal anti-calreticulin (1:200; Proteintech, 27298-1-AP), polyclonal anti-DNAJB11/ERdj3 (1:200; Thermo Fisher, 15484-1-AP), monoclonal anti-Grp94 (1:200; Enzo, SPA-850; lot: 103418), anti-PDIA1 (1:200; GeneTex, GTX2279), polyclonal anti-calnexin (1:200; Enzo, ADI-SPA-860; a gift from the Speiss laboratory), followed by Alexa Fluor (AF)488- or AF633-conjugated goat anti-rabbit, AF633-conjugated goat anti-mouse, AF488- or AF633-conjugated goat anti-rabbit, AF488- or AF633-conjugated goat anti-rabbit, AF488-conjugated goat anti-rat or AF546-conjugated donkey anti-rat, AF633-conjugated goat anti-mouse and AF488- or AF633-conjugated goat anti-rabbit (all 1:500), respectively. Proinsulin was visualized within the condensates by staining pre-proinsulin–myc–his-transfected HeLa cells with anti-myc clone 9E10 (1:2,000; Sigma-Aldrich, M4439; lot: 029M4849V) primary antibody, followed by AF633-conjugated anti-mouse (1:500). PDIA6 in the PDIA6–HaloTag knock-in HeLa CCL2 cell line was stained with polyclonal anti-HaloTag (1:250; Promega, G928A), followed by AF488-conjugated goat anti-rabbit (1:500). Overexpressed PDIA6–GFP was imaged in the 488 channel using the eGFP signal. Coverslips were mounted onto glass slides with Fluoromount G (Southern Biotech) and sealed with nail polish.

### Microscopy of fixed cells

Confocal images were acquired using an Olympus Fluoview FV3000 system and an UPLSAPO ×60/1.30 objective with silicone oil using the FV3000 (FV21S-SW version 2.5.1) system software. Laser intensities were at 0.5–3.0% for both the 488 (AF488) and 640 nm (AF633) wavelengths. Sampling speed was 8.0 μs pixel^−1^. All images are representative of at least three independent sets of experiments. All images for corresponding experiments were processed with the same settings to insure comparable results. Post-acquisition colouring of the channels was performed according to artistic preference.

### Live-cell imaging

For live-cell imaging, cells were seeded on an imaging chamber (ibidi μ-slide) 24 h before data acquisition. Knock-in cells expressing PDIA6–Halo–KDEL fusion were labelled for 1 h using 200 nM JFX-554 (Promega) in the cell culture medium. After the labelling, the cells were rinsed and incubated for at least 1 h in culture medium. Live-cell imaging was performed in complete growth medium lacking phenol red at 37 °C with 5% CO_2_ on a wide-field microscope (FEI MORE) with a Hamamatsu ORCA flash 4.0 cooled sCMOS camera using a TIRF APON ×60/1.49 oil objective and an Olympus ‘SpinD’ spinning-disc confocal microscope with a Hamamatsu ORCA-Fusion, sCMOS using an UPL APO ×60/1.5 oil objective. Laser intensities were set to 5–10% for the 488 nm wavelength. The system was preheated overnight (MORE) or 1 h (SpinD) to 37 °C to prevent thermal fluctuations. All images are representative of at least three independent sets of experiments. Images of the movies were recorded every 0.5–1.0 s. Post-acquisition colouring of the channels was performed according to artistic preference.

### FRAP

The FRAP experiments were performed on an Olympus ‘SpinD’ spinning-disc confocal microscope with a Hamamatsu ORCA-Fusion, sCMOS using an UPL APO ×60/1.5 oil objective. PDIA6 droplets and condensates were bleached using a 488 nm laser with 100% intensity (Rapp OptoElectronic Firefly). A total ten images were obtained prebleaching, and 200 images were recorded every 1.0–1.83 s (in vitro) or 0.5–1.0 s (in vivo) post bleaching. Sec61 was bleached using a 488 nm laser with 100% intensity (Rapp OptoElectronic Firefly). A total of ten images were obtained prebleaching and 30–200 images were recorded post bleaching with a time interval of 1.5 s (PDIA6–GFP WT co-transfected cells) or 2.3 s (PDIA6–GFP 5× linker mutant co-transfected cells). Intensities of FRAP regions were extracted with the Fiji 1.52p software tool, background corrected and reported relative to the prebleaching time point (PDIA6–GFP) or normalized as a percentage of recovery (mApple–Sec61).

### Image analysis

Images were analysed using Omero webserver 5.26.0, Fiji 1.52p and Python 3.9.

#### Morphological analysis and quantification of condensates in cells

Cell images were processed using a custom Python 3.9 script with OpenCV 4.10.00 and NumPy 1.26.4 to detect phase-separated condensates based on predefined morphological parameters. The detected condensates were quantified using circularity-based segmentation, enabling the extraction of key morphological properties, including size, shape and number, for further analysis.

#### Morphological analysis and quantification of droplets in vitro

Microscopy images were processed using a custom Python 3.9 script with OpenCV 4.10.00 and NumPy 1.26.4, employing the Hough Transform to detect circular droplets. The number of detected droplets per image was then quantified, enabling further analysis of their distribution.

#### Quantification of endogenous PDIA6 and BiP enrichment in condensates in cells

Cell images were processed using a custom Python 3.9 script with OpenCV 4.10.00 and NumPy 1.26.4 for cell segmentation via Otsu thresholding and condensate detection. Following feature extraction, the relative intensities of condensed and dispersed protein populations in each cell were quantified to assess protein enrichment in condensates.

#### Analysis of fluorescence co-localization

Pearson correlation coefficients were determined using the Fiji 1.52p ‘JaCOP’ plugin from 10–15 cells (see Supplementary Table 2). Statistical significance was calculated using a two-tailed Student’s *t*-test. The fluorescence intensity histograms were extracted using the Fiji 1.52p software ‘plot profile’ tool.

### Western blotting and antibodies

To assess PDIA6 and BiP expression levels, HeLa CCL2 cells were lysed in lysis buffer (1% Triton X-100, 150 mM NaCl, 20 mM Tris pH 7.5, 1 mM EDTA, 1 mM EGTA and protease inhibitor) and denatured in Laemmli buffer at 65 °C for 10 min. The three biological replicates were prepared and lysed independently (*n* = 3). Samples were resolved by 10% SDS–PAGE and transferred onto nitrocellulose membrane (Amersham). The membranes were blocked with TBST (20 mM Tris (pH 7.6), 150 mM NaCl and 0.1% Tween 20) with 5% non-fat dry milk for 30 min and incubated overnight with primary polyclonal anti-PDIA6 (1:2,000; GeneTex, GTX33397), monoclonal primary anti-GRP78/BiP (1:2,000; Invitrogen, 1H11-1H7; lot: XI3695781) or primary monoclonal anti-α-tubulin (1:2,000; Sigma, T5168; lot: 0000242797) at 4 °C, followed by 2 h incubation with horseradish peroxidase (HRP)-conjugated secondary antibody (1:20,000; anti-mouse or anti-rabbit; Invitrogen, 31430 and 31460) in TBST. Chemiluminescence signals were detected using Immobilon western HRP substrate (Advansta) and imaged using a FusionFX imaging system (Vilber Lourmat).

To asses insulin processing in HeLa cells, the cells were transfected with pre-proinsulin (and PDIA6 constructs). After 24 h, the cells were washed with preheated 1×PBS and 4×OPTIMEM GlutaMAX (Gibco), and incubated in OPTIMEM GlutaMAX (16 h). To assess insulin processing in INS-1 cells, the cells were washed 36 h post transfection (of PDIA6 constructs only) with HBSS (Gibco) + 2.5 mM glucose (Gibco) for 1 h and subsequently incubated with HBSS + 2.5 mM glucose for 2 h. The medium was collected, spun down (10 min, 13,000 rpm, 4 °C) and the supernatant was precipitated by the addition of 100% trichloroacetic acid (one volume trichloroacetic acid to four volumes protein). After 10 min incubation on ice, the samples were spun down (5 min, 13,000 rpm, 4 °C) and washed twice with cold acetone. The dried pellet was dissolved in 30 μl Laemmli buffer by vortexing and boiling (10 min, 300 rpm, 95 °C). The cells were lysed in RIPA buffer and denatured in Laemmli buffer at 65 °C for 10 min. The four biological replicates were prepared and lysed independently (*n* = 4). Protein levels were measured using a BCA assay (Merck) and the volumes of the precipitate and lysates were adjusted to equal lysate protein concentrations. Samples were resolved by 4–20% SDS–PAGE (BioRad) and transferred onto nitrocellulose membranes (BioRad). The lysate blot was processed as described above. The blot of the secreted proteins was preblocked with TBST containing 5% non-fat dry milk for 5 min and the samples were fixed by incubation with TBST containing 0.2% glutaraldehyde for 15 min. The membrane was washed in TBST, blocked with TBST containing 5% non-fat dry milk for 30 min and incubated overnight with monoclonal anti-proinsulin (for secretion, 1:1,000; Invitrogen, MA1-83256; lot: 160454; for lysate, 1:2,000; Cell Signaling, 3014; lot: 10) in Can get signal solution (Toyobo) at 4 °C, followed by 2 h incubation with HRP-conjugated secondary antibody (1:20,000; anti-mouse or anti-rabbit; Invitrogen, 31430 or 31460, respectively) in Can get signal solution. Chemiluminescence signals were detected using Immobilon western HRP substrate (Advansta) and imaged using a FusionFX imaging system (Vilber Lourmat).

### MTT assay

HeLa cells were seeded in 96-well plates 24 h after transfection. After 24 h, MTT (Merck) was added at the indicated time points to a final concentration of 500 μg ml^−1^ and incubated for 2 h at 37 °C. The medium was removed and the crystals were dissolved in acidic (0.08 M HCl) isopropanol. The absorbance (*A*_550nm_ − *A*_690nm_) was measured on a plate reader. All experiments were repeated in experimental and independent biological triplicates. Significance was calculated using a two-tailed Student’s *t*-test (*n* = 9).

### Quantitative PCR with reverse transcription

RNA was extracted and purified from cells using an RNeasy kit according to the manufacturer’s instructions. Complementary DNA was reverse-transcribed using GoScript reverse transcriptase primed with a mix of Oligo(dT)s and random hexamers (Promega). Real-time PCR was then performed using GoTaq qPCR master mix (Promega) and primers specific for sXBP1 (sXBP1, 5′-GCTGAGTCCGCAGCAGGT-3′; β-actin, 5′-CACCATTGGCAATGAGCGGTTC-3′). The expression of sXBP1 was normalized to that of β-actin. For PDIA6 WT-transfected cells, the sXBP1 levels were still below detection limit after 40 cycles of quantitative PCR and the *C*_t_ was thus set to 40 for statistical analysis. The significance was calculated from three independent biological replicates using a two-tailed Student’s *t*-test (*n* = 3).

### Aggresome detection

For microscopy, PDIA6–GFP (WT and 5× linker mutant)-transfected HeLa cells were plated onto coverslips 24 h before fixation. Aggresomes were visualized using the PROTEOSTAT aggresome dye following the manufacturer’s protocol. Briefly, the cells were fixed for 30 min with 4% formaldehyde solution at room temperature, incubated with permeabilizing solution (1×assay buffer, 0.5 mM EDTA and 0.05% Triton X-100) on ice for 30 min, incubated for 30 min with the PROTEOSTAT detection reagent at room temperature in the dark, mounted on a microscopy slide with Fluoromount G (Southern Biotech) and sealed with nail polish.

For aggresome detection analysis by flow cytometry, PDIA6–GFP (WT and 5× linker mutant)-transfected HEK293A cells and controls were pelleted, fixed with 4% formaldehyde solution for 30 min at room temperature and permeabilized (1×assay buffer, 0.5 mM EDTA and 0.05% Triton X-100), resuspended in FACS buffer (PBS containing 2% FCS and 0.05 μM EDTA) with PROTEOSTAT dye (1:1,250 dilution) and incubated for 30 min at room temperature in the dark. A Fortessa flow cytometer was used to measure mCherry fluorescence (PROTEOSTAT dye) of GFP-positive cells. Data were analysed using FlowJo 10.9.0. The gating strategy is in Supplementary Fig. 2.

### Structural models

The BiP structural model in the ADP undocked states was obtained by homology modelling using the SWISS-MODEL server and the DnaK undocked conformation (PDB 2kho)^61^ as a template. The structural model of PDIA6 FL has been modelled as a chimaera of the individual domain crystal structures (domain a^0^, PDB: 4ef0; domain a, PDB: 4gwr; domain b, PDB: 8cpq, determined in this study). The linker connecting domain a^0^ and a, and the C-terminal tail were modelled as disordered based on the NMR data presented in Fig. 2 and Extended Data Figs. 3,4. The interface between the domain a^0^ monomers is based on the crystal structure of domain a^0^ (PDB: 4ef0) and confirmed by NOE contacts between the domain, as presented in Fig. 2. Importantly, the distances V75–L131 (7.3 Å), V75–L134 (11.4 Å), V75–V135 (12.7 Å) and L72–L131 (7.8 Å) within the monomer are too large to have (strong) intramolecular methyl NOE contacts, indicating that the resulting NOEs are intermolecular NOEs. The measured NOE crosspeak intensities fit to the expected intermolecular NOE intensities based on the distances calculated from the crystal structure (PDB: 4ef0; V75–L131, 4 Å; V75–L134, 4 Å; V75–L135, 7.1 Å and L72–L131, 7.5 Å). The interface between domains a and b was modelled using ESMFold^62^ based on the NOE contact between the domains presented in Fig. 2. The binding interface of the linker to domain b was modelled based on the experiments presented in this paper.

### Structural comparison of helix α4

The following protein structures were used to compare the length of helix α4: PDB: 4ekz (PDIA1 a and a′), 2dmm (PDIA3 a′), 2dj1 (PDIA4 a^0^), 2dj2 (PDIA4 a), 2dj3 (PDIA4 a′), 4ef0 (PDIA6 a^0^) and 4gwr (PDIA6 a).

### Estimation of PDIA6 Ca^2+^ binding affinity

To determine the PDIA6 Ca^2+^ binding affinity (*K*_D_(Ca^2+^)), the Ca^2+^-dependent formation of droplets in the presence of a crowding agent was used as a direct measurable of the Ca^2+^ binding to PDIA6. The number of unlabelled PDIA6 droplets was quantified for a Ca^2+^ concentration between 100 μM and 1,000 μM from differential-interference-contrast microscopy images and fitted using nonlinear regression to the Hill equation binding model (equation (1)) to determine the *K*_D_(Ca^2+^).

1 $${\mathrm{nc}}={{\mathrm{nc}}}_{\min }+\frac{{{\mathrm{nc}}}_{\max }-{{\mathrm{nc}}}_{\min }}{1+{\left(\frac{{K}_{\rm{D}}{({\mathrm{Ca}}^{2+})}}{[{\mathrm{Ca}}^{2+}]}\right)}^{n}}$$

where nc is the number of condensates and *n* is the Hill coefficient.

### MD simulations to study flexibility of domain a–b linker residues

#### Initial structures and equilibration

The structure model was generated with ESMFold^61^ for the full-length protein and manually trimmed to residues 161–419 to have a more compact, yet fully functional, construct. To generate equilibrated starting structures for the pulling simulations, each structure (WT and mutant) was placed in a dodecahedral box of TIP4 water, to which 150 mM NaCl was added, including neutralizing counterions. Following steepest descents minimization, each of the systems was equilibrated for 100 ps under a constant-pressure (constant number of particles, pressure, and temperature) ensemble, with position restraints applied to peptide heavy atoms throughout. Protein and non-protein atoms were coupled to separate temperature coupling baths and temperature was maintained at 300 K using the velocity rescale coupling method. Pressure was controlled using exponential relaxation pressure coupling to maintain pressure isotropically at 1.0 bar. Short-production molecular dynamics (MD) simulations were conducted for 100 ps in the absence of any restraints to relax the initial structures. All simulations were conducted using the GROMACS package (version 2024.3).

#### Pulling simulations and umbrella sampling

Structures at the end of each of the previous relaxed trajectories (WT and ^274^PPP–^274^GGG) were used as starting configurations for pulling simulations. To make the immobile reference for the pulling simulations, the coordinates of Cα atoms for residues 161–272 were restrained. Residues 284–419 were pulled away from the structure over 500 ps, using a spring constant of 1,000 kJ mol^−1^ nm^−2^ and a pull rate of 0.1 Å ps^−1^. A final centre-of-mass collective variable distance of approximately >6.0 Å was achieved. From these trajectories, snapshots were taken to generate the starting configurations for the umbrella sampling windows. A distribution of sampling windows was used, such that the window spacing was 1 Å centre-of-mass separation, and resulted in each WT and mutant case in nine windows. In each window, 1 ns of MD was performed for a total simulation time of 9 ns utilized for umbrella sampling. Analysis of results was performed with the weighted histogram analysis method (WHAM).

### Statistics and reproducibility

The number of data points in each experiment is specified in the figure legends or in Supplementary Tables 2 (Fig. 5e) and 3 (microscopy images and western blots). All in-cell data are based on multiple experiments with independent biological replicates. No statistical methods were used to pre-determine sample sizes but our sample sizes are similar to those reported in previous publications. Data distribution was assumed to be normal but this was not formally tested. A two-sided unpaired Mann–Whitney test was used for statistical analyses of the number of condensates per cell and condensate area. Statistical significance for all other analyses was calculated using a two-tailed Student’s *t*-test. Statistical analyses were performed using Excel 16.97 or GraphPad Prism 9. Details are provided in the figure legends. Where statistical comparisons were performed, specific *P* values are indicated on the graphs. No samples or data points were excluded. The experiments were not randomized. The investigators were not blinded to the conditions of the experiments during data analysis.

### Reporting summary

Further information on research design is available in the Nature Portfolio Reporting Summary linked to this article.

## Online content

Any methods, additional references, Nature Portfolio reporting summaries, source data, extended data, supplementary information, acknowledgements, peer review information; details of author contributions and competing interests; and statements of data and code availability are available at 10.1038/s41556-025-01730-w.

## Supplementary information

Supplementary Information Supplementary Figs. 1–6.

Reporting Summary

Supplementary Tables 1–3 Table 1. Statistics on diffraction data and refinement of PDIA6 domain b (residues 274–427). *Values in parenthesis are for the highest resolution shell. Table 2. Co-localization of ER chaperones in PDIA6 condensates. PCC for pixel intensity of the indicated proteins (protein 1 and protein 2) in 10–15 cells (*n*). Table 3. Number of independent experiments with similar outcomes. All microscopy images and western blots are representative of the results of all independent repetitions. No data were excluded.

Supplementary Video 1 Fusion event of PDIA6–GFP condensates in HeLa cells.

Supplementary Video 2 Fission event of PDIA6–GFP condensates in HeLa cells.

Supplementary Video 3 Fluorescence recovery after photobleaching of PDIA6–GFP condensates in HeLa cells, showing a first phase of fast recovery due to diffusion, followed by a second phase of slow recovery due to exchange of material.

Supplementary Video 4 HeLa cells stably expressing PDIA6–HaloTag fusion. PDIA6 is visualized by the HaloTag ligand JFX-554.

## Source data

Source Data Fig. 1 Statistical source data.

Source Data Fig. 3 Statistical source data.

Source Data Fig. 4 Statistical source data.

Source Data Fig. 5 Statistical source data.

Source Data Fig. 3 Unprocessed western blots.

Source Data Extended Data Fig. 1 Statistical source data.

Source Data Extended Data Fig. 2 Statistical source data.

Source Data Extended Data Fig. 2 Unprocessed western blots.

Source Data Extended Data Fig. 3 Statistical source data.

Source Data Extended Data Fig. 7 Statistical source data.

Source Data Extended Data Fig. 7 Unprocessed western blots.

Source Data Extended Data Fig. 8 Statistical source data.

Source Data Extended Data Fig. 10 Statistical source data.

Source Data Extended Data Fig. 10 Unprocessed western blots.

## Data Availability

PDIA6 methionine leucine valine methyl and his–domain a^0^, his–domain a and his–domain ab amide backbone resonance assignments have been submitted to the Biological Magnetic Resonance Data Bank under the following accession codes: 51863 and 51864. The crystal structure of the PDIA6 domain b has been deposited in the PDB under the accession code: 8cpq. Previously published PDB structures that were re-analysed here are available under the following accession codes: 4ekz (10.2210/pdb4EKZ/pdb), 2dmm (10.2210/pdb2DMM/pdb), 2dj1 (10.2210/pdb2DJ1/pdb), 2dj2 (10.2210/pdb2DJ2/pdb), 2dj3 (10.2210/pdb2DJ3/pdb), 4ef0 (10.2210/pdb4EF0/pdb), 4gwr (10.2210/pdb4GWR/pdb), 5kn3 (10.2210/pdb5KN3/pdb) and 2kho (10.2210/pdb2KHO/pdb). Domain delimitations for the generation of PDIA6 subconstructs were obtained from the UniProt database Q15084 (ref. ^63^). Guide RNAs were designed using the human reference genome GRCh38 (RefSeq accession: NC_000001.11). Specific target sequences were identified using CRISPOR^64^ and confirmed against transcript ENST00000272227. Microscopy images have been deposited on *Zenodo* at 10.5281/zenodo.15497399 (ref. ^65^). All other data supporting the findings of this study are available from the corresponding author on reasonable request. Source data are provided with this paper.

## Code availability

Custom Python scripts used in this paper for image quantification are available without access restriction at 10.5281/zenodo.15424556 (ref. ^66^).
