# Supplementary material for: A multichaperone condensate enhances protein folding in the endoplasmic reticulum
Source: Nat Cell Biol. 2025 Aug 11;27(9):1422–30. doi: 10.1038/s41556-025-01730-w (PMC12431857; doi:10.1038/s41556-025-01730-w)
Supplement: Supplementary file 1 — Supplementary Figs. 1–6. [file 41556_2025_1730_MOESM1_ESM.pdf]

nature cell biology

Supplementary information https://doi.org/10.1038/s41556-025-01730-w

# A multichaperone condensate enhances protein folding in the endoplasmic reticulum

In the format provided by the authors and unedited

# Supplementary Information

**for**

# A multi-chaperone condensate enhances protein folding in the endoplasmic reticulum

Anna Leder[1], Guillaume Mas[1]*, Viktória Szentgyörgyi[1], Roman P. Jakob[1], Timm Maier[1], Anne Spang[1], Sebastian Hiller[1]*

**Affiliations**

[1]Biozentrum, University of Basel, 4056 Basel, Switzerland

*Corresponding authors. Email: guillaume.mas@unibas.ch, sebastian.hiller@unibas.ch

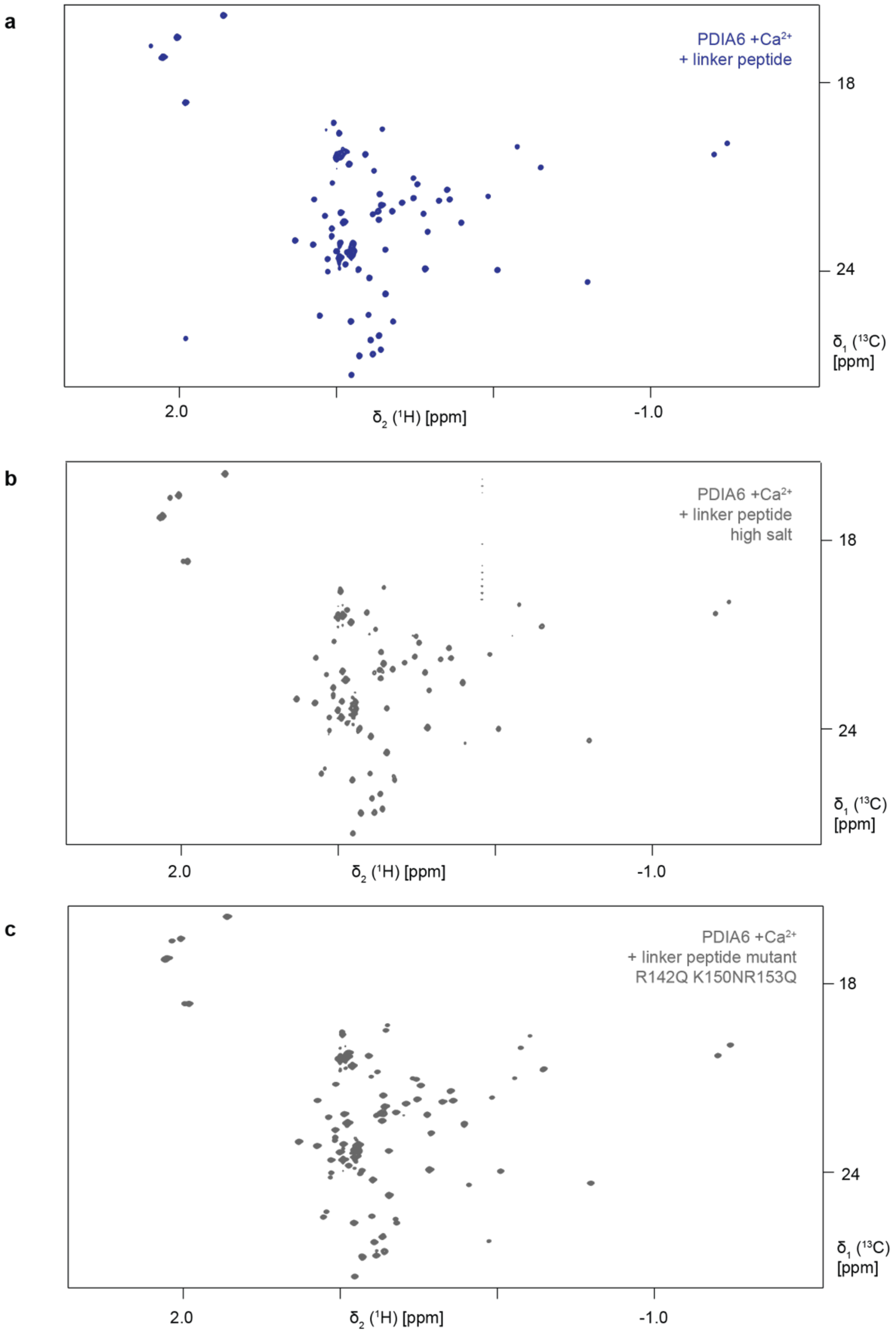


**Supplementary Fig. 1: Spectra corresponding to Fig. 3c,d. a,** 2D [$^{13}$C,$^{1}$H]-HMQC spectra of 100 µM methyl-labeled PDIA6 in presence of 10 mM $Ca^{2+}$ and 10 eq. linker peptide. **b,** 2D [$^{13}$C,$^{1}$H]-HMQC spectra of 100 µM methyl-labeled PDIA6 in presence of 10 mM $Ca^{2+}$, 10 eq. linker peptide and 1 M KCl. **c,** 2D [$^{13}$C,$^{1}$H]-HMQC spectra of 100 µM methyl-labeled PDIA6 in presence of 10 mM $Ca^{2+}$ and 10 eq. linker peptide mutated to R142Q/K150N/R152Q.

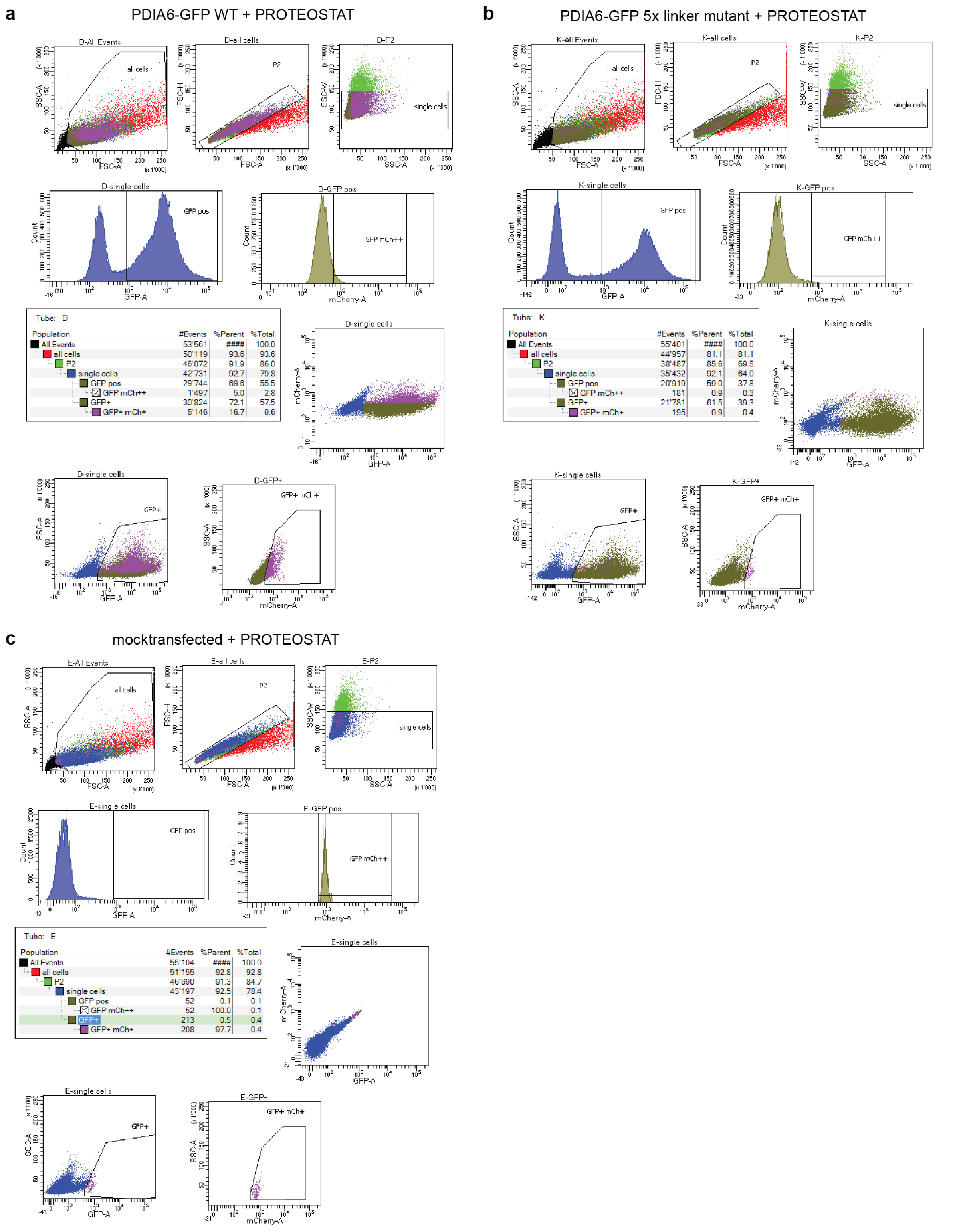


**Supplementary Fig. 2: Gating strategy for FACS sorting and mCherry measuremtns in Hek293A cells of data presented in Fig. 5h.** Gating strategy to measure cells with aggresomes in PDIA6-GFP WT (a), PDIA6-GFP 5x linker mutant (b) or mock (c) transfected cells, using the PROTEOSTAT® dye (mCherry). To exclude debris from the initial cell population, forward (FSC) versus side scatter (SSC) gating was applied (P2). Single cells were determined by using SSC area vs. SSC width (SSC-A/SSC-W) gating and

by FSC-A/FSC-H gating (single cells). From the single cell population, GFP+ cells were determined by gating GFP-A/SSC-A density plots (population GFP+). From the GFP+ cells, mCherry+ cells were determined by gating mCherry-A/SSC-A density plots (population GFP+mCherry+). More than 30’000 single cells were measured and the percentage of mCherry+ in GFP+ cells were determined in each sample (% Parent).

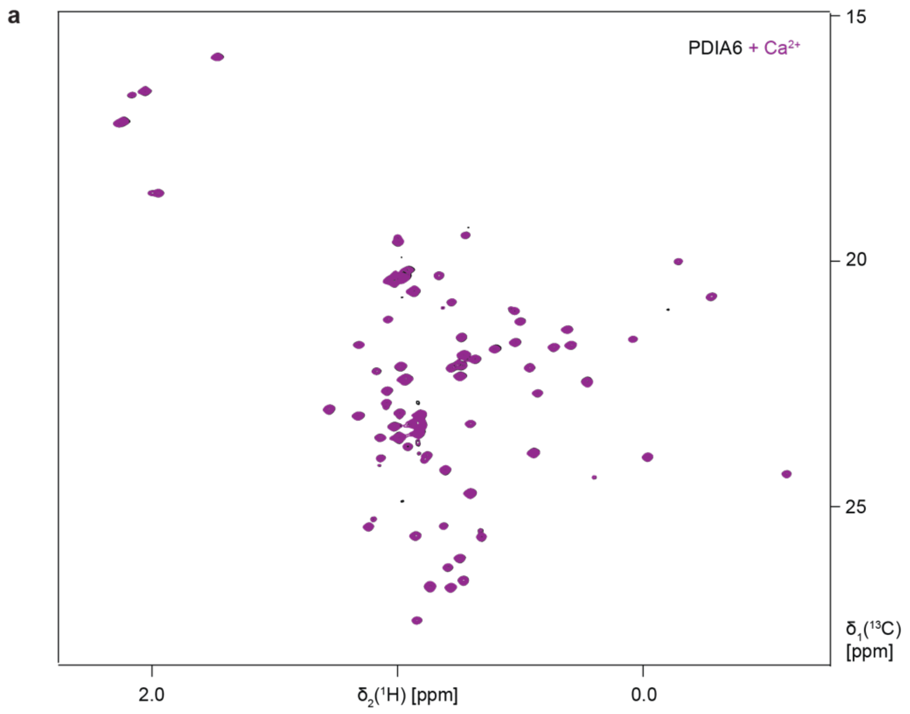


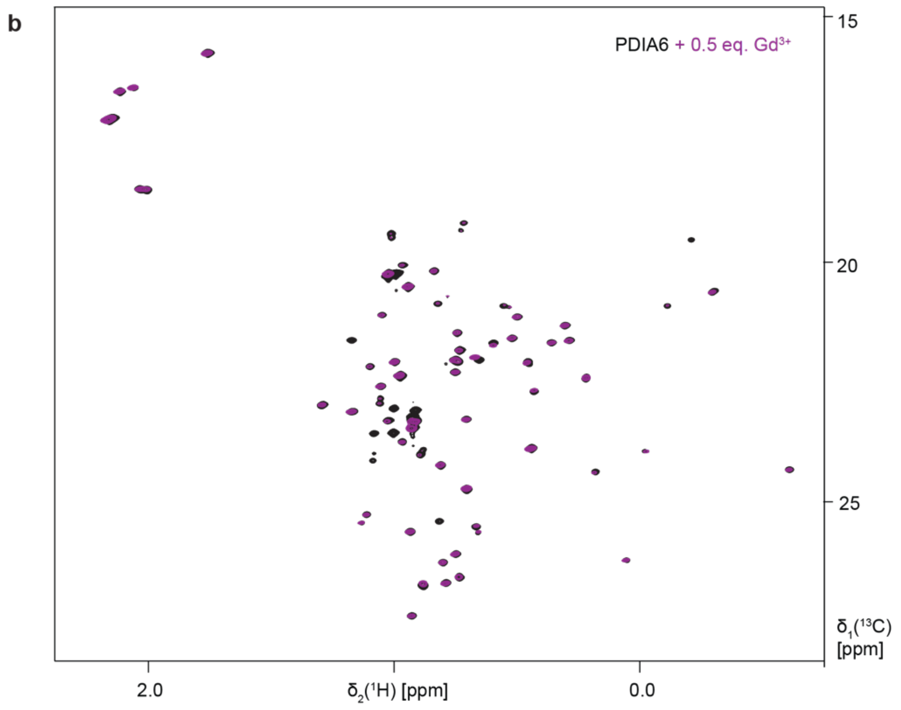


**Supplementary Fig. 3: Larger views of spectra presented in Extended Data Fig. 6a,b. a,** Overlay of 2D [$^{13}C$,$^{1}H$]-HMQC spectra of 100 µM methyl-labeled PDIA6 in absence (black) and presence of 10 mM $Ca^{2+}$ (purple). **b,** Overlay of 2D [$^{13}C$,$^{1}H$]-HMQC spectra of 100 µM methyl-labeled PDIA6 in absence (black) and presence of 0.5 eq. $Gd^{3+}$ (purple).

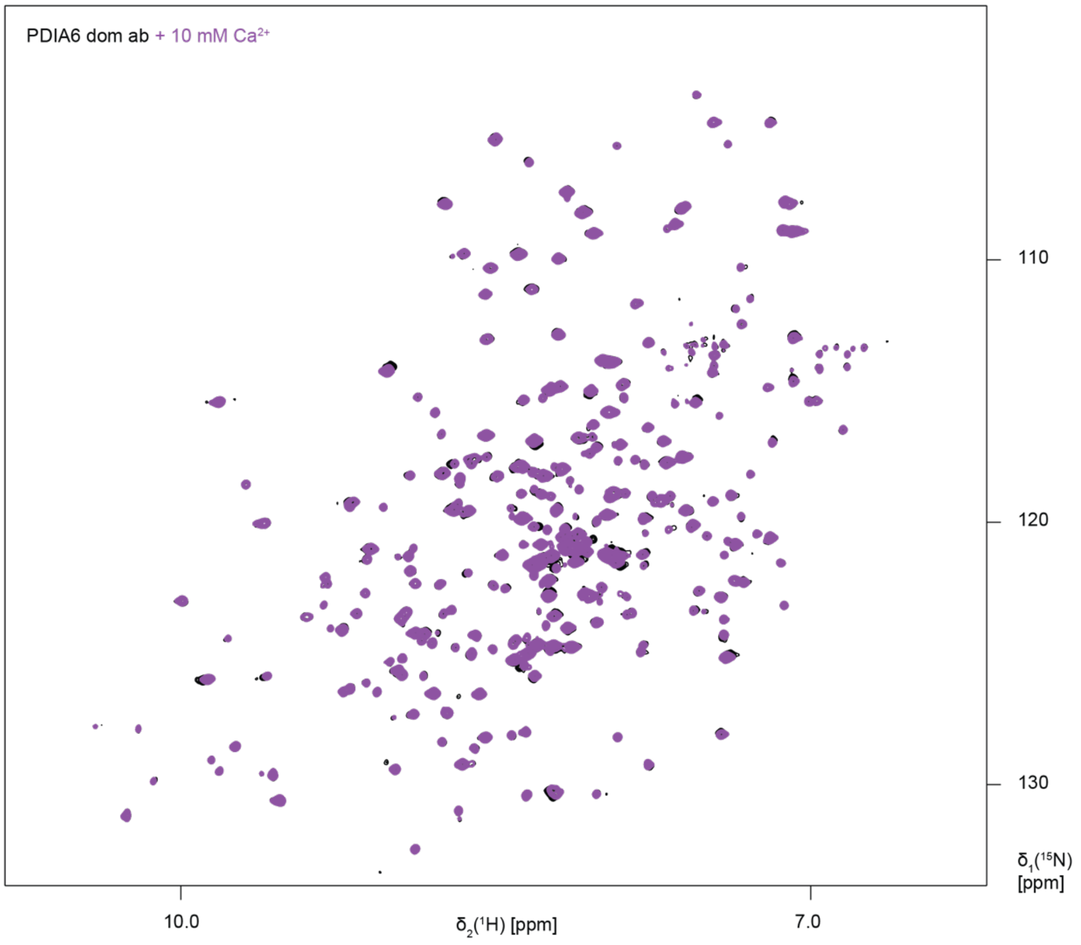


**Supplementary Fig. 4: Full spectra corresponding to sections presented in Extended Data Fig. 6i.** Overlay of of 2D [$^{15}$N,$^{1}$H]-HSQC spectra of 100 µM PDIA6 domain ab in absence (black) and in presence of 10 mM $Ca^{2+}$ (purple).

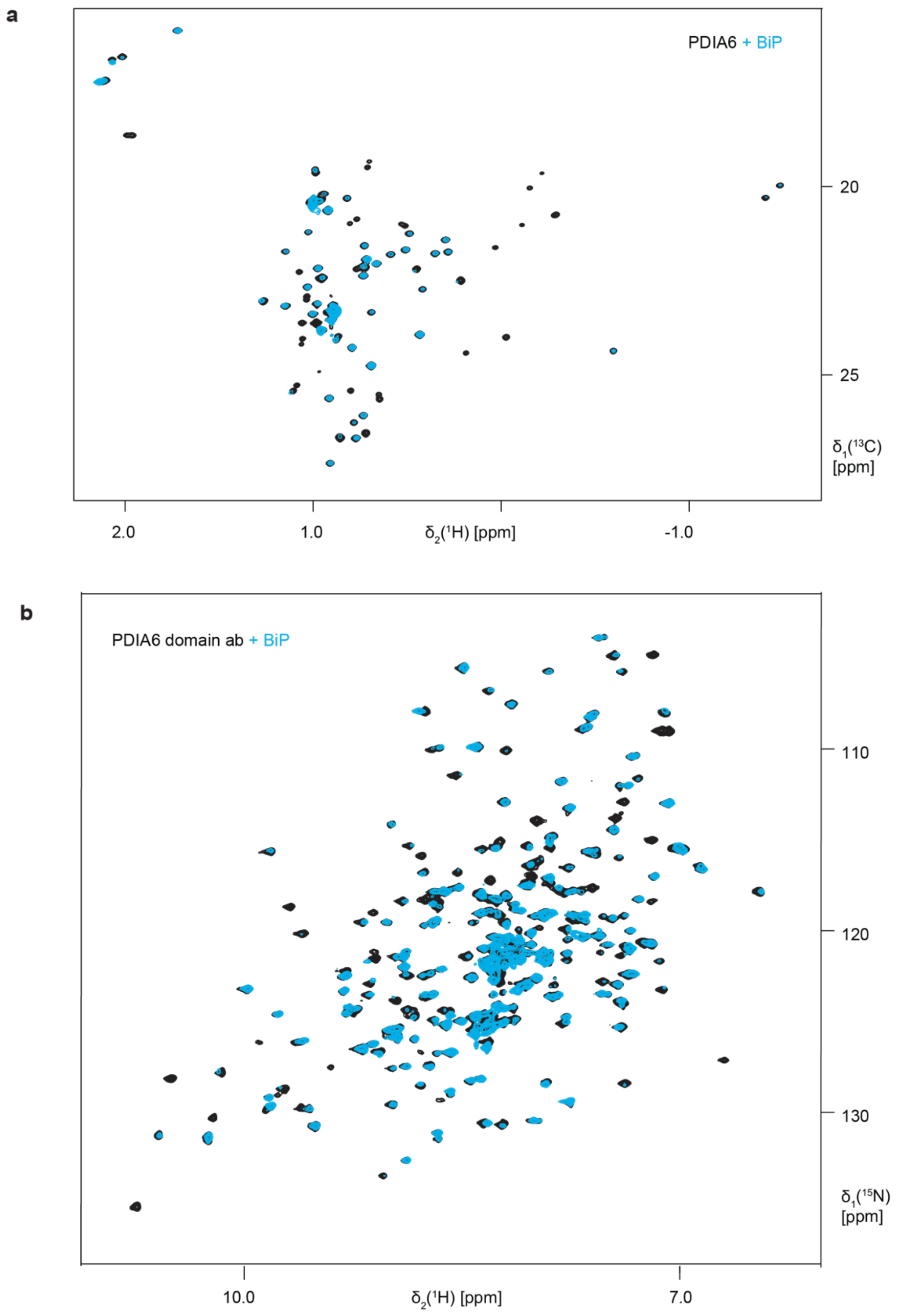


**Supplementary Fig. 5: Larger views of spectra presented in Extended Data Fig. 8d,e. a,** Overlay of 2D [$^{13}C$,$^{1}H$]-HMQC spectra of 100 µM PDIA6 in absence (black) and in presence of 2 eq. BiP (blue). **b,** Overlay of 2D [$^{15}N$,$^{1}H$]-HSQC spectra of 100 µM PDIA6 domain ab in absence (black) and in presence of 2 eq. BiP (blue).

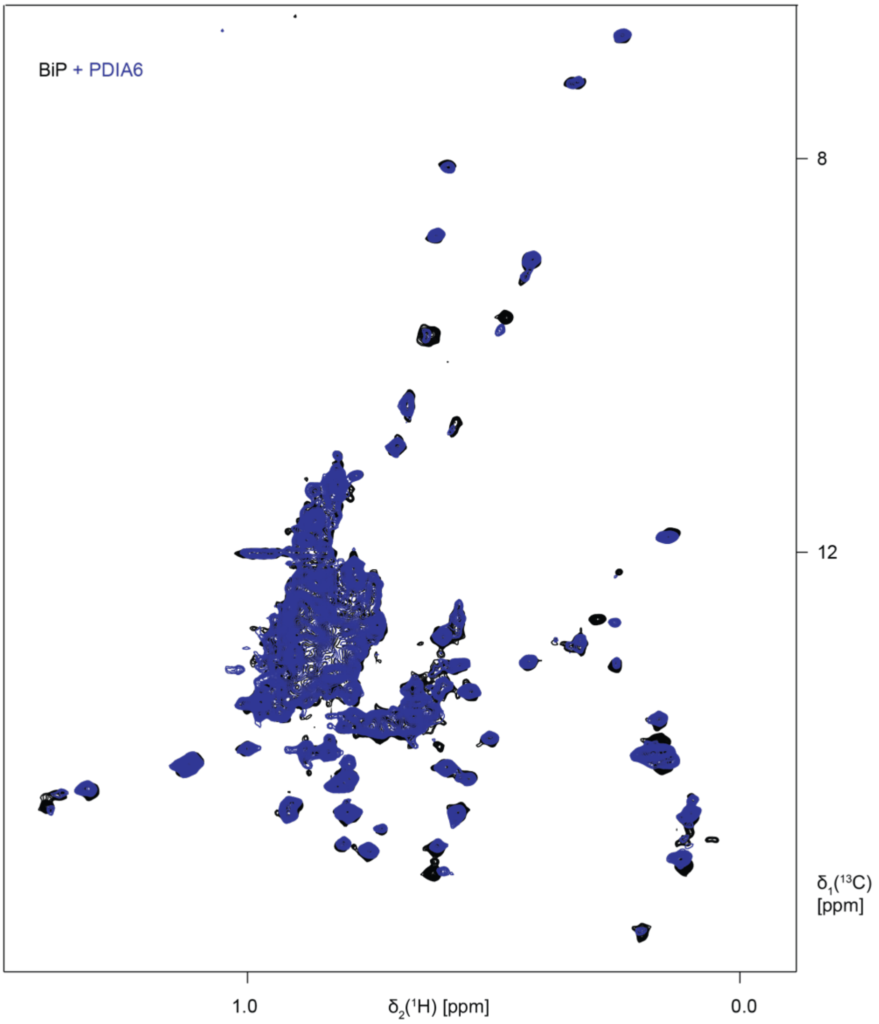


**Supplementary Fig. 6: Larger views of spectra presented in Extended Data Fig. 8i.** Overlay of 2D [$^{13}$C,$^{1}$H]-HMQC spectra of 100 µM methyl-labeled BiP in absence (black) and in presence of 2 eq. PDIA6 (blue).
