# Supplementary material for: A multichaperone condensate enhances protein folding in the endoplasmic reticulum
Source: Nat Cell Biol. 2025 Aug 11;27(9):1422–30. doi: 10.1038/s41556-025-01730-w (PMC12431857; doi:10.1038/s41556-025-01730-w)
Supplement: Supplementary file 2 — Reporting Summary [file 41556_2025_1730_MOESM2_ESM.pdf]

nature portfolio

Corresponding author(s): Sebastian Hiller and Guillaume Mas

Last updated by author(s): May 24, 2025

Reporting Summary

Nature Portfolio wishes to improve the reproducibility of the work that we publish. This form provides structure for consistency and transparency in reporting. For further information on Nature Portfolio policies, see our Editorial Policies and the Editorial Policy Checklist.

Statistics

For all statistical analyses, confirm that the following items are present in the figure legend, table legend, main text, or Methods section.

n/a	Confirmed	
☐	☒	The exact sample size (*n*) for each experimental group/condition, given as a discrete number and unit of measurement
☐	☒	A statement on whether measurements were taken from distinct samples or whether the same sample was measured repeatedly
☐	☒	The statistical test(s) used AND whether they are one- or two-sided<br>*Only common tests should be described solely by name; describe more complex techniques in the Methods section.*
☒	☐	A description of all covariates tested
☒	☐	A description of any assumptions or corrections, such as tests of normality and adjustment for multiple comparisons
☐	☒	A full description of the statistical parameters including central tendency (e.g. means) or other basic estimates (e.g. regression coefficient) AND variation (e.g. standard deviation) or associated estimates of uncertainty (e.g. confidence intervals)
☐	☒	For null hypothesis testing, the test statistic (e.g. *F*, *t*, *r*) with confidence intervals, effect sizes, degrees of freedom and *P* value noted<br>*Give P values as exact values whenever suitable.*
☒	☐	For Bayesian analysis, information on the choice of priors and Markov chain Monte Carlo settings
☒	☐	For hierarchical and complex designs, identification of the appropriate level for tests and full reporting of outcomes
☐	☒	Estimates of effect sizes (e.g. Cohen's *d*, Pearson's *r*), indicating how they were calculated

Our web collection on statistics for biologists contains articles on many of the points above.

Software and code

Policy information about availability of computer code

Data collection	NMR data were collected on Bruker spectrometers operated with TOPSPIN 3.6-4.0.<br>For the crystal structure data was collected at the SLS beamline X06DA (Swiss Light Source, Paul Scherrer Institute, Switzerland) at 100 K and were integrated, indexed and scaled using XDS software.<br>Western Blots were captured on a FUSION FX Vilber Lourmat using FusionCapt Advance<br>Microscopy: FEI MORE with a Hamamatsu ORCA flash 4.0 cooled sCMOS camera with Live Acquisition 2.5 software, Olympus Fluoview FV3000 system with FV3000 (FV21S-SW Version 2.5.1) system software<br>FlowJo Version 10.9.0
Data analysis	NMR: NMRPipe 2022.230.12.16 , CcpNmr Analysis version 2.5.2<br>Crystal structure: Phaser 1.0, Arp/Wrap 8.0, PHENIX 1.17, Coot 0.9.8<br>Microscopy: Fiji 1.52p and plugins therein, Omero webclient 5.26.0<br>Flow cytometry: FlowJo 10.9.0<br>MD Simulations: GROMACS 2024.3<br>Data analysis and statistical analysis: Excel 16.97 or GraphPad Prism 9, Python 3.9 with OpenCV 4.10.00 and NumPy 1.26.4<br>Custom Python scripts used in this manuscript for image quantification are available without access restriction at https://doi.org/10.5281/zenodo.15424556

For manuscripts utilizing custom algorithms or software that are central to the research but not yet described in published literature, software must be made available to editors and reviewers. We strongly encourage code deposition in a community repository (e.g. GitHub). See the Nature Portfolio guidelines for submitting code & software for further information.

Data

Policy information about availability of data

All manuscripts must include a data availability statement. This statement should provide the following information, where applicable:

- Accession codes, unique identifiers, or web links for publicly available datasets
- A description of any restrictions on data availability
- For clinical datasets or third party data, please ensure that the statement adheres to our policy

PDIA6 MLVpS methyl and his-domain a0, his-domain a and his-domain ab amide backbone resonance assignments have been submitted to the Biological Magnetic Resonance Data Bank under the following accession codes: 51863 and 51864. The crystal structure of the PDIA6 domain b has been deposited in the protein data bank under the accession code: 8cpq. Previously published pdb structures that were re-analysed here are available under accession codes: 4ekz [https://doi.org/10.2210/pdb4EKZ/pdb], 2dmm [https://doi.org/10.2210/pdb2DMM/pdb], 2dj1 [https://doi.org/10.2210/pdb2DJ1/pdb], 2dj2 [https://doi.org/10.2210/pdb2DJ2/pdb], 2dj3 [https://doi.org/10.2210/pdb2DJ3/pdb], 4ef0 [https://doi.org/10.2210/pdb4EF0/pdb], 4gwr [https://doi.org/10.2210/pdb4GWR/pdb], 5kn3 [https://doi.org/10.2210/pdb5KN3/pdb] and 2kho [https://doi.org/10.2210/pdb2KHO/pdb]. Domain delimitations for the generation of PDIA6 sub-constructs were obtained from uniport data base Q15084. Guide RNAs were designed using the human reference genome GRCh38 (RefSeq accession: NC_000001.11). Specific target sequences were identified using CRISPOR50 and confirmed against transcript ENST00000272227. Source data are provided with this study. Microscopy images have been deposited on Zenodo at https://doi.org/10.5281/zenodo.15497399. Custom Python scripts used in this manuscript for image quantification are available without access restriction at https://doi.org/10.5281/zenodo.15424556 (ref61). All other data supporting the findings of this study are available from the corresponding author on reasonable request.

Research involving human participants, their data, or biological material

Policy information about studies with human participants or human data. See also policy information about sex, gender (identity/presentation), and sexual orientation and race, ethnicity and racism.

Reporting on sex and gender	n/a
Reporting on race, ethnicity, or other socially relevant groupings	n/a
Population characteristics	n/a
Recruitment	n/a
Ethics oversight	n/a

Note that full information on the approval of the study protocol must also be provided in the manuscript.

Field-specific reporting

Please select the one below that is the best fit for your research. If you are not sure, read the appropriate sections before making your selection.

☒ Life sciences ☐ Behavioural & social sciences ☐ Ecological, evolutionary & environmental sciences

For a reference copy of the document with all sections, see nature.com/documents/nr-reporting-summary-flat.pdf

Life sciences study design

All studies must disclose on these points even when the disclosure is negative.

Sample size	Sample size was chosen in each case according to experimental design. All cell biology experiments were performed at least in 3 independent biological repeats. No statistical methods were used to pre-determine sample sizes, but our sample sizes are similar to those reported in previous publications (Enkler et al. (2023), Szentgyörgyi et al. (2024)). Data distribution was assumed to be normal, but this was not formally tested. The number of data points in each experiment is specified in the figure legends, Methods section or in Supplementary Tables 1 (Fig. 1e,f), 2 (Fig. 5e) and 4 (microscopy images and western blots).
Data exclusions	No data were excluded from the analyses.
Replication	Experiments were replicated to ensure reproducibility of the findings. For cell biology experiments, at least 3 independet experiments were performed per condition. The number of independent replicates for each experiment are specified in the respective Figure captions, Supplementary Tables and Methods sections. All attempts at replication were successful.
Randomization	All groups of experiments were performed using the same experimental conditions and protocols. Randomization was not applied. The experiment design does not require a randomized control trial. Randomization is not conventionally used during studies such as this one.

Blinding

Blinding was not used in this study as phenotypes were obvious. Biophysical assays used objective quantification methods that are not susceptible to bias, so samples were not blinded.

Reporting for specific materials, systems and methods

We require information from authors about some types of materials, experimental systems and methods used in many studies. Here, indicate whether each material, system or method listed is relevant to your study. If you are not sure if a list item applies to your research, read the appropriate section before selecting a response.

Materials & experimental systems

n/a	Involved in the study
☐	☒ Antibodies
☐	☒ Eukaryotic cell lines
☒	☐ Palaeontology and archaeology
☒	☐ Animals and other organisms
☒	☐ Clinical data
☒	☐ Dual use research of concern
☒	☐ Plants

Methods

n/a	Involved in the study
☒	☐ ChIP-seq
☐	☒ Flow cytometry
☒	☐ MRI-based neuroimaging

Antibodies

Antibodies used

All antibodies are described in the manuscript. The collowing antibodies were used in this study: polyclonal anti-PDIA6 (GeneTex GTX33397, 1:200 for immunostaining, 1:1000 for western blotting), monoclonal anti-GRP78/BiP (Invitrogen 1H11-1H7, lot: XI3695781, 1:200 for immunostaining, 1:1000 for western blotting), polyclonal anti-calreticulin (Proteintech 27298-1-AP, 1:200 for immunostaining), polyclonal anti-DNAJB11/ERdj3 (Thermofisher #15484-1-AP, 1:200 for immunostaining), monoclonal anti-Grp94 (1:200, Enzo SPA-850, lot: 103418), polyclonal anti-PDIA1 (Genetex GTX2279, 1:200 for immunostaining), polyclonal anti-calnexin (Enzo ADI-SPA-860, 1:200 for immunostaining), monoclonal anti-myc clone 9E10 (1:2000, Sigma M4439, lot: 029M4849V, 1:2000 for western blotting), monoclonal anti-tubulin primary antibody (Sigma T5168, lot: 0000242797, 1:2000 for western blotting), monoclonal anti pro/insulin (D3E7) (Invitrogen MA1-83256, lot: 160454, 1:1000 for western blotting), monoclonal anti pro/insulin (Cell Signaling C27C9, lot: 10, 1:2000 for western blotting), polyclonal anti-HaloTag (Promega G928A, 1:250 for immunostaining). HRP-conjugated goat anti-mouse IgG (H+L) secondary antibody (Thermo Fisher Scientific 31430, 1:10'000) and polyclonal HRP-conjugated goat-anit-rabbit IgG (Thermo Fisher Scientific 31460, 1:10'000) were used (incubated for 1 h at room temperature) to detect bound antibodies with Blotting detction kit WesternBrightTM ECL (advansta K-12045-D50). AlexaFluor 488 goat anti-rabbit IgG(H+L) (Invitrogen A-11034), AlexaFluor 633 goat anti-mouse IgG (H+L) (Invitrogen A-21052), AlexaFluor 488 goat anti-rat IgG(H+L). AlexaFluor 633 goat anti-rabbit IgG (H+L) (Invitrogen A-21071), AlexaFluor 488 donkey anti-rat IgG(H+L) (Invitrogen A-21208) secondary antibodies.

Validation

All primary antibodies are commercially available and validated by the manufacturer. Anti-PDIA6 (GeneTex GTX33397) was validated by the company for WB and ICC/IF, also in HeLa cells (https://www.genetex.com/Product/Detail/NTH1-antibody/GTX30397). Anti-GRP78/BiP (Invitrogen 1H11-1H7) was validated by the company for WB and ICC/IF also in various cell lines, including HeLa cells (https://www.thermofisher.com/antibody/product/GRP78-Antibody-clone-1H11-1H7-Monoclonal/MA5-27686) and verified by Cell treatment to ensure that the antibody binds to the antigen stated by Thermo Fisher Scientific. Anti-calreticulin (Proteintech 27298-1-AP) was vaildated by the company for WB, IHC and IF for various cell lines, including HeLA cells. (https://www.ptglab.com/products/Calreticulin-Antibody-27298-1-AP.htm). Anti-DNAJB11/ERdj3 (1:200, Thermofisher #15484-1-AP) was vaildated by the company for WB, IHC, ICC/IF and IP in various cell lines, inclluding HeLa cells (https://www.thermofisher.com/antibody/product/DNAJB11-Antibody-Polyclonal/15484-1-AP). Anti-Grp94 (1:200, Enzo SPA-850) was validated by the company for ICC, IF, IHC, IP and WB (https://www.enzo.com/product/grp94-monoclonal-antibody-9g10/). Anti-tubulin (Sigma) was validated by the company for WB (https://www.sigmaaldrich.com/CH/de/product/sigma/t5168?utm_source=google&utm_medium=cpc&utm_campaign=21480226247&utm_content=164812866133&gad_source=1&gbraid=0AAAAAD8kLQQjTjLXr-CDvHRQAEVius5d5&gclid=Cj0KCQiAwtu9BhC8ARIsAI9JHam8NWtk38p5nlo5XYMGCs7xO7_1C9kEbm6-uwbuNgjAlpP2V0yRswgaAnBzEALw_wcB). Anti-HaloTag antibody (Promega) was vaildated by the company for WB, IF, Pulldown (https://ch.promega.com/products/protein-detection/primary-and-secondary-antibodies/anti-halotag-pab/?catNum=G9281). Anti-myc clone 9E10 (Sigma 9E10) was validated by the company for for WB, IHC, ICC/IF and IP (https://www.sigmaaldrich.com/CH/de/product/sigma/m4439). Anti-tubulin antibody (1:2000, Sigma T5168) was validated by the company for IF and WB (https://www.sigmaaldrich.com/CH/de/product/sigma/t5168). Anti-pro/insulin clone D3E7 (5B6/6) (Invitrogen MA1-83256) was validated by the company for WB, IHV and ELISA (https://www.thermofisher.com/antibody/product/Insulin-Proinsulin-Antibody-clone-D3E7-5B6-6-Monoclonal/MA1-83256). It recognizes both insulin and proinsulin, but does not react with free C-peptide.

Eukaryotic cell lines

Policy information about cell lines and Sex and Gender in Research

Cell line source(s)

HeLa CCL2 (ATCC CCL-2) and HEK293A (ATCC CVCL-6910) kind gift of Prof. Dr. Martin Spiess. U2OS (ATCC HTB-96) kind gift of Prof. Dr. Mike Hall. INS-1 832/13 (SCC207) were purchased from Sigma Aldrich.

Authentication

Standard cell lines, HeLa CCL2 and HEK 293 authenticated by ATCC. Recentyl (2021), the cell lines' identities authenticated by

Authentication

STR analysis Microsynth AG (Balgach Switzerland). U2OS cells were not authenticated.

Mycoplasma contamination

We routinely test our cell lines for mycoplasma contamination. All cell lines were negative.

Commonly misidentified lines (See ICLAC register)

No commonly misidentified cell lines were used.

Plants

Seed stocks

Report on the source of all seed stocks or other plant material used. If applicable, state the seed stock centre and catalogue number. If plant specimens were collected from the field, describe the collection location, date and sampling procedures.

Novel plant genotypes

Describe the methods by which all novel plant genotypes were produced. This includes those generated by transgenic approaches, gene editing, chemical/radiation-based mutagenesis and hybridization. For transgenic lines, describe the transformation method, the number of independent lines analyzed and the generation upon which experiments were performed. For gene-edited lines, describe the editor used, the endogenous sequence targeted for editing, the targeting guide RNA sequence (if applicable) and how the editor was applied.

Authentication

Describe any authentication procedures for each seed stock used or novel genotype generated. Describe any experiments used to assess the effect of a mutation and, where applicable, how potential secondary effects (e.g. second site T-DNA insertions, mosiacism, off-target gene editing) were examined.

Flow Cytometry

Plots

Confirm that:

☒ The axis labels state the marker and fluorochrome used (e.g. CD4-FITC).

☒ The axis scales are clearly visible. Include numbers along axes only for bottom left plot of group (a 'group' is an analysis of identical markers).

☒ All plots are contour plots with outliers or pseudocolor plots.

☒ A numerical value for number of cells or percentage (with statistics) is provided.

Methodology

Sample preparation

HEK 293 cells were seeded at 800’000 cells/well and transfected after 24 h with either PDIA6-GFP WT, PDIA6-GFP 5x linker mutant or mock-transfected. Two days posttransfection, cells were trypsinized, fixed, permeabilized and incubated for 30 min with the PROTEASTAT dye at 1:1250 dilution in PBS with 2% Fetal Bovine Serum and 0.05 uM EDTA.

Instrument

BD LSR Fortessa Analyzer

Software

FlowJo Version 10.9.0

Cell population abundance

GFP intensity of 30'000 cells were measured per sample (single cells were 86-94% of the initial cell population).

Gating strategy

To discard the cell debri from the intial cell population FCS/SSC gating was applied. Single cells were determined by SSC-A/SSC-W and by FCS-A/FCS-H gating. From the single cell populations GFP+ cells were gted by GFP-A/SSC-A. From the GFP+ populations mCherryü cells were gated by a mCherry-A/SSC-A and the GFP+mCherry+ cells were counted in each sample.

☒ Tick this box to confirm that a figure exemplifying the gating strategy is provided in the Supplementary Information.
