# Supplementary material for: A multichaperone condensate enhances protein folding in the endoplasmic reticulum
Source: Nat Cell Biol. 2025 Aug 11;27(9):1422–30. doi: 10.1038/s41556-025-01730-w (PMC12431857; doi:10.1038/s41556-025-01730-w)

Unprocessed Blots of Fig. 5c (1/3)

The content of Fig. 5c is highlighted by red boxes. Blots representative of three biological repetitions. Unprocessed image of the blots presented in the manuscrip and merged image with molecular weight marker.

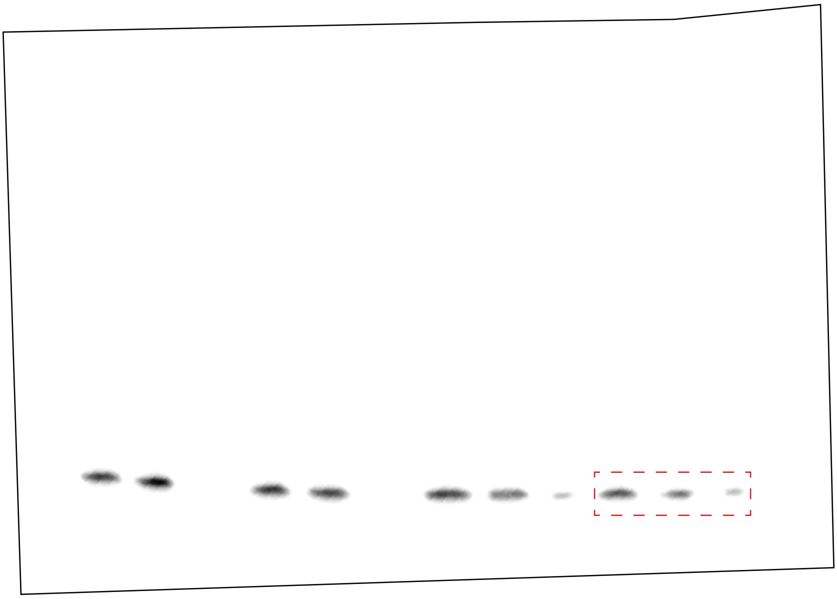

PDIA6 transf. – WT 5x – WT 5x

MW
[kDa]

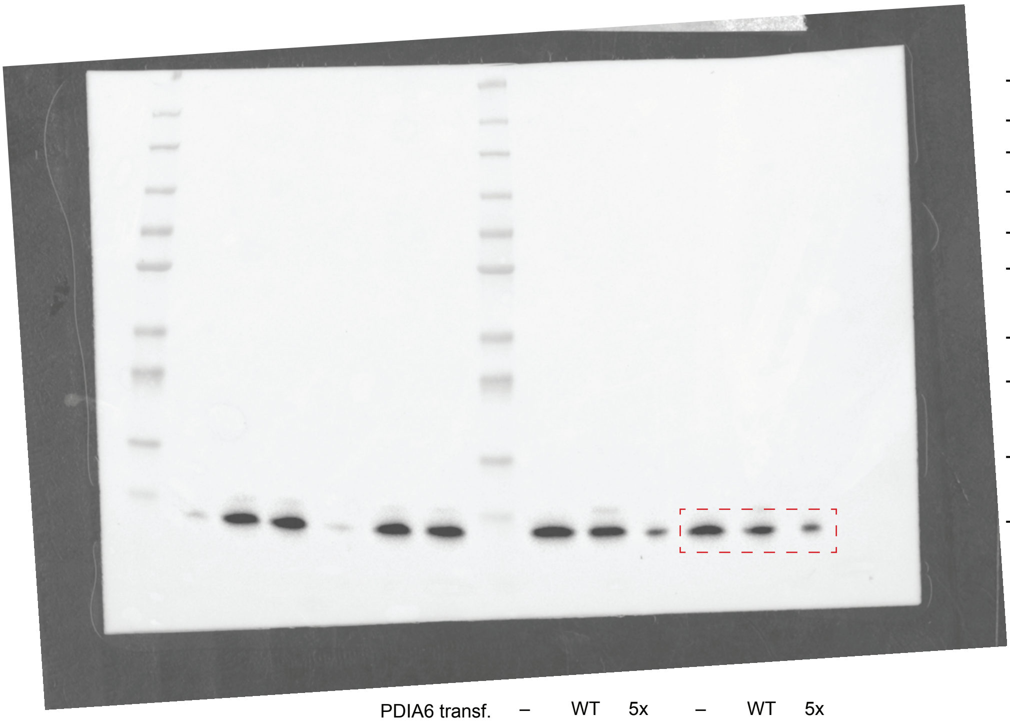

insulin

270
175
130
95
66
52
37
30
16
6.5

PDIA6 transf. – WT 5x – WT 5x

Unprocessed Blots of Fig. 5c (2/3)

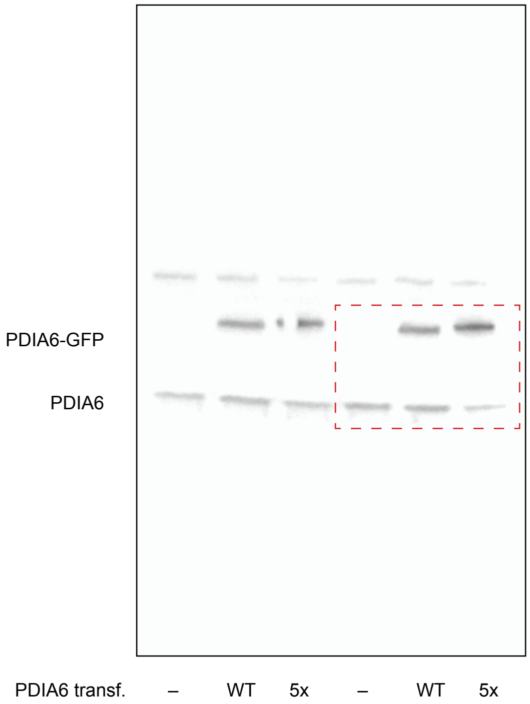

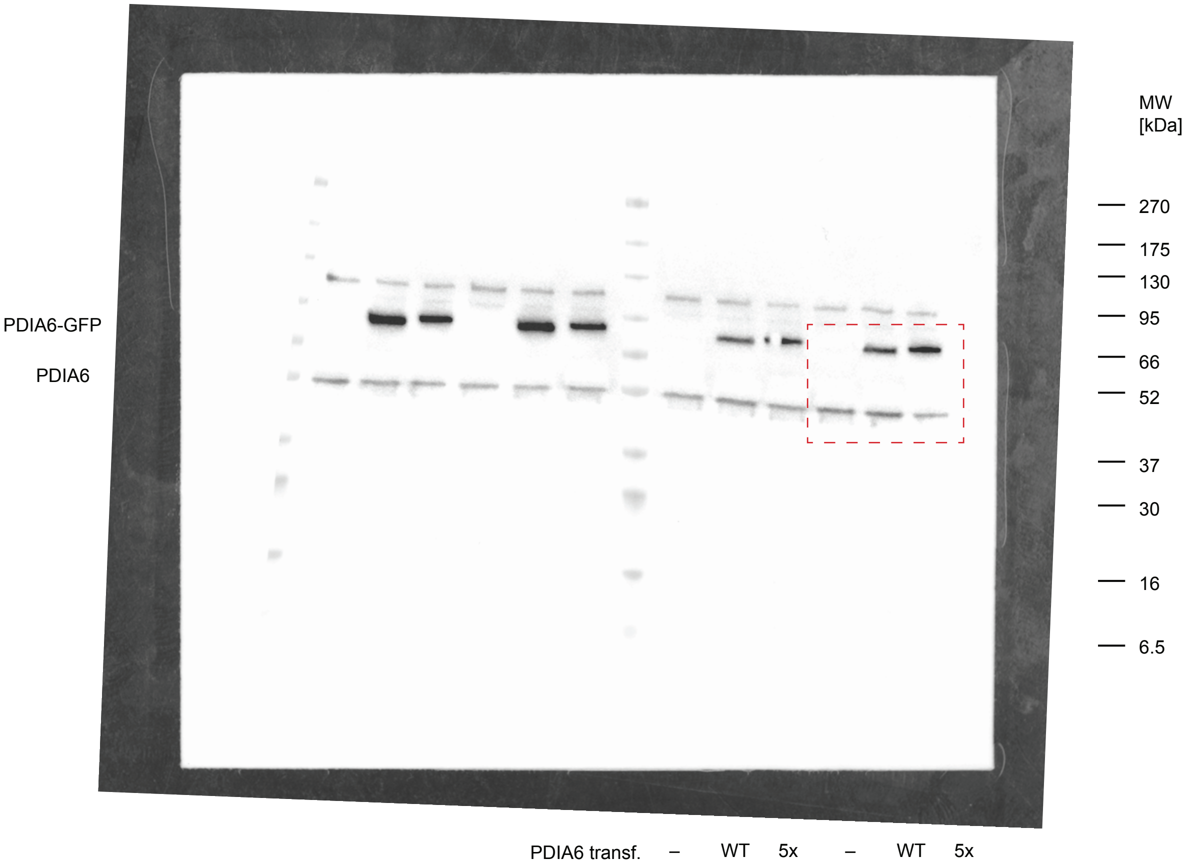

Unprocessed Blots of Fig. 5c (3/3)

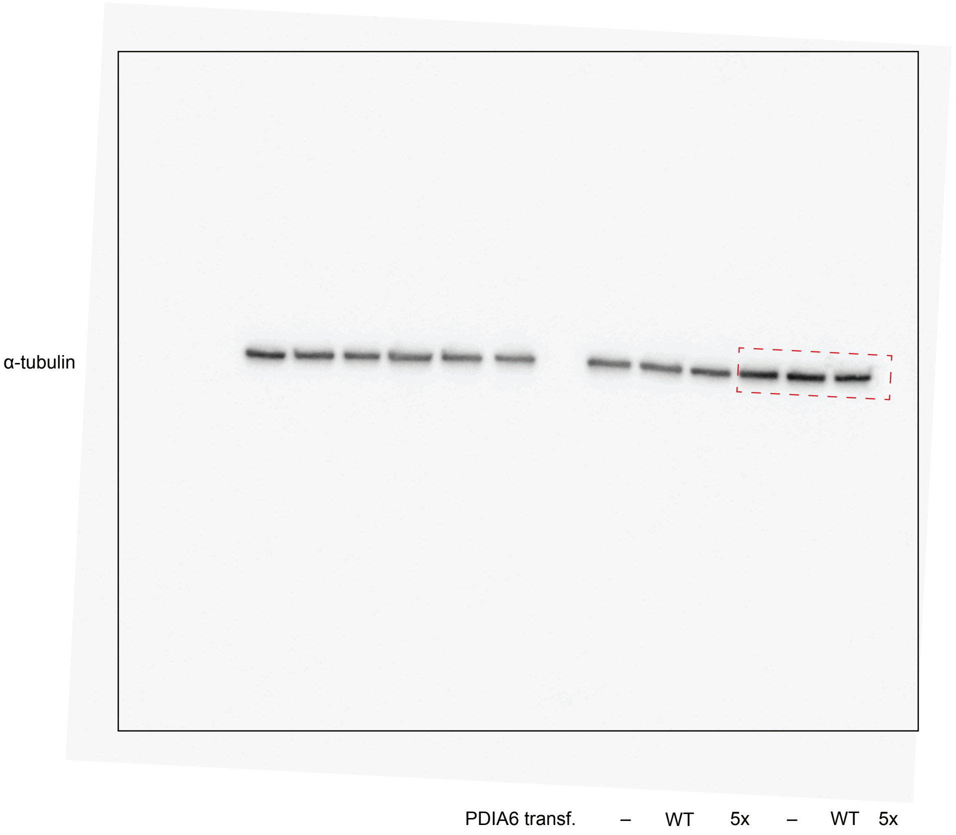

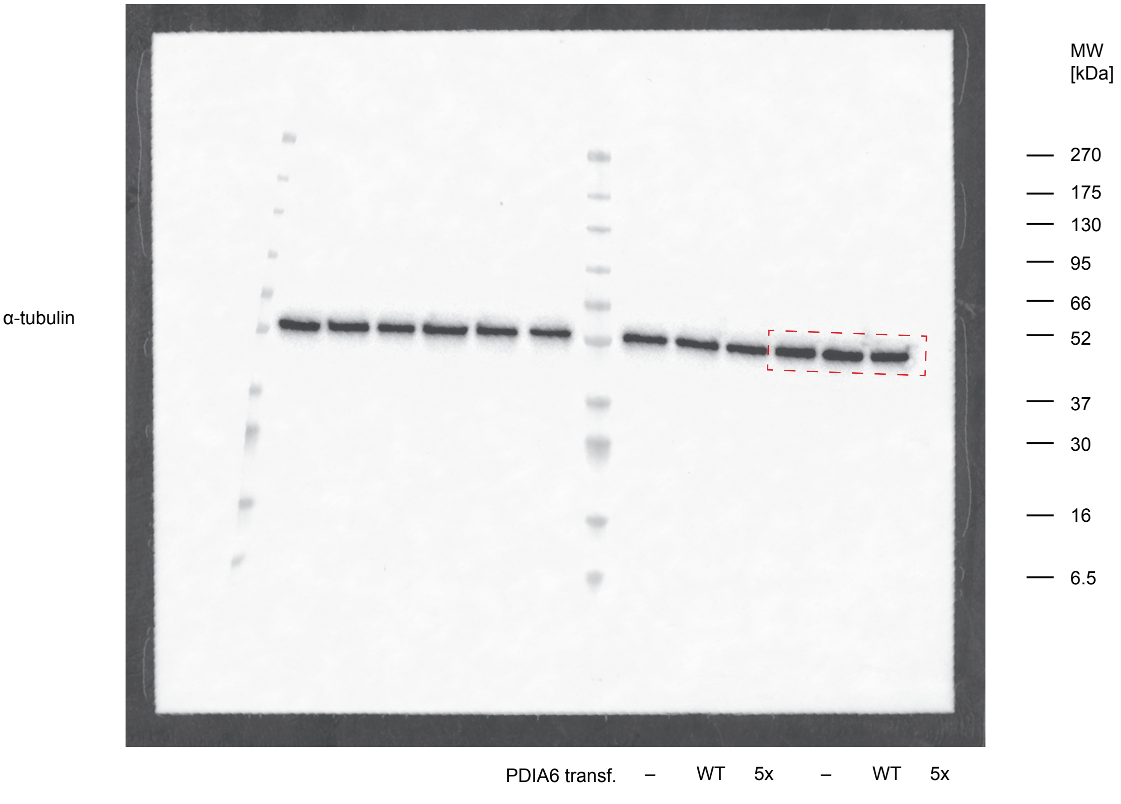

Unprocessed Blots of Fig. 5d (1/2)

The content of Fig. 5d is highlighted by red boxes. Blots representative of three biological repetitions. PDIA6 expression was probed on a different western blot than proinsulin and α-tubulin, same amount of the same lysate was used. Unprocessed image of the blots presented in the manuscript and merged image with molecular weight marker.

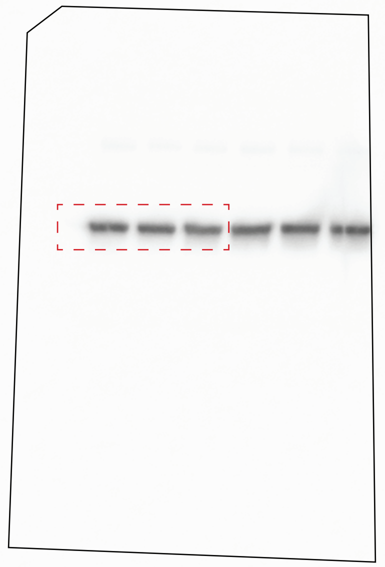

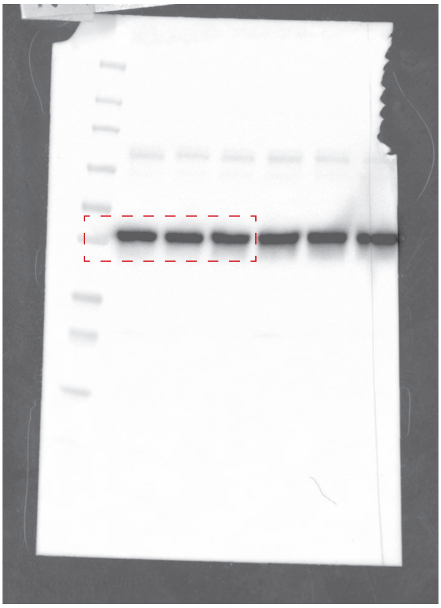

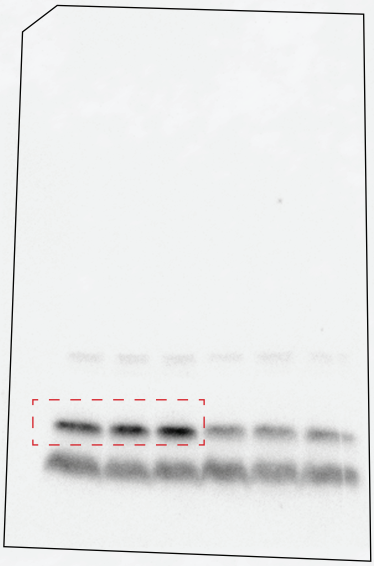

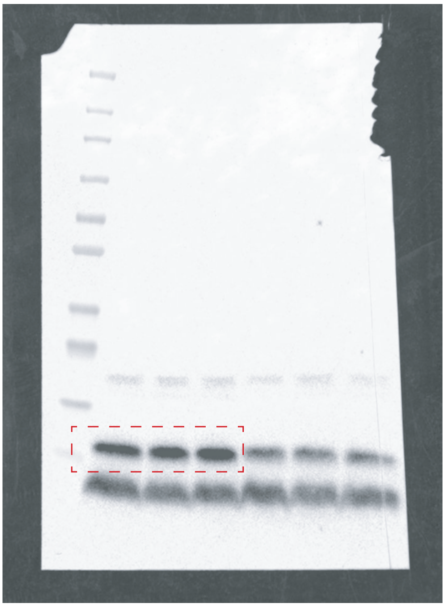

Unprocessed Blots of Fig. 5d (2/2)

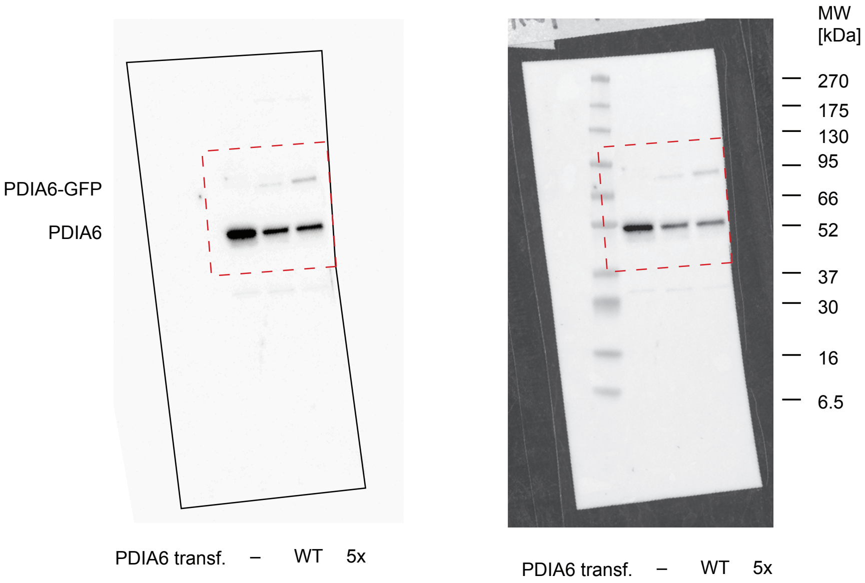

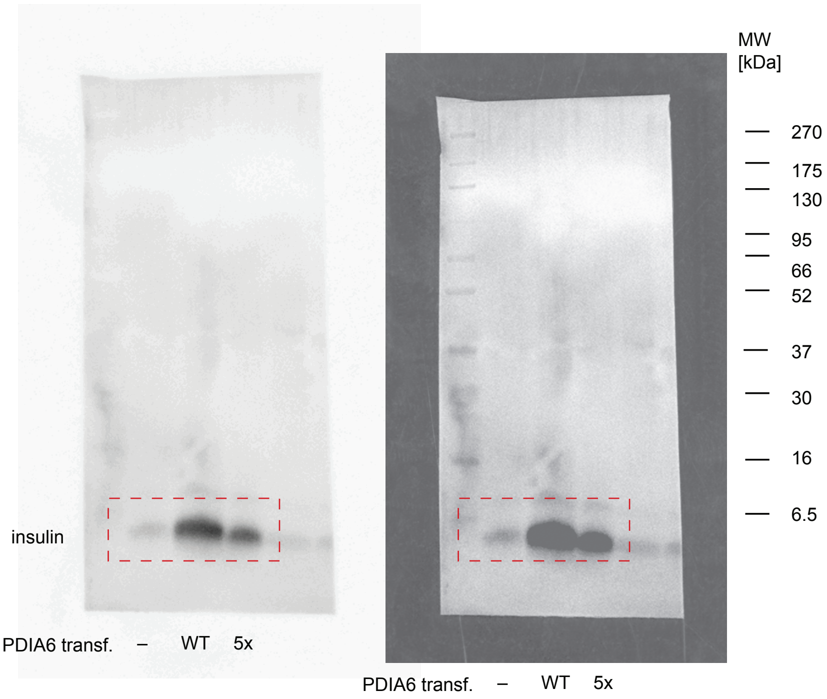

Supplement: Supplementary file 12 — Unprocessed western blots. [file 41556_2025_1730_MOESM12_ESM.pdf]
