# Supplementary material for: A multichaperone condensate enhances protein folding in the endoplasmic reticulum
Source: Nat Cell Biol. 2025 Aug 11;27(9):1422–30. doi: 10.1038/s41556-025-01730-w (PMC12431857; doi:10.1038/s41556-025-01730-w)

Unprocessed Blots of Extended Data Fig. 2b (1/2)

The content of Extended Data Fig. 2b is highlighted by red boxes. Blots representative of three biological repetitions. Unprocessed image of the blots presented in the manuscript and merged image with molecular weight marker.

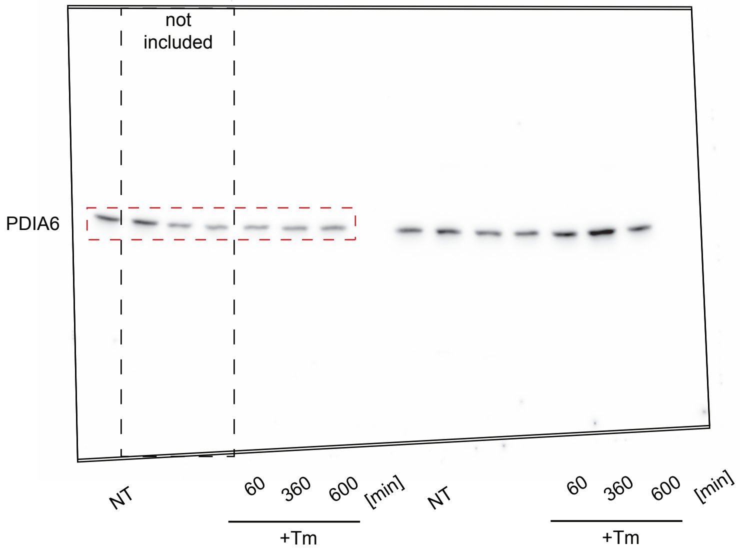

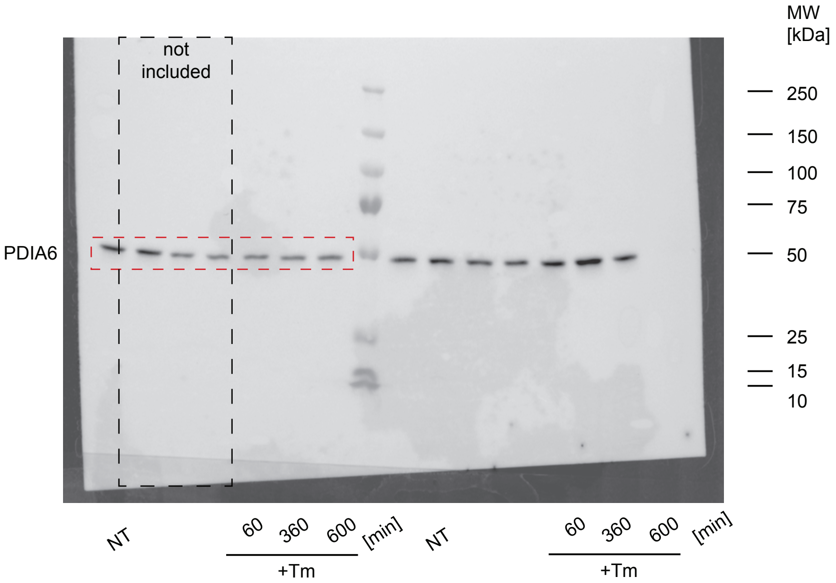

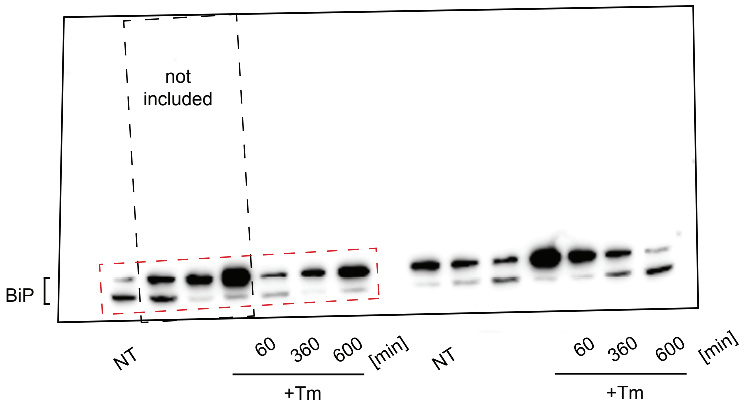

Unprocessed Blots of Extended Data Fig. 2b (2/2)

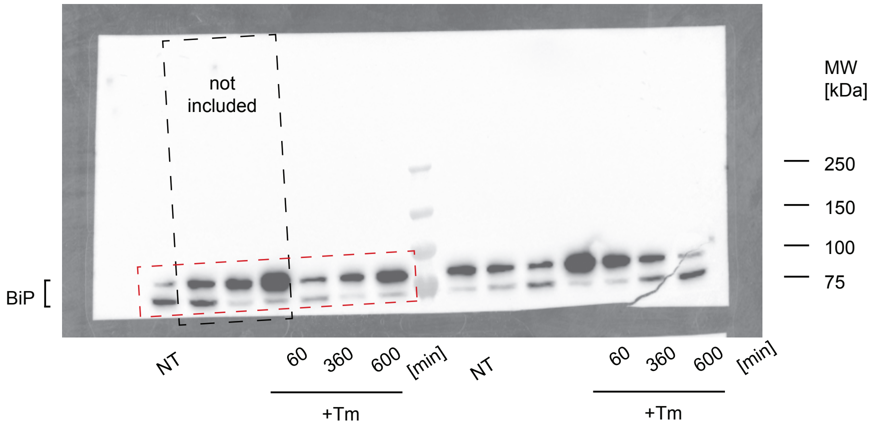

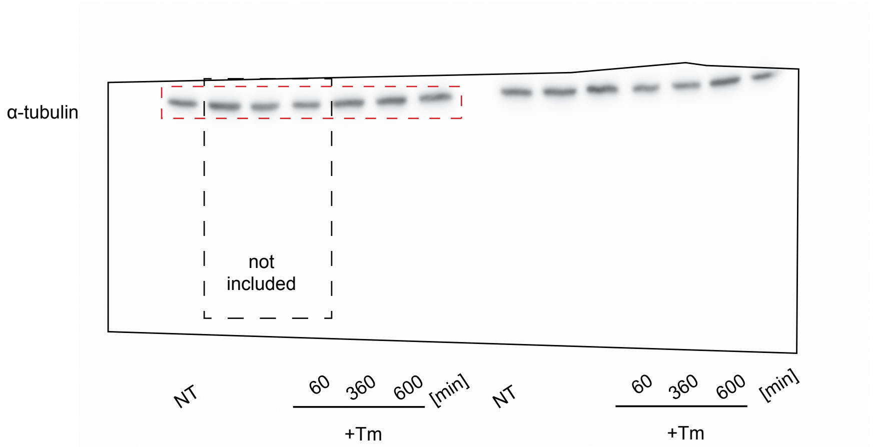

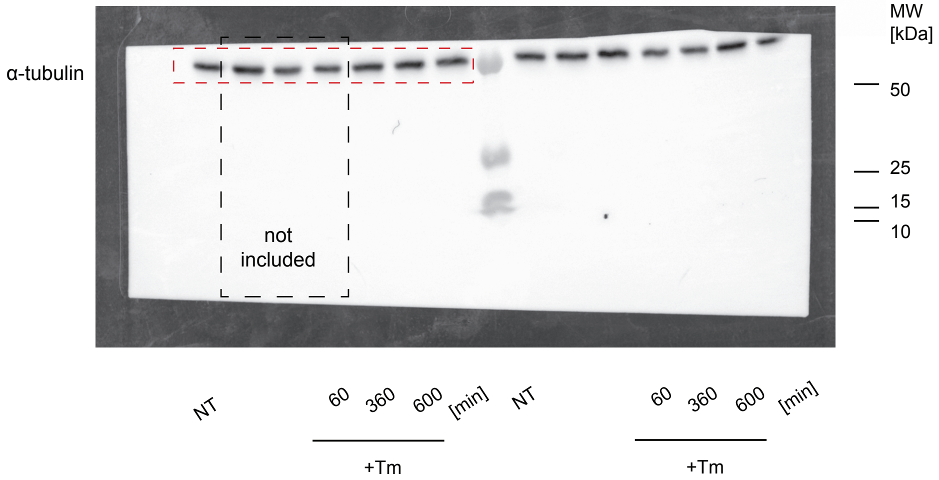

Supplement: Supplementary file 15 — Unprocessed western blots. [file 41556_2025_1730_MOESM15_ESM.pdf]
