# Supplementary material for: A multichaperone condensate enhances protein folding in the endoplasmic reticulum
Source: Nat Cell Biol. 2025 Aug 11;27(9):1422–30. doi: 10.1038/s41556-025-01730-w (PMC12431857; doi:10.1038/s41556-025-01730-w)

# Unprocessed Blot of Fig. 7l

The content of Extended Data Fig. 7l is highlighted by a red box. Blot representative of three biological repetitions.

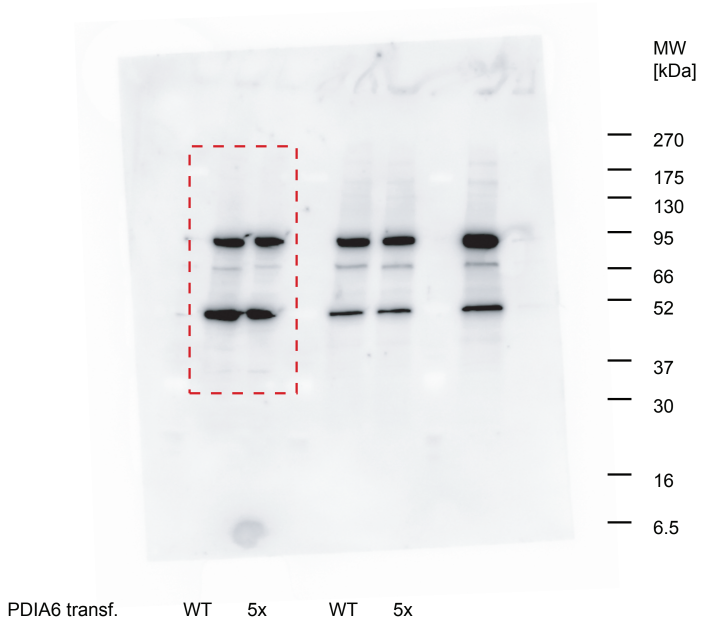

Supplement: Supplementary file 18 — Unprocessed western blots. [file 41556_2025_1730_MOESM18_ESM.pdf]
