# Supplementary material for: A multichaperone condensate enhances protein folding in the endoplasmic reticulum
Source: Nat Cell Biol. 2025 Aug 11;27(9):1422–30. doi: 10.1038/s41556-025-01730-w (PMC12431857; doi:10.1038/s41556-025-01730-w)

Unprocessed Blots of Extended Data Fig. 10b (1/2)

The content of Extended Data Fig. 10b is highlighted by red boxes. Blots representative of three biological repetitions. PDIA6 expression was probed on a different western blot than proinsulin and α-tubulin, same amount of the same lysate was used. Unprocessed image of the blots presented in the manuscript and merged image with molecular weight marker.

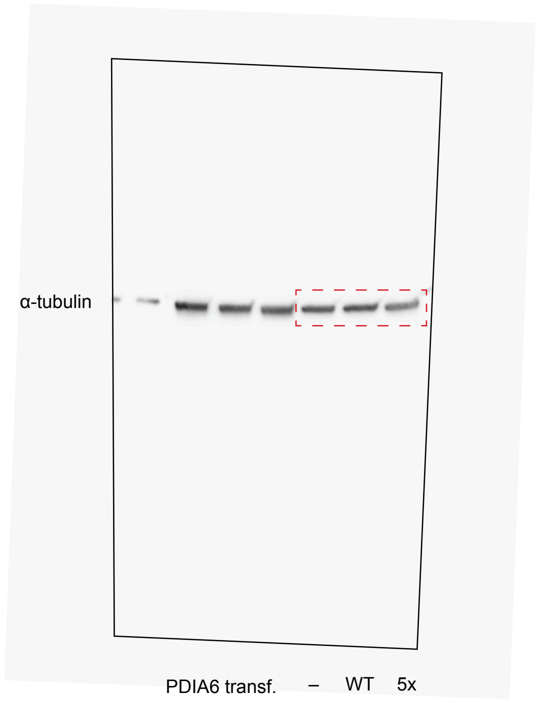

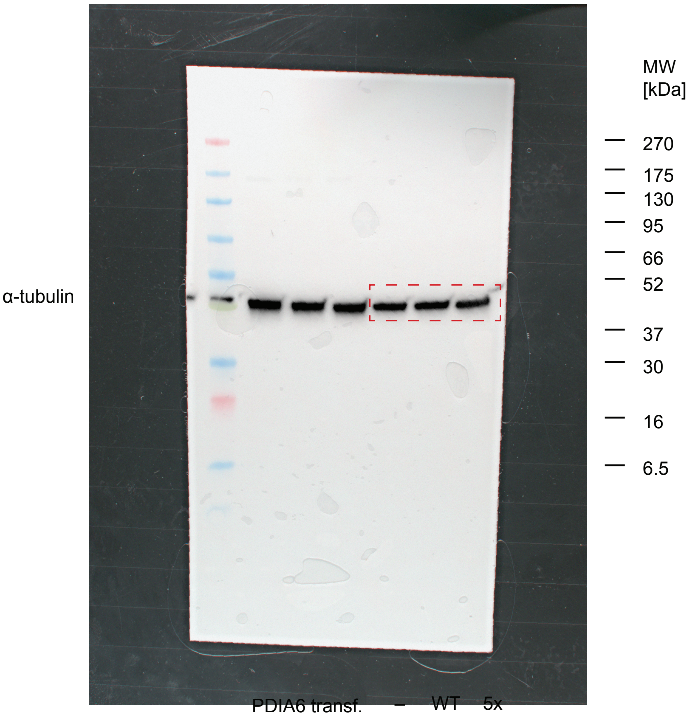

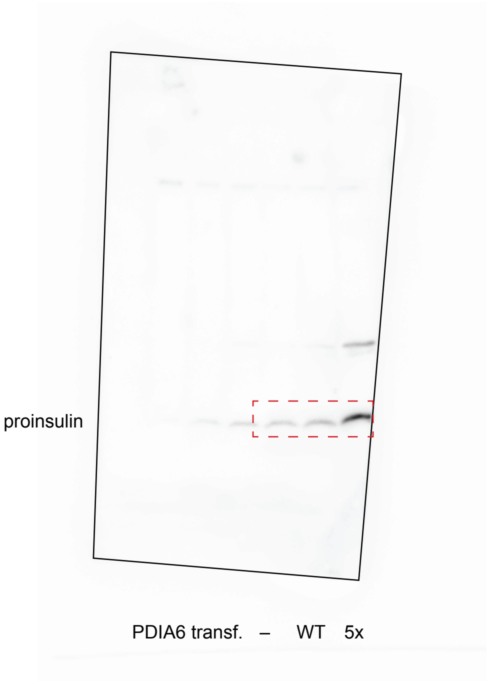

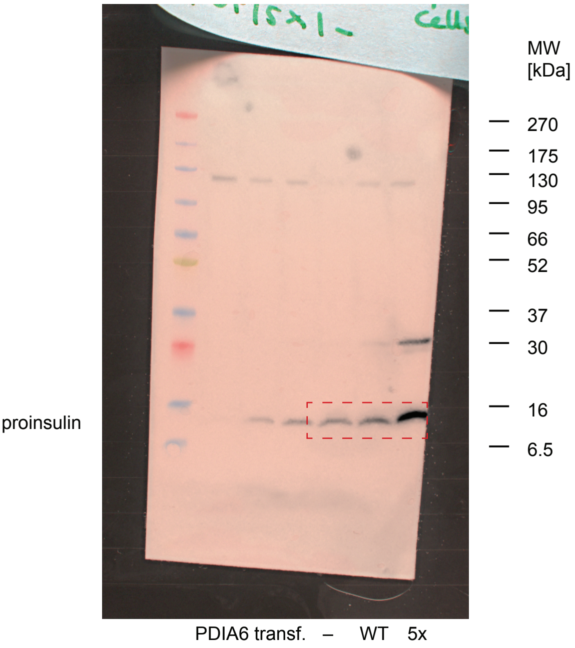

Unprocessed Blots of Extended Data Fig. 10b (2/2)

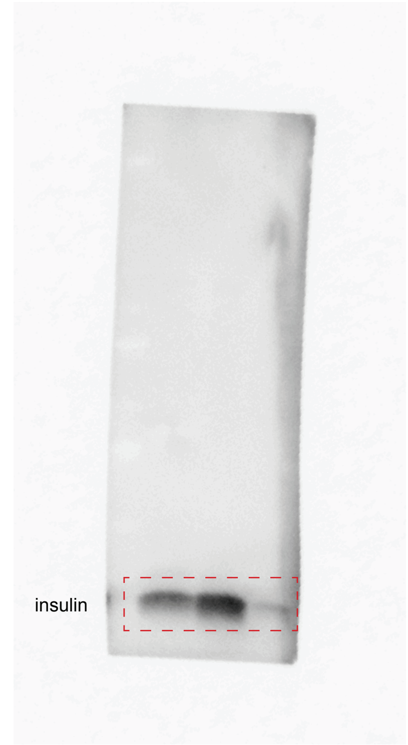

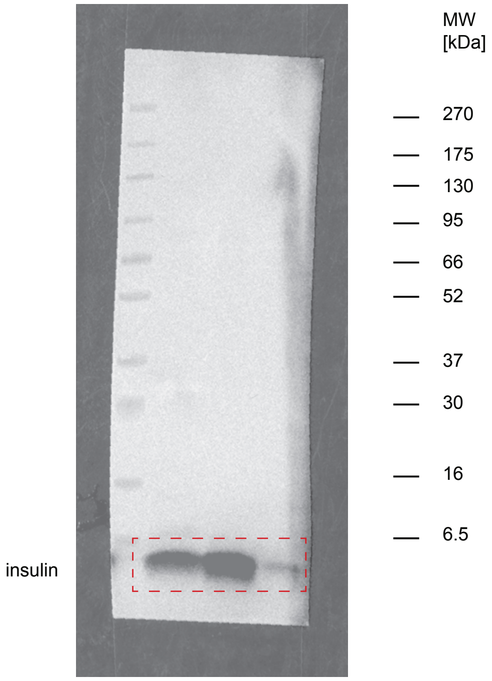

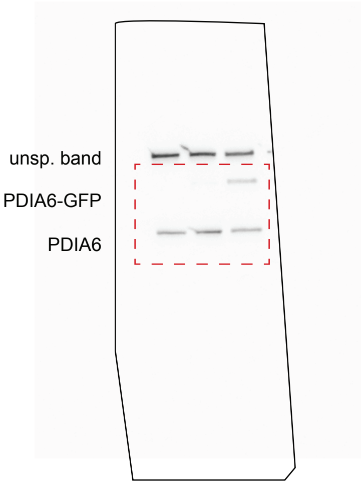

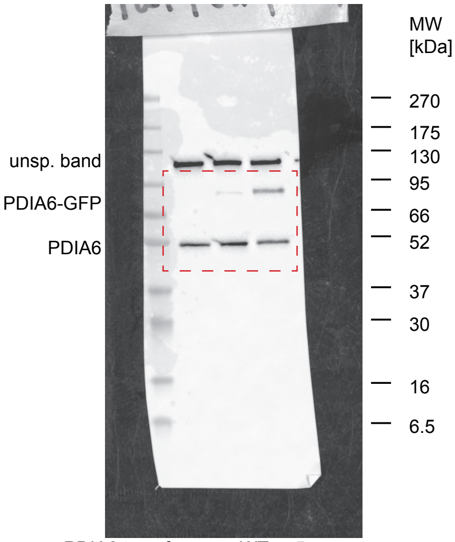

Unprocessed Blots of Extended Data Fig. 10l (1/2)

The content of Extended Data Fig. 10l is highlighted by red boxes. Blots representative of three biological repetitions. A new batch of anti-PDIA6 antibody was used, resulting in a strong unspecific band at ~100 kDa. Unprocessed image of the blots presented in the manuscript. The molecular marker was not present on the blot since the blot was cut to probe other parts with different antibodiss and was scanned seperately.

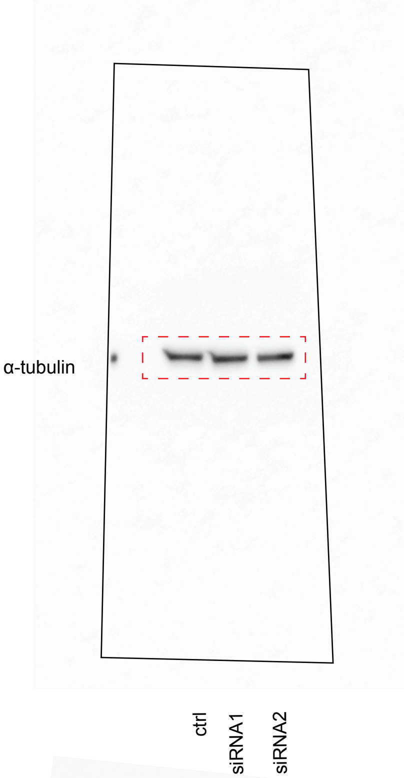

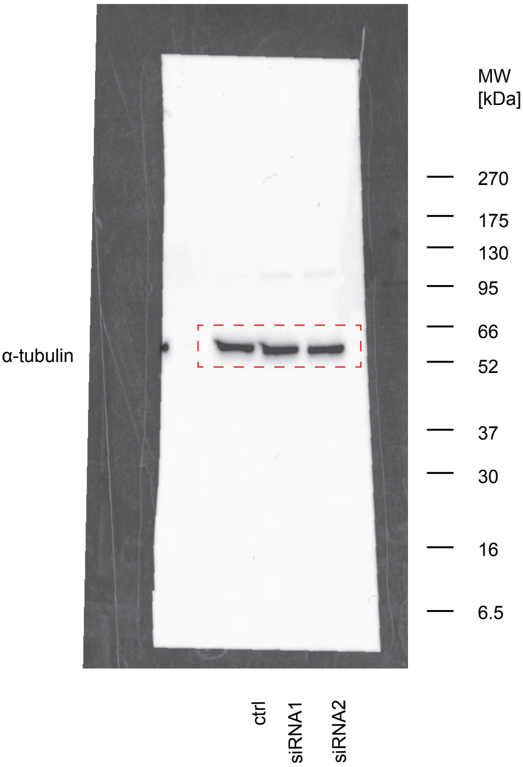

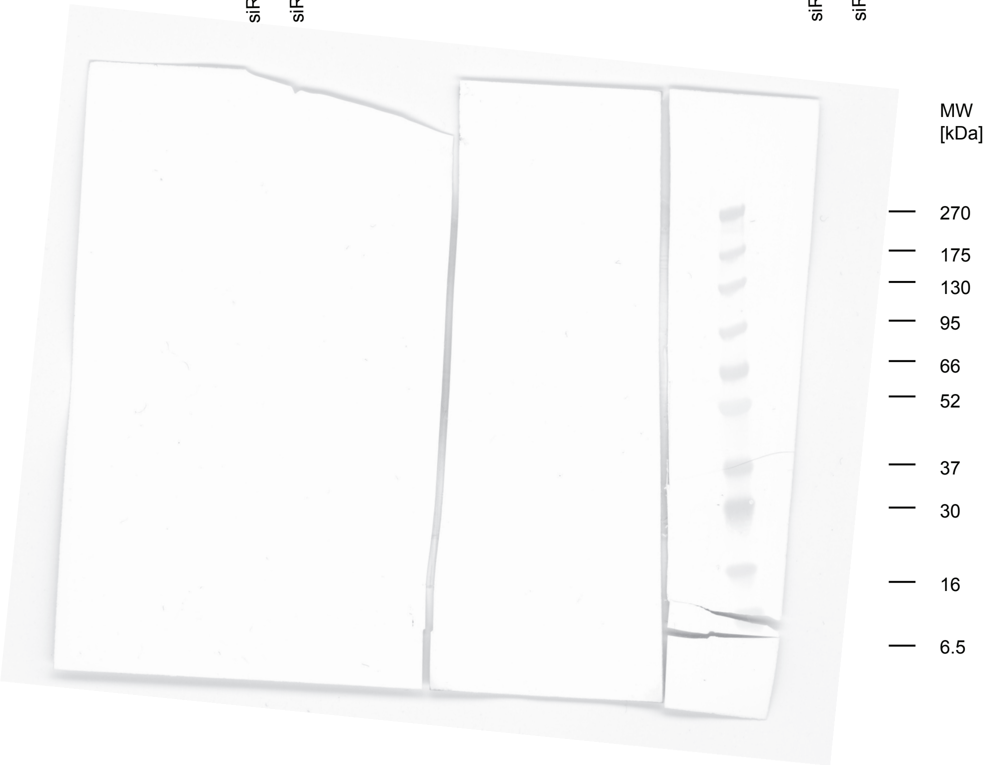

Unprocessed Blots of Extended Data Fig. 10l (2/2)

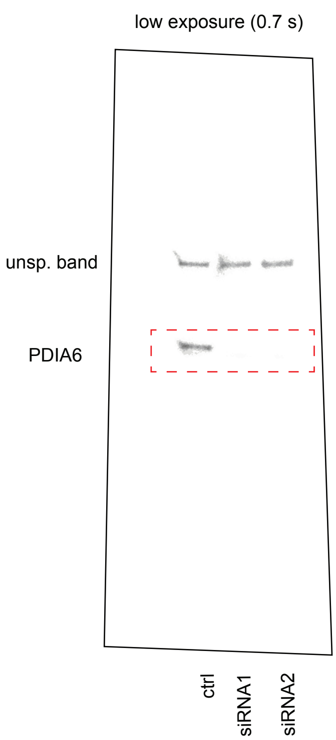

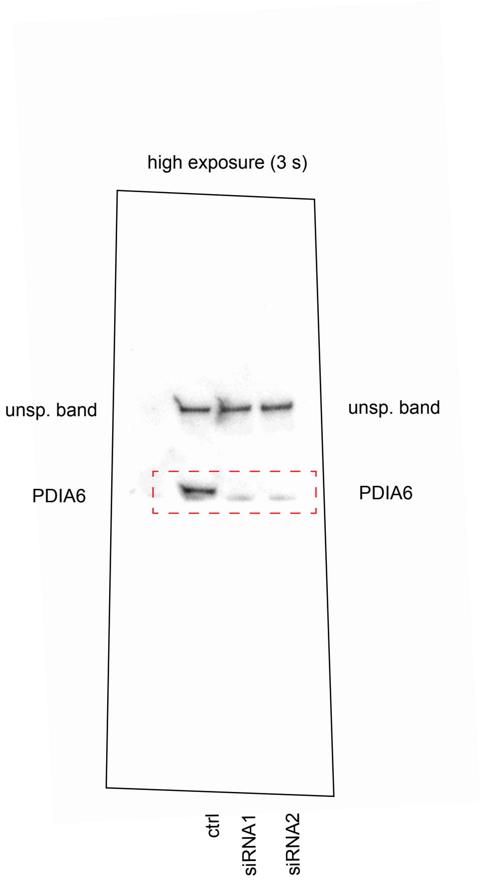

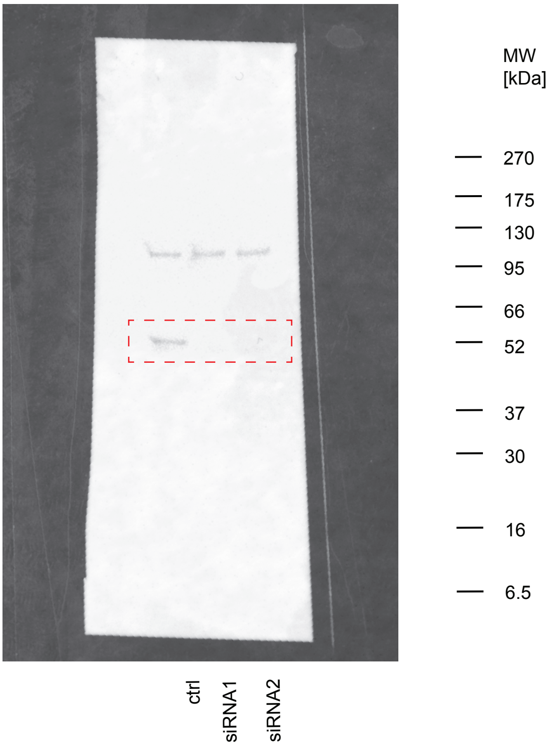

Supplement: Supplementary file 21 — Unprocessed western blots. [file 41556_2025_1730_MOESM21_ESM.pdf]
